# Genome-wide systematic characterization of the *NRT2* gene family and its expression profile in wheat (*Triticum aestivum* L.) during plant growth and in response to nitrate deficiency

**DOI:** 10.1186/s12870-023-04333-5

**Published:** 2023-07-07

**Authors:** Qing-Yan Deng, Jiang-Tao Luo, Jian-Min Zheng, Wen-Fang Tan, Zong-Jun Pu, Fang Wang

**Affiliations:** 1 Crop Research Institute, Sichuan Academy of Agricultural Sciences, Chengdu, 610066 Sichuan China; 2 Environment-Friendly Crop Germplasm Innovation and Genetic Improvement Key Laboratory of Sichuan Province, Chengdu, 610066 Sichuan China; 3 Key Laboratory of Wheat Biology and Genetic Improvement on Southwestern China (Ministry of Agriculture and Rural Affairs of P.R.C.), Chengdu, Sichuan 610066 China

**Keywords:** Wheat, Nitrogen use efficiency (NUE), NRT2, Low nitrogen stress, Transcriptome analysis

## Abstract

**Background:**

Wheat (*Triticum aestivum* L.) is a major cereal crop that is grown worldwide, and it is highly dependent on sufficient N supply. The molecular mechanisms associated with nitrate uptake and assimilation are still poorly understood in wheat. In plants, NRT2 family proteins play a crucial role in NO_3_^–^ acquisition and translocation under nitrate limited conditions. However, the biological functions of these genes in wheat are still unclear, especially their roles in NO_3_^–^ uptake and assimilation.

**Results:**

In this study, a comprehensive analysis of wheat *TaNRT2* genes was conducted using bioinformatics and molecular biology methods, and 49 *TaNRT2* genes were identified. A phylogenetic analysis clustered the *TaNRT2* genes into three clades. The genes that clustered on the same phylogenetic branch had similar gene structures and nitrate assimilation functions. The identified genes were further mapped onto the 13 wheat chromosomes, and the results showed that a large duplication event had occurred on chromosome 6. To explore the *TaNRT2* gene expression profiles in wheat, we performed transcriptome sequencing after low nitrate treatment for three days. Transcriptome analysis revealed the expression levels of all *TaNRT2* genes in shoots and roots, and based on the expression profiles, three highly expressed genes (*TaNRT2-6A.2*, *TaNRT2-6A.6*, and *TaNRT2-6B.4*) were selected for qPCR analysis in two different wheat cultivars (‘Mianmai367’ and ‘Nanmai660’) under nitrate-limited and normal conditions. All three genes were upregulated under nitrate-limited conditions and highly expressed in the high nitrogen use efficiency (NUE) wheat ‘Mianmai367’ under low nitrate conditions.

**Conclusion:**

We systematically identified 49 *NRT2* genes in wheat and analysed the transcript levels of all *TaNRT2s* under nitrate deficient conditions and over the whole growth period. The results suggest that these genes play important roles in nitrate absorption, distribution, and accumulation. This study provides valuable information and key candidate genes for further studies on the function of *TaNRT2*s in wheat.

**Supplementary Information:**

The online version contains supplementary material available at 10.1186/s12870-023-04333-5.

## Introduction

Nitrogen (N) is the second most important crop input factor after water. It is also an important component of most biomacromolecules and many secondary and signaling compounds in plants, such as proteins, nucleic acids, cell wall components, phytohormones, and vitamins [1, 2]. Therefore, nitrogen deficiency could severely limit plant growth and development. This is particularly true for wheat. Wheat plants do not establish symbiotic associations with N_2_-fixing microbes [3]. Therefore, chemical fertilizers have historically been used to maintain or increase crop yields. However, these chemical fertilizers have been mismanaged, resulting in environmental pollution and decreased nutrient-use efficiency (NUE) [4]. For example, only one-third of the applied nitrogen is utilized by wheat, which suggests that there is potential for increasing its NUE [5]. The remaining N is released into the environment through leaching and volatilization [6]. This means that low wheat NUE and excess N fertilizer applications are aggravate environmental pollution and cause ecological deterioration [7, 8]. Therefore, improving the NUE will improve the sustainability of wheat production. However, achieving greater NUE is challenged by the complexity of the trait, which is comprised of processes associated with nitrogen uptake, transport, reduction, assimilation, translocation, and remobilization.

Nitrogen is available to plant roots in several different forms, such as NO_3_^−^, NH_4_^+^, and organic molecules, such as amino acids [9]. Nitrate is one of the most important N sources for plants. Nitrogen uptake is the first step in nitrate assimilation and can be manipulated to enhance NUE. Plants have evolved regulated, energy-dependent systems for the uptake of NO_3_^−^ that use both high- and low-affinity transporters. The nitrate transporter 1 (NRT1)/peptide transporter (PTR) family (NPF), NRT2 family, chloride channel (CLC) family, and slow anion channel (SLAC) protein family are the four protein families that play key roles in NO_3_^−^ transport [10, 11]. The NRT1 and NRT2 families have been identified as being involved in low-affinity nitrate transporter systems (LATSs) and high-affinity nitrate transporter systems (HATSs), respectively. The LATS is activated when nitrate concentrations are high (> 1 mM), whereas the HATS is activated when nitrate concentrations are low (< 1 mM) [12, 13]. The NRT2s, which are thought to be involved in the major transporter system responsible for nitrate uptake in plants, are membrane associated proteins and contribute specifically to nitrate-inducible steps.

The first NRT2 family transporters were discovered in a chlorate-resistant mutant (*crnA*) of *Aspergillus nidulans* [14, 15]. Subsequently, numerous studies have investigated the functional roles of the plant NRT2 family and important progress has been made. There are 7 *NRT2* genes in Arabidopsis [10, 16], 4 in rice [17], 4 in maize [18], 31 in rapeseed [19, 20], 13 in poplar [21], 4 in tomato [22], and 5 in wild soybean (*Glycine soja*) [23]. In Arabidopsis, four AtNRT2 transporters (AtNRT2.1, AtNRT2.2, AtNRT2.3, and AtNRT2.4) are involved in nitrate uptake. The *AtNRT2.1* and *AtNRT2.2* genes play key roles in the regulation of high-affinity NO_3_^–^ uptake and *nrt2.1nrt2.2* reduces the inducible high-affinity transport system (IHATS) by up to 80% in *Arabidopsis thaliana* [24, 25]. *AtNRT2.4* has a role in both the roots and shoots under N starvation [26] and *AtNRT2.5* is the most abundant transcript in adult plants among the seven *AtNRT2* family members after long-term nitrogen starvation [27]. Furthermore, *AtNRT2.7* is specifically highly expressed in reproductive organs, reaching a maximum in dry seeds, and AtNRT2.7 is the only NRT2 transporter located in the tonoplast [28].

In crops, the homologs of AtNRT2s have been shown to perform numerous roles in N uptake, transport, and utilization processes across all developmental stages. In rice, *OsNRT2.1* and *OsNRT2.2* share the same coding sequences (CDSs) with different 5′- and 3′-untranslated regions (UTRs) and have high similarities with maize *ZmNRT2* genes, while *OsNRT2.3* is more closely related to *AtNRT2.5*, and *OsNRT2.4* is more closely related to *AtNRT2.7* [17]. *OsNRT2.3* mRNA has been previously spliced into *OsNRT2.3a* and *OsNRT2.3b* [29]. OsNRT2.3a plays a key role in long-distance nitrate transport from root to shoot at low nitrate supply levels [30], OsNRT2.3b plays a critical role in sensing the cytosolic pH of phloem cells and increased *OsNRT2.3b* expression improves grain yield and NUE [31]. OsNRT2.4 has been shown to be a dual­affinity nitrate transporter and is required for nitrate-regulated root and shoot growth [32]. In wheat, *TaNRT2.5* is expressed in the root, leaf, embryo, and shell and can increase seed vigour, grain nitrate accumulation, and yield [33]. In maize, only ZmNRT2.1 plays a role in nitrate uptake along the root axis [34]. In summary, NRT2 homologs play key roles in nitrate uptake and utilization in plants.

Wheat (*Triticum aestivum* L.) is one of the three main cereal crops across the globe. The ability to uptake N is heavily dependent on the functional efficiency of the nitrate transporter, which is genetically determined in many crops. However, *TaNRT2* family members have not been systematically identified, and their expression has been analysed under nitrate deficiency conditions in wheat. This is due to the complexity of its genome. In this study, a genome-wide identification of *TaNRT2* members in wheat was performed. The gene structures, chromosomal locations, *cis*-elements, and conserved motifs of all *TaNRT2s* were also analysed. Furthermore, a transcriptome analysis of all *TaNRT2s* was conducted under nitrate starvation conditions. This study reveals the characteristics of *NRT2* genes in wheat and provides valuable information and candidate gene resources for future functional analyses that could be used to genetically improve the NUE of wheat.

## Results

### Identification of the *NRT2* gene family in wheat

**Fig. 1:**
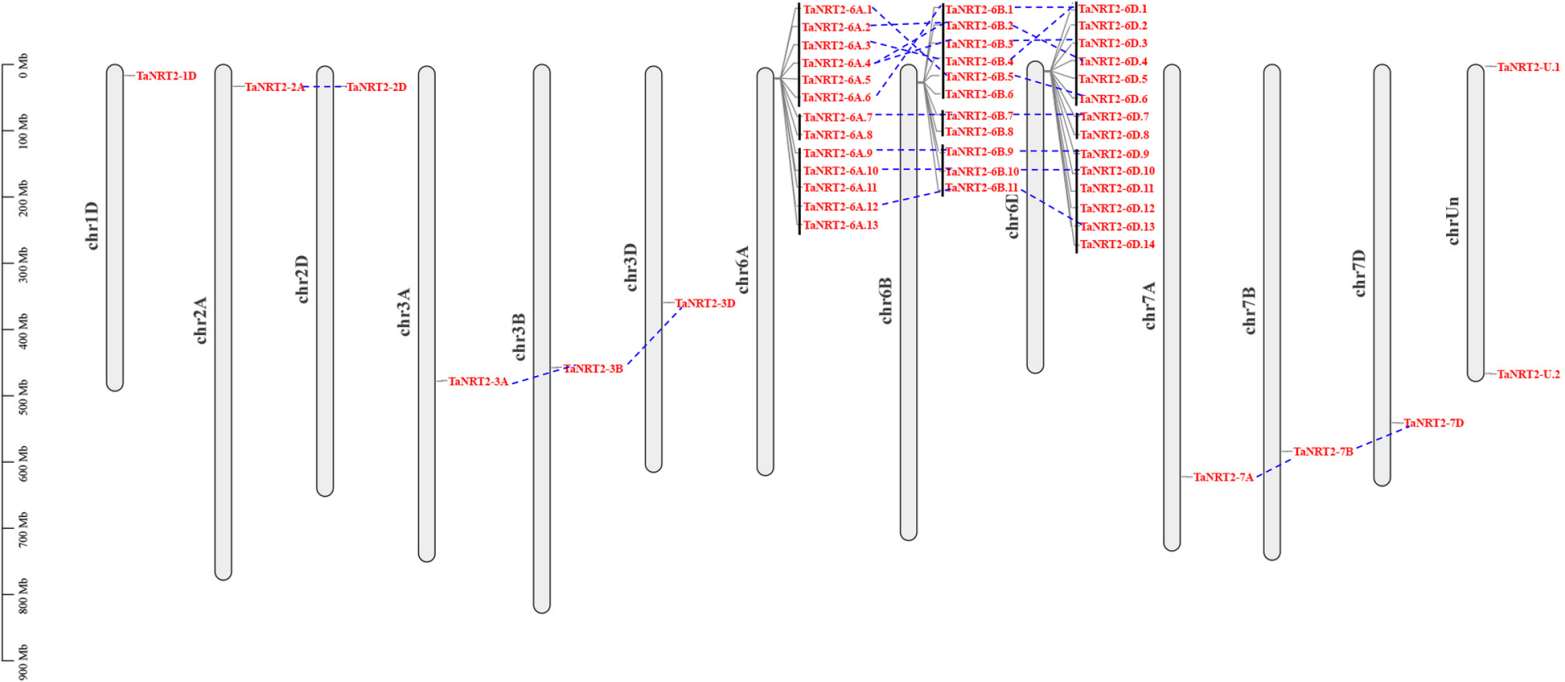
Chromosomal localization and collinearity of *TaNRT2* genes on bread wheat genome. The black line between the gene names indicated that they were tandem repeat gene pairs. Gene locations are shown by the scale. The gene location on each chromosome is represented by grey lines. The collinear relationships of *TaNRT2* genes are indicated by blue dotted lines

To identify the *NRT2* gene family in wheat, whole-genome scanning and a Blastp search were used to identify the genes that contained the conserved domain (MFS). A total of 49 *NRT2* genes were identified in the wheat genome. These consisted of 46 high-confidence genes and three low-confidence genes (Table 1). The 49 *NRT2* genes were unevenly distributed on the 13 wheat chromosomes and 38 of them were located on chromosome 6 (Fig. 1). The *TaNRT2s* on chromosome 6 showed multiple duplication to form tandemly duplicated gene clusters. There was also good collinearity among the 6A, 6B, and 6D homologous genes (Fig. 1). In addition, the characteristics of the *TaNRT2* genes, including the CDS length, protein length, molecular weight (MW), isoelectric point (pI), and predicted subcellular localization, were systematically evaluated (Table 1). The CDS lengths of the *TaNRT2* genes ranged from 780 (*TaNRT2-U.2*) to 1698 (*TaNRT2-6B.5*) and the corresponding protein lengths ranged from 259 to 565. The protein MWs ranged from 28.00 kDa (TaNRT2-U.2) to 60.98 kDa (TaNRT2-6B.5) and the average pIs of the TaNRT2 proteins ranged from 7.51 (TaNRT2-7D) to 9.77 (TaNRT2-7A). The subcellular localization prediction for TaNRT2 proteins suggested that most TaNRT2s were located on the plasma membrane.

**Table 1:** *TaNRT2* genes identified in wheat

Gene Name	Gene ID	Genomic Location	CDS Length (bp)	Amino acid Length (aa)	Molecular Weight (KDa)	pI	Predicted Subcellular Localization
TaNRT2-1D	TraesCS1D02G035700	chr1D:16504613..16506169	1557	518	55.47	8.93	plasma membrane
TaNRT2-2A	TraesCS2A02G074800	chr2A:33054150..33056031	1509	502	54.58	8.98	plasma membrane
TaNRT2-2D	TraesCS2D02G073500	chr2D:30787486..30789242	1500	499	54.12	9.14	plasma membrane
TaNRT2-3A	TraesCS3A02G254000	chr3A:475304797..475306341	1545	514	55.40	8.82	plasma membrane
TaNRT2-3B	TraesCS3B02G285900	chr3B:457633984..457635782	1545	514	55.34	8.71	plasma membrane
TaNRT2-3D	TraesCS3D02G254900	chr3D:356623041..356624585	1545	514	55.36	8.71	plasma membrane
TaNRT2-6A.1	TraesCS6A02G030700	chr6A:15727844..15729367	1524	507	54.72	8.39	plasma membrane
TaNRT2-6A.2	TraesCS6A02G030800	chr6A:15734520..15736043	1524	507	54.75	8.39	plasma membrane
TaNRT2-6A.3	TraesCS6A02G030900	chr6A:15747526..15749383	1524	507	54.72	8.39	plasma membrane
TaNRT2-6A.4	TraesCS6A02G031000	chr6A:15756560..15758437	1524	507	54.75	8.39	plasma membrane
TaNRT2-6A.5	TraesCS6A02G031100	chr6A:15765759..15767783	1524	507	55.76	8.53	plasma membrane
TaNRT2-6A.6	TraesCS6A02G031200	chr6A:15781020..15782725	1530	509	55.04	8.65	plasma membrane
TaNRT2-6A.7	TraesCS6A02G032400	chr6A:15951566..15953536	1527	508	54.62	8.70	plasma membrane
TaNRT2-6A.8	TraesCS6A02G032500	chr6A:16098637..16100163	1527	508	54.57	8.80	plasma membrane
TaNRT2-6A.9	TraesCS6A02G032800	chr6A:16357746..16359603	1524	507	54.54	7.87	plasma membrane
TaNRT2-6A.10	TraesCS6A02G032900	chr6A:16374353..16376212	1530	509	54.52	8.16	plasma membrane
TaNRT2-6A.11	TraesCS6A02G033000	chr6A:16386427..16388254	1530	509	54.59	8.14	plasma membrane
TaNRT2-6A.12	TraesCS6A02G033100	chr6A:16398961..16400795	1527	508	54.55	7.88	plasma membrane
TaNRT2-6A.13	TraesCS6A02G033200	chr6A:16408185..16410137	1527	508	54.67	7.89	plasma membrane
TaNRT2-6B.1	TraesCS6B02G044000	chr6B:26591111..26592640	1530	509	55.19	8.65	plasma membrane
TaNRT2-6B.2	TraesCS6B02G044100	chr6B:26596252..26597775	1524	507	54.76	8.53	plasma membrane
TaNRT2-6B.3	TraesCS6B02G044200	chr6B:26616491..26618567	1524	507	55.77	8.54	plasma membrane
TaNRT2-6B.4	TraesCS6B02G044300	chr6B:26625403..26626926	1524	507	54.71	8.39	plasma membrane
TaNRT2-6B.5	TraesCS6B02G044400	chr6B:26633039..26634966	1698	565	60.98	8.60	plasma membrane
TaNRT2-6B.6	TraesCS6B02G044500	chr6B:26644113..26645632	1458	485	52.94	8.70	plasma membrane
TaNRT2-6B.7	TraesCS6B02G045600	chr6B:27122861..27124387	1527	508	54.64	8.71	plasma membrane
TaNRT2-6B.8	TraesCS6B02G045700	chr6B:27169710..27171230	1521	506	54.28	8.91	plasma membrane
TaNRT2-6B.9	TraesCS6B02G046500	chr6B:27685182..27687046	1524	507	54.41	7.87	plasma membrane
TaNRT2-6B.10	TraesCS6B02G046600	chr6B:27778038..27779912	1530	509	54.55	7.90	plasma membrane
TaNRT2-6B.11	TraesCS6B02G046700	chr6B:27818480..27820351	1527	508	54.62	7.88	plasma membrane
TaNRT2-6D.1	TraesCS6D02G035600	chr6D:14618629..14620585	1530	509	55.06	8.53	plasma membrane
TaNRT2-6D.2	TraesCS6D02G035800LC	chr6D:14624460..14625647	1188	395	42.68	8.04	plasma membrane
TaNRT2-6D.3	TraesCS6D02G036100LC	chr6D:14663011..14663928	918	305	33.14	7.63	endoplasmic reticulum
TaNRT2-6D.4	TraesCS6D02G035700	chr6D:14631385..14633069	1524	507	54.73	8.39	plasma membrane
TaNRT2-6D.5	TraesCS6D02G035800	chr6D:14655066..14656589	1524	507	54.70	8.39	plasma membrane
TaNRT2-6D.6	TraesCS6D02G035900	chr6D:14679252..14680775	1524	507	54.72	8.39	plasma membrane
TaNRT2-6D.7	TraesCS6D02G037200	chr6D:15383513..15385158	1527	508	54.64	8.71	plasma membrane
TaNRT2-6D.8	TraesCS6D02G037300	chr6D:15418086..15419612	1527	508	54.51	8.80	plasma membrane
TaNRT2-6D.9	TraesCS6D02G037800	chr6D:15658356..15659879	1524	507	54.43	7.90	plasma membrane
TaNRT2-6D.10	TraesCS6D02G037900	chr6D:15696840..15698711	1530	509	54.52	8.16	plasma membrane
TaNRT2-6D.11	TraesCS6D02G038000	chr6D:15710039..15711562	1524	507	54.44	8.29	plasma membrane
TaNRT2-6D.12	TraesCS6D02G038100	chr6D:15745837..15748091	1530	509	54.62	8.22	plasma membrane
TaNRT2-6D.13	TraesCS6D02G038200	chr6D:15797524..15799374	1527	508	54.53	8.14	plasma membrane
TaNRT2-6D.14	TraesCS6D02G038300	chr6D:15807091..15808955	1527	508	54.57	7.88	plasma membrane
TaNRT2-7A	TraesCS7A02G428500	chr7A:621910950..621913739	1407	468	49.05	9.77	plasma membrane
TaNRT2-7B	TraesCS7B02G328700	chr7B:583923053..583926829	1461	486	50.75	7.95	plasma membrane
TaNRT2-7D	TraesCS7D02G420900	chr7D:540617018..540627808	1452	483	50.47	7.51	plasma membrane
TaNRT2-U.1	TraesCSU02G002800	chrUn:2667931..2669478	1548	515	55.34	9.19	plasma membrane
TaNRT2-U.2	TraesCSU02G657200LC	chrUn:466241336..466242124	780	259	28.00	8.15	endoplasmic reticulum

### Phylogenetic analysis of *TaNRT2s*

**Fig. 2:**
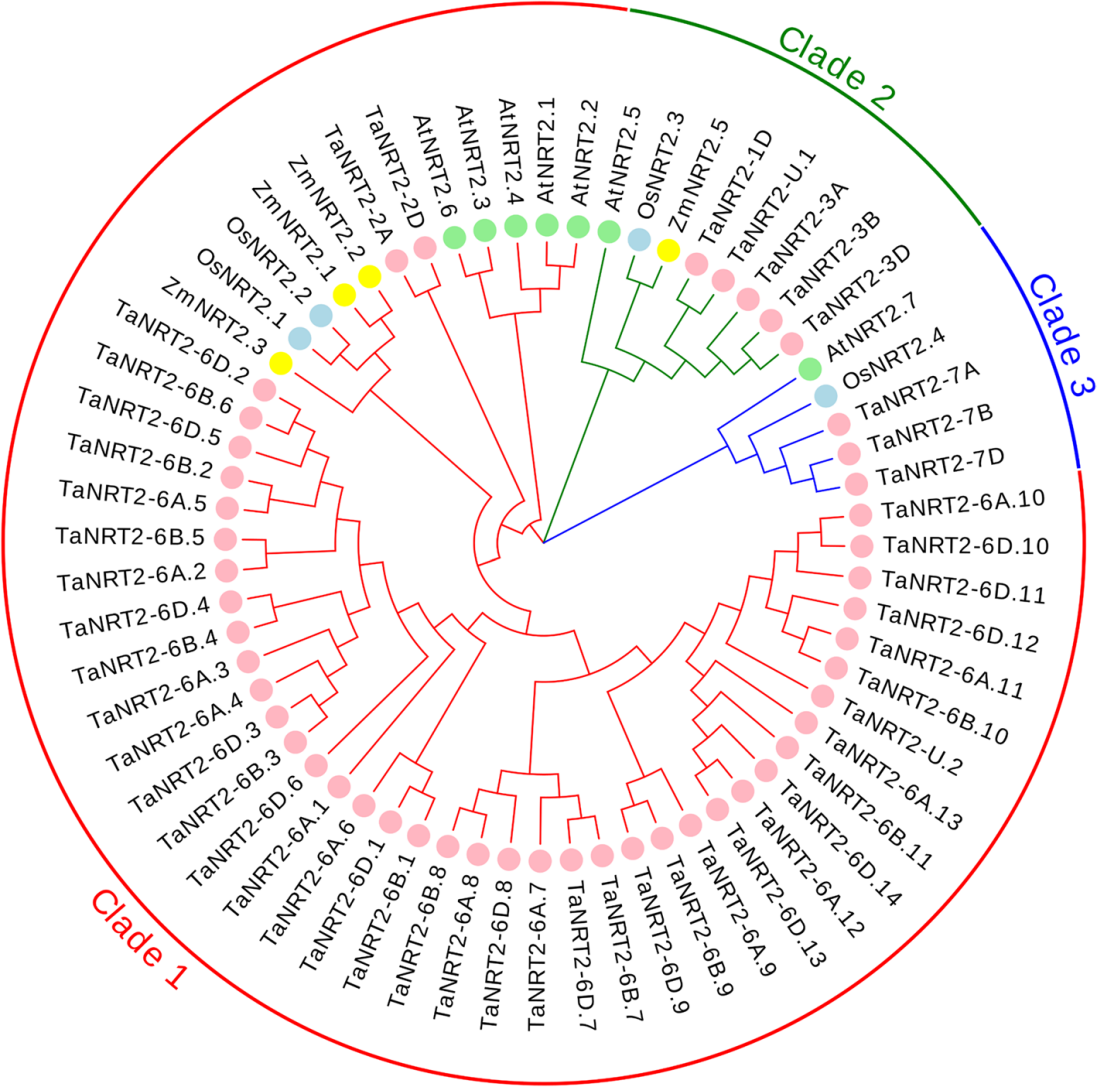
Phylogenetic tree of the *NRT2* genes family. Phylogenetic tree of NRT2 members in higher plants was generated by protein sequence alignment with MEGA 6.0 using the neighbor-joining method, displayed by Evolview 2.0. All NRT2 proteins were assigned into three groups as clade1, 2 and 3 (indicated by red, green and blue, respectively). At: *A. thaliana*, Os: *O. sativa*, Zm: *Z. mays*, Ta: *T. aestivum* (marked by a light green, blue, yellow and pink circle, respectively)

To investigate the phylogenetic relationship between *TaNRT2*s and *NRT2s* from other plant species, a neighbor-joining phylogenetic tree consisting of 49 *TaNRT2*s, 4 *OsNRT2*s, 4 *ZmNRT2*s, and 7 *AtNRT*2s was generated after multi-alignment of the protein sequences (Fig. 2). The total number of *NRT2* genes in wheat is far larger than rice, maize and Arabidopsis, which is partly a result of the hexaploidy nature of wheat. However, even when corrected for ploidy level, the number of *NRT2* genes in the wheat ABD sub-genome was significantly larger than that in rice and Arabidopsis (Fig. S1a). The ratio of total *NRT2* genes between wheat and rice or wheat and Arabidopsis was significantly higher than the expected 3:1 ratio (Fig. S1b). This indicated that the expansion of *NRT2* genes in wheat was not only due to hexaploidy but also due to a large number of tandem duplications during the evolution of wheat. According to the phylogenetic tree, the *NRT2*s were clustered into three main clades and each clade contained monocots and dicots. Clade 1 contained the most members, including 41 *TaNRT2*s, 5 *AtNRT2*s, 3 *ZmNRT2*s and 2 *OsNRT2*s. Of the 41 *TaNRT2*s in clade 1, 36 were located on chromosome 6 and divided into two branches, suggesting that two gene duplication events occurred on chromosome 6 during the formation and evolution of the wheat *NRT2* gene. The five *TaNRT2*s in clade 2 and the three *TaNRT2*s in clade 3 were homologous to *AtNRT2.5* and *AtNRT2.7*, respectively. These results suggest that duplications and multiplications have contributed to the expansion of the *TaNRT2* gene family in wheat.

### Conserved domain and gene structure analysis

**Fig. 3:**
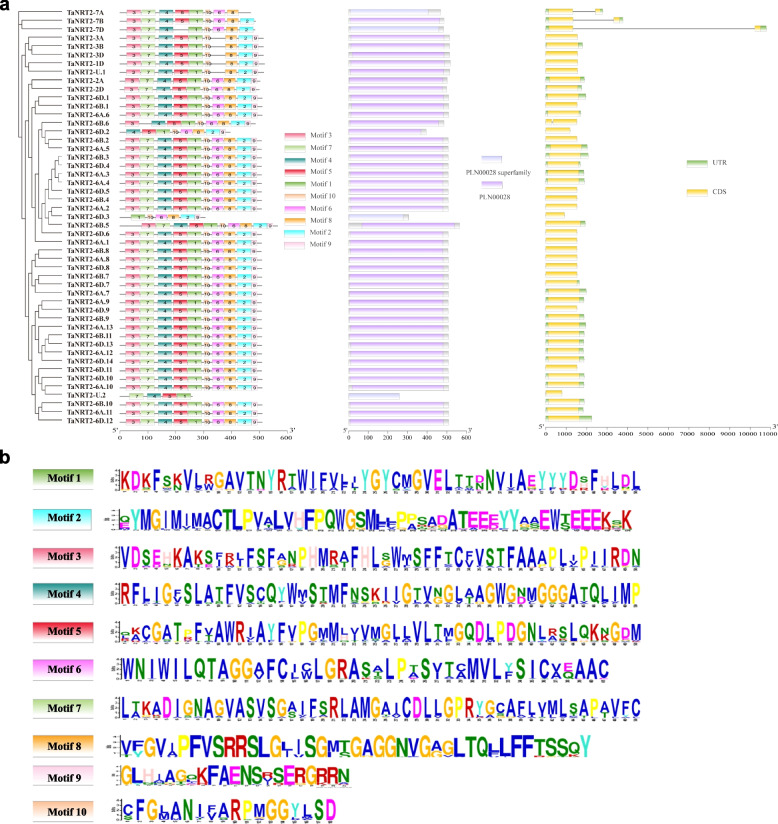
The gene structure analysis and conserved motifs of *TaNRT2* genes. **a** Conserved motifs, conserved domain and gene structures of *TaNRT2* genes. MEME motif: Ten MEME motifs are colored by different color. The length of each box in the figure does not represent the actual motif size. NCBI CDD: The conserved domains are represented by purple boxes. Gene structure: exons, introns, and untranslated regions (UTRs) are indicated by yellow rectangles, gray lines, and green rectangles, respectively. **b** Sequence logo conserved motif of *TaNRT2* proteins. The overall height of each stack represents the degree of conservation at this position, while the height of individual letters within each stack indicates the relative frequency of the corresponding amino acids

The conserved protein motifs, conserved domain, and gene structure were characterized to further understand the evolutionary characteristics of the *TaNRT2* gene family. Ten motifs were identified using MEME to illustrate the protein structure of the TaNRT2 family (Fig. 3a and b). The results showed that 37 TaNRT2s contained all the motifs, seven TaNRT2s contained nine motifs and three TaNRT2s contained eight motifs. Only motif 1 was present in all 49 TaNRT2 proteins. TaNRT2-U.2 contained the fewest number of motifs because it contained only motifs 1, 4, 5, and 7. The *TaNRT2* gene family was identified by the presence of a nitrate transmembrane transporter domain (Pfam PLN00028).

The exon–intron structures were analysed to further understand the structural characteristics of the *TaNRT2* genes (Fig. 3a). The results showed that most *TaNRT2* genes had similar gene structures. There were 1–2 exons in the *TaNRT2* genes. Among the 49 *TaNRT2* genes, four genes (*TaNRT2-7A, TaNRT2-7B, TaNRT2-7D,* and *TaNRT2-6B.6*) contained one intron, while the other 45 *TaNRT2* genes had no intron. These results suggested that the similar features of wheat *NRT2* genes might be due to duplication events during species evolution.

### Prediction of *cis*‑regulatory elements (CARE) in *TaNRT2s*

**Fig. 4:**
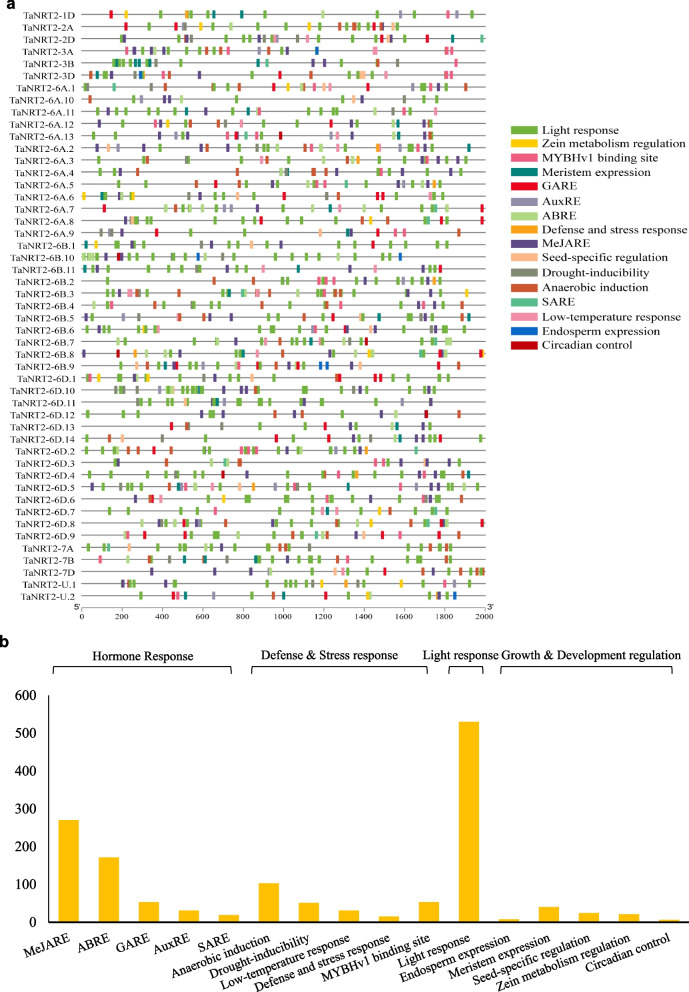
*Cis*-acting regulatory elements (CAREs) of the *TaNRT2* gene family. **a** The CAREs analysis was performed with a 2 kb upstream region using PlantCARE online server. **b** The distribution of CAREs in the promoter of *TaNRT2* genes. Most commonly occurring CAREs in *TaNRT2s*

*Cis*-regulatory elements play a role in the transcriptional regulation of various biological processes, including phytohormone responses, defence responses, and developmental processes. To further understand the potential regulatory mechanism controlling *TaNRT2* genes, the PlantCARE database (https://bioinformatics.psb.ugent.be/webtools/plantcare/html/) was used to identify putative *cis*-acting elements in the 2000 bp promoter region of *TaNRT2*s [35]. A total of 16 CAREs were identified in the 49 *TaNRT2* genes, including hormone responses, defence and stress-responsive, light response, growth, and development regulation (Fig. 4a). The CAREs involved in light, MeJA, abscisic acid response, and anaerobic induction were the most abundant in the *TaNRT2* gene family (Fig. 4b). These results suggest that *TaNRT2* family members may play roles in diverse developmental processes, such as phytohormones, stress responses, and light responses.

### Expression profiling of *TaNRT2s* in various tissues

**Fig. 5:**
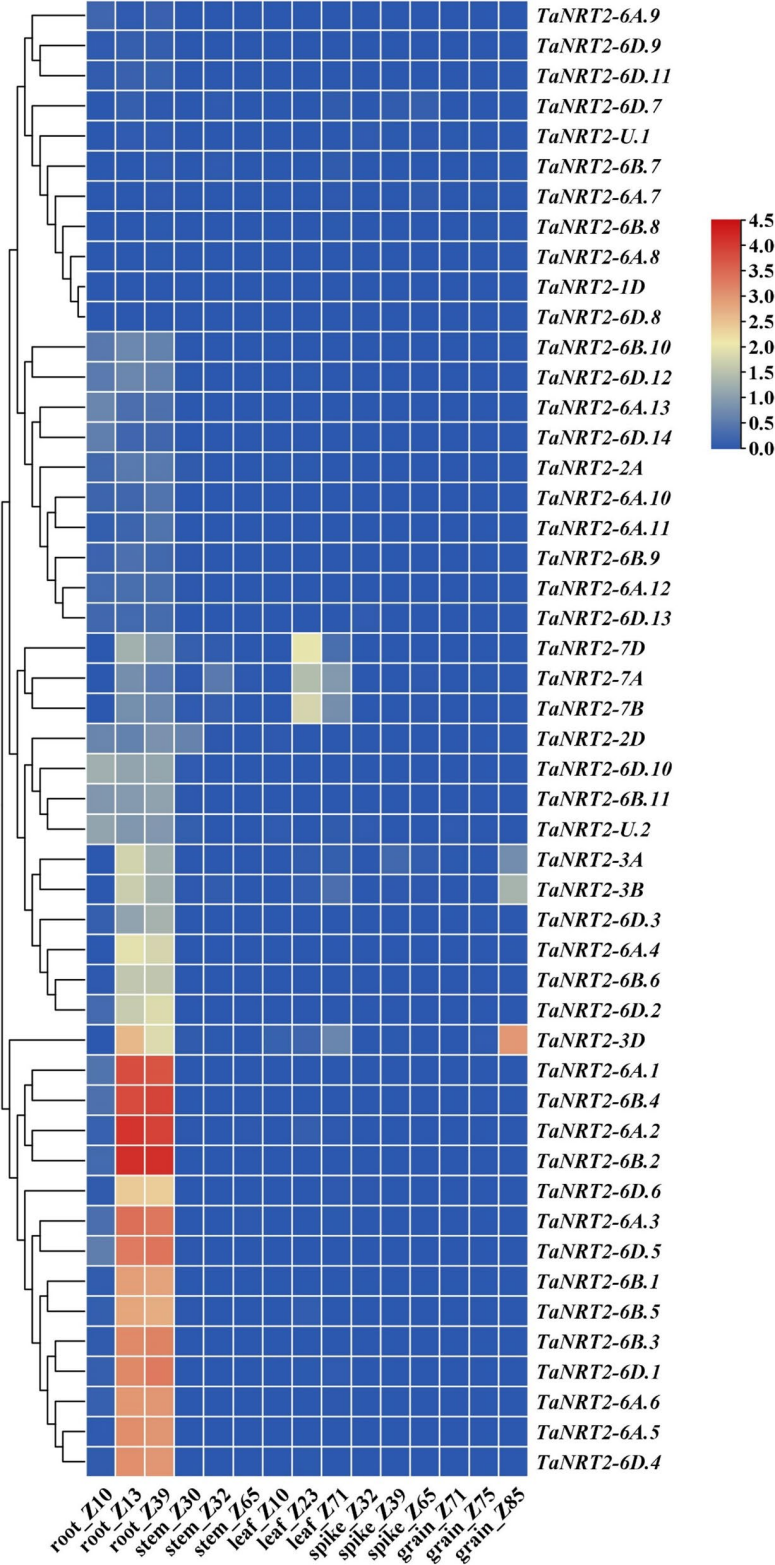
Heatmap representing the expression pattern of *TaNRT2* genes in various developmental stages. The TPM values normalized by logarithmic scale were used to construct the heatmap. Z10 ~ Z85 represent different growth stage of wheat. Different colors represent relative expression levels, as shown in the legend on the right. The horizontal axis represents the names and classifications genes, and the vertical axis represents various tissues. The rows of the heat map are clustered according to the expression patterns

The publicly accessible RNA seq database for hexaploidy common wheat (var. Chinese spring), which includes various tissues and stages, was explored in order to analyse the expression profiles of *TaNRT2* genes in different tissues, such as roots, stems, leaves, spikes, and grains. The transcripts per million (TPM) values for the *TaNRT2* genes were used to construct a heatmap (Fig. 5). The heatmap indicated that most *TaNRT2* genes were specifically expressed in the roots, especially in the roots during the Z13 and Z39 stages. However, three genes (*TaNRT2-3A, TaNRT2-3B*, and *TaNRT2-3D*) also showed high expression levels in Z85 grains, which suggested that these three genes may participate in nitrogen accumulation in grains. Furthermore, *TaNRT2-7A*, *TaNRT2-7B*, and *TaNRT2-7D* were highly expressed in the leaves, which indicated that these genes may play important roles in nitrate distribution. The expression pattern for *TaNRT2* genes suggested that the wheat *NRT2* family members can be divided into two groups. The genes in group I showed low expression or were not expressed in the roots and other tissues. However, the group II genes showed highly specific expression in the roots. These results showed that these genes play important roles in NO_3_^−^ absorption and transportation under N-limiting conditions.

### RNA-seq analysis of nitrogen deficiency

**Fig. 6:**
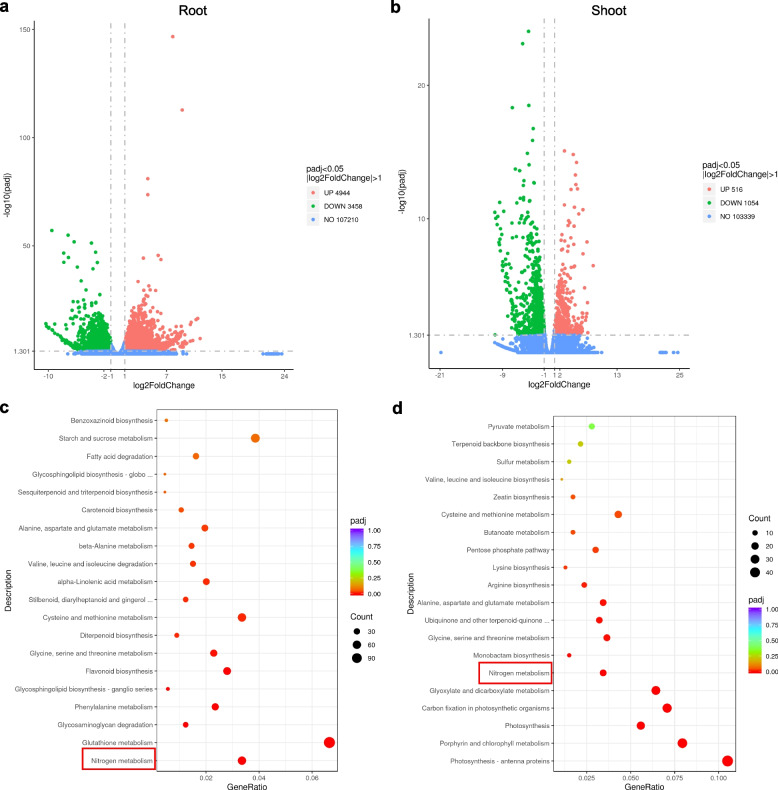
KEGG enrichment scatter plot of root and volcano plot under nitrogen deficiency treatment. The volcano indicating the DEGs in roots (**a**) and shoots (**b**), each dot in the figure represents a particular gene, and the red dots indicate significantly up-regulated genes, the green dots indicate significantly down-regulated genes, and the blue dots represent non-significant differential genes. The 20 most significantly DEG-enriched pathways of wheat seedling in roots (**c**) and shoots (**d**) under nitrate deficiency treatment

To explore whether *TaNRT2* genes are induced by nitrogen deficiency, the widely cultivated variety ‘Chuanmai104’ was used for the nitrogen deficiency treatment. When grown to the two-leaf stage, one group of seedlings was subjected to three days of low nitrogen (0 mM nitrate) and seedlings grown in normal nutrient solution (5 mM nitrate) were used as controls. After treatment, the seedlings were divided into roots and shoots. Three biological replicates and a total of 12 samples were used for the RNA-seq analysis. The differentially expressed gene (DEG) analysis indicated that there were 4944 significantly upregulated genes and 3458 significantly downregulated genes in the roots after low nitrogen treatment (Fig. 6a). However, the number of DEGs substantially decreased in the shoots. There were only 516 significantly upregulated genes and 1054 significantly downregulated genes in the low-nitrogen treated shoots (Fig. 6b). This indicated that there are more genes responding to nitrogen signals in the roots.

GO enrichment analysis revealed that carbohydrate and polysaccharide metabolic processes were enriched in the roots, while photosynthesis and oxidoreductase activity were enriched in the shoots (Fig. S2). KEGG analysis showed that nitrogen metabolism and glutathione metabolism were the two most significantly altered pathways in the roots after the plants had been subjected to the nitrogen deficiency treatment (Fig. 6c), while photosynthesis-related pathways and nitrogen metabolism were significantly enriched in the shoots (Fig. 6d). These results suggest that genes in the roots and shoots respond to stress caused by nitrogen deficiency.

**Fig. 7:**
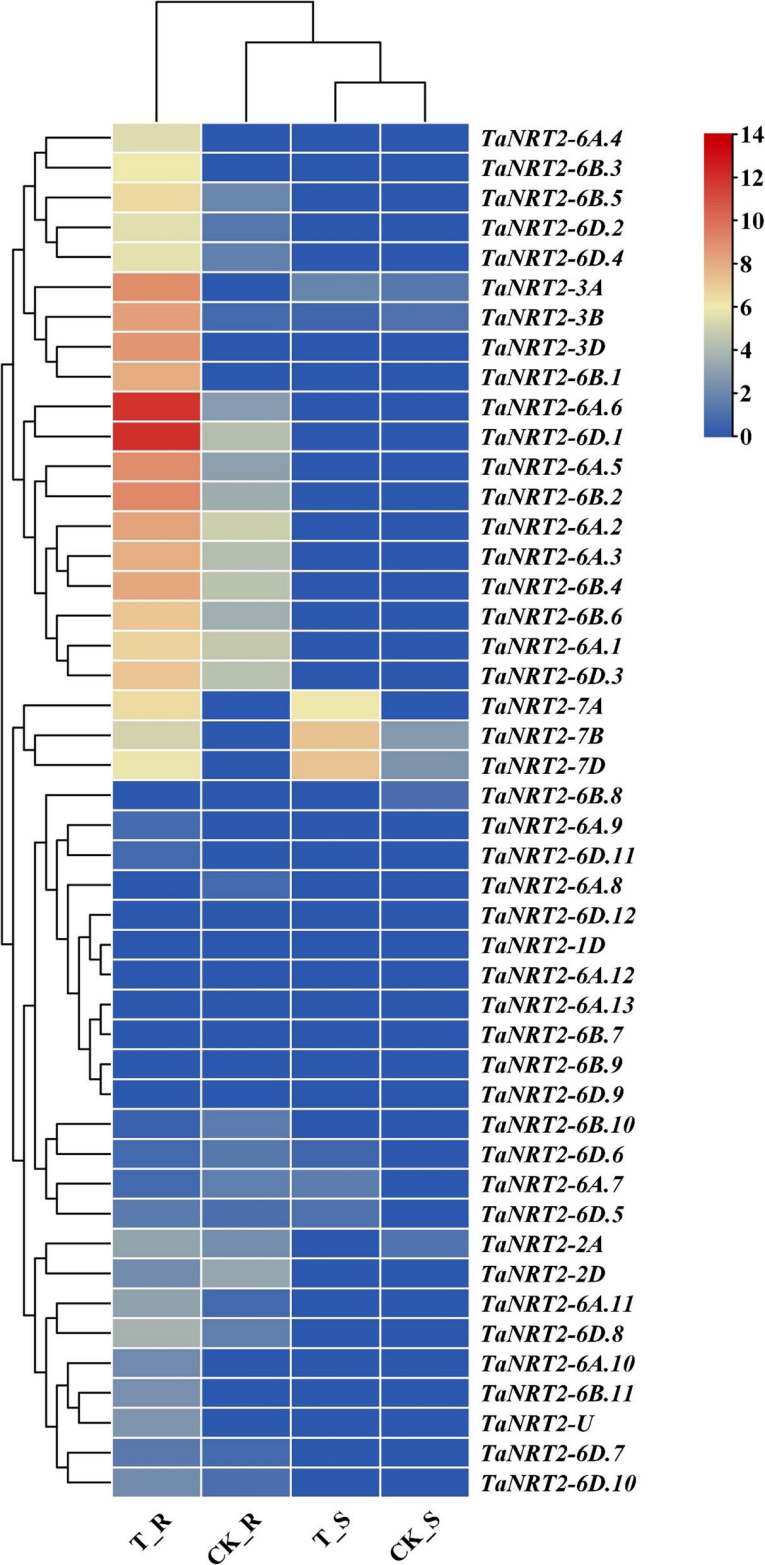
Heatmap representing the expression of *TaNRT2* genes under nitrogen deficiency treatment. The FPKM values of all *TaNRT2s* from transcriptome databases were used to construct the heatmap. The color represent relative expression levels. T_R: low nitrogen treated roots, CK_R: control roots, T_S: low nitrogen treated shoot, CK_S: control shoots

To investigate the expression of the *TaNRT2* genes, the FPKM values of all the *TaNRT2* genes identified by RNA-seq were used to construct a heatmap (Fig. 7). The results showed that the expression of 24 *TaNRT2* genes were induced by nitrogen deficiency. Among them, *TaNRT2-6D.1* and *TaNRT2-6A.6* were most significantly induced by nitrogen deficiency in the roots, indicating that they may play important roles in nitrogen absorption and metabolism. In addition, the expression of *TaNRT2-7A/B/D* were upregulated in both the roots and shoots after the nitrogen deficiency treatment. This indicates that *TaNRT2-7A/B/D* may also participate in the nitrogen transfer and accumulation in the shoots in addition to nitrogen uptake in the roots. The expression of the remaining 25 *TaNRT2* genes did not change under nitrogen deficiency condition, indicating that the genes in the *TaNRT2* family might have functionally differentiated or been made functionally redundant during evolution.

### Phenotypic and expression analyses of two different wheat varieties

**Fig. 8:**
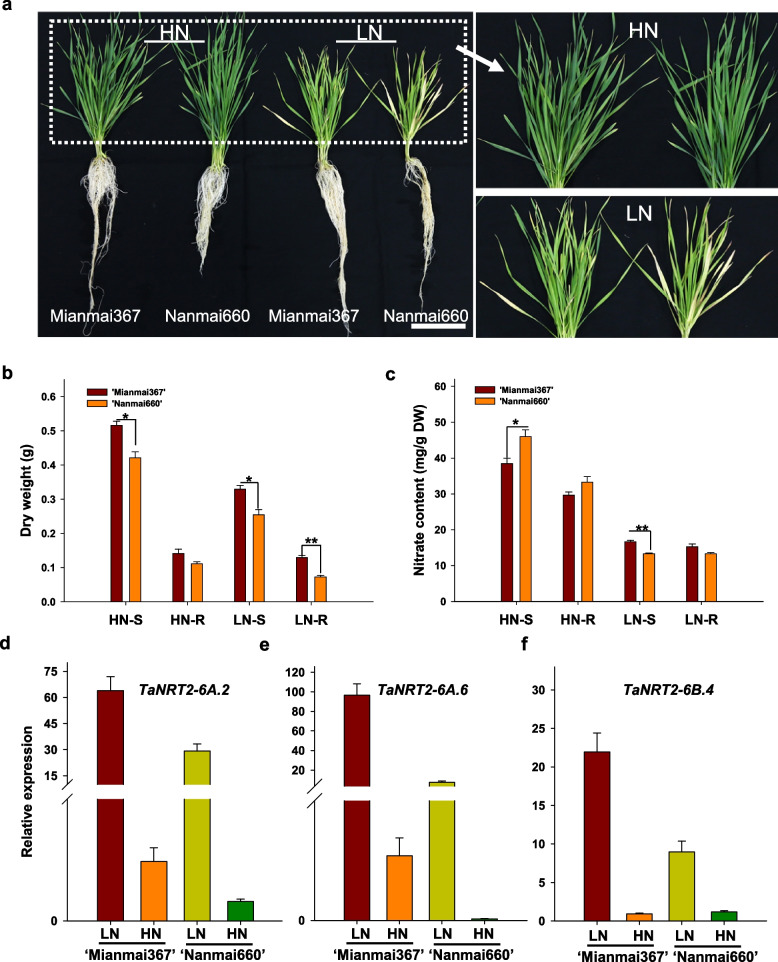
Phenotype comparison between two wheat cultivars under low nitrogen condition. **a** Phenotype of ‘Mianmai367’ and ‘Nanmai660’ under HN (5 mM NO_3_^−^) and LN (0.1 mM NO_3_^−^) conditions for 12 days; **b** Dry weight of shoot and root under HN and LN condition; **c** Nitrogen contents of shoot and root under HN and LN condition. White scale bar represents 10 cm. Data are means ± SEM (*n* = 4). Differences between mean values of treatments and controls were compared using t—tests (* *P* < 0.05, ** *P* < 0.01). S: shoot; R: root; **d-f** Relative expression of three *NRT2* genes in root of two different wheat cultivars under phenotype condition

Two different NUE wheat varieties, ‘Mianmai367’ and ‘Nanmai660’, were used to investigate the phenotypes under nitrate-limited conditions. The two-leaf stage seedlings were transferred to high nitrate (5 mM, HN) and low nitrate (0.1 mM, LN) Hoagland hydroponic solutions as the control and treatment conditions, respectively. After treatment for 12 days, the nitrate-deficient phenotypes of the two varieties were evaluated and the dry weight and nitrogen content were measured. The cultivar ‘Mianmai367’ showed obvious tolerance to low nitrate stress compared to ‘Nanmai660’ (Fig. 8a). The biomass results were consistent with the observed phenotypes. The root and shoot biomasses for ‘Nanmai660’ were obviously lower than those for ‘Mianmai367’ (Fig. 8b). The nitrogen content in ‘Nanmai660’ was also lower than in ‘Mianmai367’ (Fig. 8c). The results showed that there was a significant difference in LN tolerance between the two wheat genotypes, further indicating that the nitrogen nutritional activities of wheat are genetically regulated through gene expression that increases its efficient use or tolerance to low nitrogen.

To further understand the NUE differences between the two wheat varieties, we further investigated several highly expressed genes (*TaNRT2-6A.2*, *TaNRT2-6A.6*, and *TaNRT2-6B.4*) under low nitrate conditions. The results showed that the expression of the three genes were all upregulated under N-limiting conditions in the two varieties. However, the three genes had higher expression levels in ‘Mianmai367’ than in ‘Nanmai660’ (Fig. 8d-f). These results showed that ‘Mianmai367’ had a greater ability to efficiently utilize N than ‘Nanmai660’.

## Discussion

In the past two decades, the *NRT2* gene family has been identified in numerous plant genomes, such as Arabidopsis [10, 16], rice [17], maize [18], rapeseed [19], poplar [21] and tomato [22], and the number of *NRT2* genes ranges from 4 (rice, maize and tomato) to 31 (rapeseed). In this study, a genome-wide analysis revealed 49 *NRT2* members in wheat and identified three new genes compared to a previous report [36]. The numbers of wheat *NRT2* genes were significantly more than those of rice, Arabidopsis and other species at both the genome and sub-genome level (Fig. S1). According to the evolutionary relationship, these genes can be divided into three clades, which is consistent with reports in other species. In addition, all the *NRT2* genes had a conserved MFS domain and multiple transmembrane domains (Table 1).

A total of 38 out of the 49 *TaNRT2* genes were located on chromosome 6 (Fig. 1), and there were three tandem repeat gene pairs on chromosome 6A, 6B and 6D. These genes also had good collinearity between ABD sub-genomes and a close evolutionary relationship (Fig. 2). It is speculated that whole genome duplication and tandem duplication might contribute to *NRT2* gene expansion in wheat. It has been reported that 16 gene duplication events occurred during the evolution of the wheat *NRT2* gene family [37]. All *TaNRT2* genes on chromosome 2 and 6 were classified into clade 1, since an ancient *NRT2* gene duplicated into two copies on chromosomes 2 and 6 after the monocot-dicot split [37].

Many studies have indicated that the exon–intron patterns are commonly conserved in gene families or subfamilies in plants. In this study, we analysed the gene location, gene structure, conserved motifs, *cis*-acting regulatory elements, and gene expression profiles of all the *TaNRT2* genes (Figs. 3, 4 and 5). The gene structure analysis showed that all the *TaNRT2*s had one exon, except for the three genes on chromosome 7 and one gene on chromosome 6 which had two exons (Fig. 3a). The intron length of genes on chromosome 7 (*TaNRT2-7A/7B/7D*), especially *TaNRT2-7D*, was even longer than the coding region*.* Furthermore, a previous study revealed that *TaNRT2-7A/7B/7D* had experienced a third duplication during the evolution of the wheat *NRT2* gene family [37]. This suggests that an extra exon acquisition might have occurred in *TaNRT2-7A/7B/7D* during wheat evolution and that this has led to the various structures seen in this family.

**Fig. 9:**
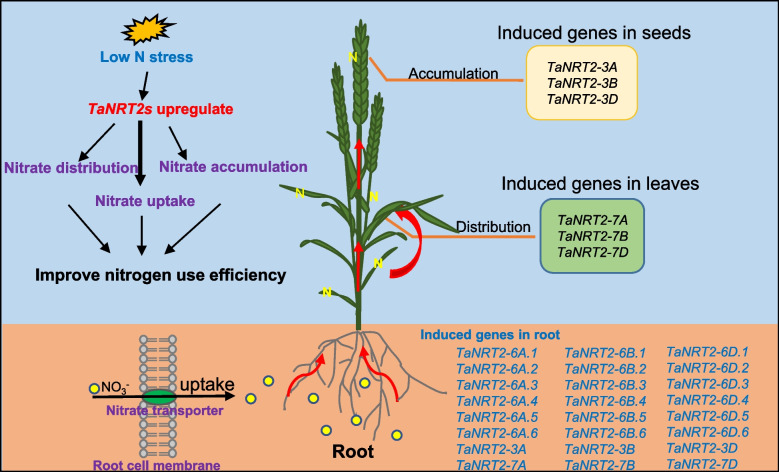
Model of expression patterns and induced *TaNRT2* genes in wheat under nitrogen deficiency condition. The induced *TaNRT2* genes were summarized in the model which based on the transcript data and the heatmap of *TaNRT2* genes in different developmental stage. Under nitrate deficiency condition, most *TaNRT2* genes are induced in root, these high-affinity nitrate transporters uptake NO_3_^−^ by trans-membrane. Then the nitrate were distributed in leaves and seeds to assimilation. The red arrows indicate nitrate uptake and transport. The green ellipse on the membrane characterizes TaNRT2 proteins and the little yellow ellipses response nitrate molecules

The *NRT2* family is involved in the high-affinity nitrate transporter systems and plays vital roles in both nitrate uptake and translocation in plants. We conducted a transcriptome analysis of bread wheat under nitrate deficiency conditions to further understand the *TaNRT2* transcript level changes and function of *TaNRT2s* (Figs. 6, 7 and 8). Transcriptome analysis showed that the genes involved in nitrogen metabolism were observably changed under nitrate-limited conditions (Fig. 6) and approximately half of the wheat *NRT2* genes were upregulated in roots under nitrate stress (Fig. 7). We selected two different NUE wheat varieties and identified the expression levels of three key *TaNRT2* genes (*TaNRT2-6A.2*, *TaNRT2-6A.6*, *TaNRT2-6B.4*) in the two different NUE wheat varieties (Fig. 8). These three genes were highly expressed in nitrogen efficient material. Furthermore, we identified the function of the three genes in nitrate uptake which marked with ^15^N in *Xenopus* oocytes. The results showed that compared with water controls, single injection of *TaNRT2-6B.4* observably increased in ^15^N accumulation, while *TaNRT2-6A.2* and *TaNRT2-6A.6* were indistinguishable with control (Fig. S3). It was reported that NRT2 proteins need form complexes with NRT3 to target the plasma membrane and maintain protein stability [38], and we believe there exist the same mechanism in wheat. In additional, several *TaNRT2* genes (*TaNRT2-7A/7B/7D*) were observably induced in shoots, which suggests that these genes may function in nitrate distribution, and several *TaNRT2* genes (*TaNRT2-3A/3B/3D*) were observably induced in seeds which suggests that these genes may participate in nitrogen accumulation in grains (Fig. 9). Interestingly, almost half of the *TaNRT2* genes showed low expression or were not expressed in the roots and other tissues with or without nitrate deficiency treatment (Figs. 5 and 7). We speculated that these genes may be differentiated due to the functional redundancy of the large *NRT2* gene family in wheat.

NODULE INCEPTION (NIN) is functionally necessary for nodule formation in the legume *Lotus japonicas*, and its homologous gene is known as *NINLIKE PROTEIN* (*NLP*) [39]. Recent studies have highlighted the emerging roles of NLPs in N signalling and assimilation, root cap release [40–42], and regulation of nitrate uptake/transport under low- and high-nitrate conditions by combining to NRT2s [43, 44]. In this study, we analysed the gene expression profiles of 18 *TaNLP* genes (Fig. S4). Interestingly, we found that several *TaNLP* genes were highly expressed in seeds and roots. To further analyse the relationship between TaNRT2s and TaNLPs, we cloned several *TaNRT2s* and *TaNLPs* which highly expressed in roots (*TaNRT2-6A.6*,* TaNRT2-3D*, *TaNLP7*) and seeds (*TaNRT2-3D*, *TaNLP1*, *TaNLP3*, *TaNLP4*) for yeast one-hybrid assay. The results showed that they can interact, suggesting that TaNLPs may interact with TaNRT2s to maintain nitrogen homeostasis in wheat (Fig. S5). The finding may provide a basis for future studies concerning the roles of the *TaNRT2s* and *TaNLPs* in wheat.

Post-translational modification (PTM) plays a key role in cellular biological functions and it has been reported that protein phosphorylation represents 53.5% of all PTMs [45, 46]. The high-affinity transporter AtNRT2.1 has been shown to be rapidly de-phosphorylated after 3 min of nitrate resupply [47]. Subsequent studies have revealed that substitution of Ser28 resulted in unstable and de-phosphorylated AtNRT2.1 that failed to complement the growth‐restricted phenotype of the *nrt2.1* mutant under low nitrate supply [48]. Another key phospho-site for AtNRT2.1 activity is Ser501, which can inactivate AtNRT2.1 function when mimicking the constitutive phosphorylation of this residue in transgenic plants [49]. Based on these results, we hypothesized that the two phosphor-sites may be highly conserved. We analysed all the NRT2 proteins by amino acid sequence alignment in wheat, rice, maize, and Arabidopsis (Additional file 1). The results showed that phosphor-site Ser28 was not conserved in rice, maize, and wheat, but was conserved in Arabidopsis. Another phospho-site, Ser501, was very conserved in maize and rice, except for OsNRT2.4, whereas it was only partly conserved in wheat. It has been reported that a large-scale expansion of *NRT2* genes has occurred in *Triticeae* and is mainly concentrated on chromosome 6 [37]. In this study, 35 *TaNRT2* genes located on chromosome 6 and three *TaNRT2* genes located on chromosome 7 were not conserved for Ser501, while other *TaNRT2* genes located on chromosomes 1, 2 and 3 were conserved for Ser501. We suggest that those that did not contain a conserved Ser501 site on chromosome 6 may have originated from a single gene before large-scale expansion, and that this original gene did not contain the conserved site.

N and phosphorus (P) are the two most important mineral nutrients for plants. It has been reported that variations in the N:P supply ratio significantly affect their uptake and an increased N:P supply ratio greatly promotes the uptake of P [50, 51]. PHOSPHORUS STARVATION RESPONSE 1 (PHR1) is a key transcription factor involved in phosphate starvation signalling [52]. In recent years, many studies have demonstrated that PHR1 also plays an important role in nitrogen nutrition. In rice, PHR2-SPX4 and NLP3 activate both phosphate- and nitrate-responsive genes. This leads to the coordinated utilization of nitrogen and phosphorus [53]. In Arabidopsis, PHR1 and NIGT1 together regulate the acquisition of phosphorus and nitrogen [54]. In this study, the transcriptome data showed that a few *TaPHR1s* and other phosphate related genes notably changed under nitrate deficiency (Table S1). This implies that these genes play important roles in the response to phosphorus and nitrogen balance. However, verification of this finding requires further research.

## Conclusions

In summary, 49 *TaNRT2* genes distributed on 13 chromosomes were identified in the wheat genome. A hypothetical model of all the *TaNRT2*s involved in nitrate absorption, distribution, and accumulation is proposed and is based on the transcriptome analysis and the expression profiles of wheat throughout its growth and development stages (Fig. 9). In particular, several genes were specifically expressed in the roots, leaves and seeds and strongly induced by nitrogen deficiency stress. This analysis of the function of these genes will improve the NUE in wheat. However, further research is needed to clarify the nitrogen assimilation mechanism in wheat.

## Methods

### Plant growth conditions and low NO_3_- stress treatment

Three wheat varieties, ‘Chuanmai104’, ‘Nanmai660’ and ‘Mianmai367’ which are the cultivars of Southwest China were used in this study. They were cultured hydroponically in a growth chamber under the following conditions: relative humidity, 50–70%; 14-h light/10-h dark photoperiod; temperatures, 22 °C days, 22 °C nights.

A modified Hoagland nutrient solution was used in this study [42], with 5 mM KNO_3_ as sufficient nitrogen (HN) solution and 0.1 mM KNO_3_ or 0 mM KNO_3_ as LN solution. The solutions were changed every second day. The solution for HN conditions contained 5 mM KNO_3_, 1 mM KH_2_PO_4_, 2 mM MgSO_4_, 0.1 mM FeNaEDTA, 5 μM KI, 1 μM H_3_BO_3_, 0.15 mM MnSO_4_, 0.05 mM ZnSO_4_, 4 mM CaCl_2_, 0.19 mM CoCl_2_, 0.1 μM CuSO_4_ and 1 μM Na_2_MoO_4_, (pH = 5.8). The solution for the LN condition contained the same nutrients with the removal of KNO_3_, and the differences in potassium supply were balanced with KCl.

### Identification of the *NRT2* gene family in wheat

To identify putative *NRT2* genes in wheat, the known wheat NRT2 protein sequence (AAG01172.1), downloaded from NCBI (https://www.ncbi.nlm.nih.gov/), was queried by blastp on WheatOmics 1.0 [55] using the IWGSC RefSeq v1.1 (Chinese Spring) genome database (Additional file 2). The Pfam online server (http://pfam.xfam.org/search) was used to predict the conserved domains, and the TMHMM-2.0 online server (https://services.healthtech.dtu.dk/service.php?TMHMM-2.0) was used to predict NRT2 protein transmembrane helices. The sequences were processed by removing the non-conserved MFS_1 domain and less than 6 transmembrane helices, and after manual curation, a final set of 49 genes belonging to the NTR2 nitrate transporter family were selected. The NTR2 protein feature prediction, molecular weight and theoretical protein isoelectric point (pI) were predicted by ProtParam (https://web.expasy.org/protparam/), and the subcellular localization prediction was analysed by POST (http://psort1.hgc.jp/form.html). The chromosome location of *TaNRT2* genes were visualized by TBtools [56] based on the IWGSC RefSeq v1.1 wheat genome database. The collinearity was determined by MCScanX toolkit [57] using the wheat genomic DNA sequence and the gff3 file.

### Phylogenetic analysis of TaNRT2

The protein sequences of *A. thaliana*, *O. sativa* and *Z. mays* were obtained from a reported study [10, 16–18], and these protein loci are listed in Additional file 3. The full-length proteins of AtNRT2s, OsNRT2s, ZmNRT2s, and the newly identified TaNRT2s were aligned using ClustalW, and the phylogenetic tree was constructed based on the alignment using MEGA7 [58] by using neighbor-joining (NJ) algorithms with the following parameters: Jones-Taylor-Thornton (JTT) model, pairwise deletion and bootstrap (1,000 replicates), and visualization by Evolview 2.0 [59].

### Analysis of motifs, domain and gene structure

Protein motifs were identified by using MEME (Multiple Expectation Maximization for Motif Elication) (https://meme-suite.org/meme/tools/meme). Conserved domains were identified by NCBI CDD (https://www.ncbi.nlm.nih.gov/cdd/?term=). Gene structure information was extracted from the gff3 file for the wheat reference genome (IWGSC RefSeq v1.1). The characteristics of the *TaNRT2* family gene structure with motif composition and conserved domains were visualized by TBtools.

### *Cis*-acting regulatory element (CARE) analysis

*Cis*-acting regulatory elements (CAREs) were predicted by using the 2000 bp upstream region of wheat *NRT2* genes in a Plant CARE online server (http://bioinformatics.psb.ugent.be/webtools/plantcare/html/) and the distribution of CAREs on the gene promoter was visualized by TBtools.

### Expression profiling of all *TaNRT2* and *TaNLP* genes

The TPM values of wheat *NRT2* and *NLP* genes come from five tissues (root, stem, leaf, spike and grain) were obtained from the Wheat Expression Browser on the WheatOmics 1.0 online server [55]. All the TPM values were logarithmic and visualized as heatmaps of *TaNRT2s* and *TaNLPs* using the TBtools integrated toolkit. The FPKM values of all *TaNRT2* genes from transcriptome databases after nitrogen deficiency treatment were used to construct the heatmap using the TBtools integrated toolkit.

### Biomass and nitrogen content measurement

The two wheat seedlings of LN (0.1 mM NO_3_^−^) and HN (5 mM NO_3_^−^) treated in hydroponics were collected after 12 days. The nutrient solution was replaced every second day. The root and shoot tissues were harvested separately and dried at 80 °C for 3 days, and then the dry weights were recorded. The dried samples were powdered and subsequently digested with concentrated H_2_SO_4_ for the determination of total N using the Kjeldahl method [60].Three biological replicates were used for phenotypic tests, biomass and nitrogen content measurements. The t test (* *P* < 0.05, ** *P* < 0.01) was used to analyse the statistical significance.

### RNA-seq analysis

The seeds of ‘Chuanmai104’ were germinated and grown on vermiculite for 15 days to two-leaf stage, then seedlings were transferred to modified Hoagland hydroponic solution grown for 3 days. First, the wheat seedlings were cultured in normal solution for 3 days. Then, half of them were transferred to nitrogen starvation conditions (0 mM nitrate) as the NO_3_^−^-deficient treatment (LN), and the other half were transferred to normal solution (5 mM nitrate) as the control (HN) in hydroponics. After 3 days, the wheat seedlings of shoots and roots were collected, and three biological replicates were used for RNA-seq analysis (Novogene, China). Differential expression analysis of two conditions was performed using the DESeq2R package (1.20.0). DESeq2 provides statistical routines for determining differential expression in digital gene expression data using a model based on the negative binomial distribution. The resulting *P* values were adjusted using the Benjamini and Hochberg’s approach for controlling the false discovery rate. Genes with an adjusted *P* value ≦0.05 found by DESeq2 were defined as differentially expressed genes. The raw date of transcriptome data are shown in Additional files 4 and 5.

Gene Ontology (GO) enrichment analysis of differentially expressed genes was implemented by the cluster Profiler R package, in which gene length bias was corrected. GO terms with corrected *P* values less than 0.05 were considered significantly enriched by differentially expressed genes. KEGG is a database resource for understanding the high-level functions and utilities of biological systems [61], such as the cells, the organisms and ecosystems, from molecular-level information, especially large-scale molecular datasets generated by genome sequencing and other high-throughput experimental technologies (www.kegg.jp/kegg/kegg1.html). We used the clusterProfiler R package to test the statistical enrichment of differentially expressed genes in KEGG pathways.

### Uptake of K^15^NO_3_- in *Xenopus* oocytes

Coding sequences of *TaNRT2-6A.2*, *TaNRT2-6A.6*, and *TaNRT2-6B.4* were cloned into the expression vector *pT7TS*. After linearization of *pT7TS* plasmids with *Eco*RI, RNA was transcribed in vitro using an mRNA synthesis kit (mMESSAGE mMACHINE T7 kit; Ambion). *Xenopus laevis* oocytes were injected with 25 ng RNA and incubated for 60 h in ND96 solution (96 mM NaCl, 2 mM KCl, 1.8 mM CaCl_2_, 1 mM MgCl_2_, and 10 mM HEPES, pH 7.5).

For ^15^N uptake in oocytes, ten oocytes were selected treatment in NO_3_^−^ uptake solution (230 mM mannitol, 0.3 mM CaCl_2_, 10 mM MES with 0.25 mM K^15^NO_3_^−^) for 12 h at 18 °C and washed five times in ND96 solution. ^15^N was measured using an isotope ratio mass spectrometer (IRMS; DELTAplus XP) according to previous report [38, 62].

### Yeast one-hybrid assay

The coding sequence of *TaNLPs* was constructed into the vector pB42AD. *LacZ* was used as a reporter gene, driven by the fragments of *TaNRT2s* promoter in yeast. The pB42AD-*TaNLPs* and PB42AD plasmids were transformed with the *pTaNRT2s:lacZ* plasmids into *Saccharomyces cerevisiae* strain EGY48 using the PEG/LiAC method. The transformed strains were cultured on SD/-Trp-Ura plates and confirmed by PCR. Then, these transformants were grown on proper SD/-Trp-Ura plates containing X-α-gal (5-bromo-4-chloro-3-indolyl-α-D-galactopyranoside), 2% galactose, and 1% raffinose for blue colour development [63].

### RNA isolation and real-time PCR analysis

The shoots and roots of wheat seedlings were treated with nitrate limited conditions (0.1 mM nitrate) and complete nutrient solution (5 mM nitrate) for 12 days, and these seedlings were collected and immediately frozen in liquid nitrogen and stored at − 80 °C. The total RNA of wheat seedlings was extracted with RNA extraction kit (EASYspin Plus Complex Plant RNA Kit) [64], and treated with DNase I (Takara, Dalian, China) to eliminate genomic DNA contamination. Then, the total RNA was used to synthesize cDNA with a reverse transcription reaction kit (Thermo Scientific, Lithuania). The qRT‒PCR assay was conducted as described previously [65]. Amplification of wheat *α-tubulin* gene was used as an internal control to normalize the data. The primers used are listed in Table S2. The gene-specific primers were designed using NCBI (Primer designing tool (nih.gov)) and DNAMAN software.

## Supplementary Information

**Additional file 1.** A multiple sequence alignment of all the TaNRT2 proteins.

**Additional file 2.** The *NRT2* genes information in wheat.

**Additional file 3.** The amino acid sequence of NRT2s in *Arabidopsis*, maize, rice and wheat.

**Additional file 4.** The raw data of transcriptome in root under nitrogen deficiency condition.

**Additional file 5.** The raw data of transcriptome in shoot under nitrogen deficiency condition.

**Additional file 6: Fig. S1.** The number and ratio of NRT2 genes in wheat, rice, maize and Arabidopsis. a The number of NRT2 genes in wheat genome and sub-genome, rice, maize and Arabidopsis. b The ratio of total NRT2 gene is shown for wheat : rice (red) and wheat : Arabidopsis (orange). The expected ratio (3 : 1) is indicated by a black dotted line. **Fig. S2.** Gene classification was based on GO analysis for DEGs under nitrate deficiency condition. The numbers of DEGs in each GO term was significantly enriched in root (a) and shoot (b). Functional categorization of genes based on the biological process of gene ontology. Different classes are shown for BP (biological process) ,CC (cellular component) and MF (molecular function). The y‑axis shows the counts of differently expressed genes, and the x‑axis shows GO term of gene enriched in each biological process. **Fig. S3.** K^15^NO_3_ uptake into *Xenopus* oocytes. oocytes injected with water as control, cRNA of *TaNRT2-6A.2*, *TaNRT2-6A.6*, *TaNRT2-6B.4* were injected alone, respectively.^15^N enrichment per oocyte is expressed as delta ^15^N compared with standard atmospheric ^15^N : ^14^N ratio. Values are average of *n* = 6 ± SD. Differences between mean values of treatments and controls were compared using t - tests (* *P*< 0.05). **Fig. S4.** Heatmap representing the expression pattern of *TaNLP* genes in various developmental stages. The TPM values normalized by logarithmic scale were used to construct the heatmap. Z10~Z85 represent different growth stage of wheat. Different colors represent relative expression levels, as shown in the legend on the right. The horizontal axis represents the names and classifications genes, and the vertical axis represents various tissues. The rows of the heat map are clustered according to the expression patterns. **Fig. S5.** Yeast one-hybrid (Y1H) assay was used to verify TaNLPs bound to the *TaNRT2s* promoter region. TaNLPs fusion proteins activate the expression of *LacZ* reporter gene driven by the promoter of *TaNRT2-6A.6* and *TaNRT2-3D*, respectively, in yeast. The empty vector pB42AD was used as a negative control. **Table S1.** Phosphorus signaling pathway genes respond to low nitrogen stress. **Table S2.** Primers used in this study.

## Data Availability

All data generated or analyzed during this study are included in this article and additional files. The transcriptome sequence were generated in Novogene company (https://cn.novogene.com/). The datasets generated during the current study are available in the National Center for Biotechnology Information Sequence Read Archive (SRA) under accession number PRJNA925925 (https://www.ncbi.nlm.nih.gov/Traces/study/?acc=PRJNA925925&o=acc_s%3Aa). The datasets supporting the conclusions of this article are included within the article (and its additional files).

## Abbreviations

DEG: Differently expressed gene
RT-PCR: Real-time polymerase chain reaction
NUE: Nitrogen utilization efficiency
NRT: Nitrate transporter
MFS: The major facilitator superfamily
LN: Low nitrogen
HN: High nitrogen
LATSs: Low-affinity nitrate transporter systems
HATSs: High-affinity nitrate transporter systems
GO: Gene ontology
KEGG: Kyoto Encyclopedia of Genes and Genomes
FPKM: Fragments per kilobase of exon per million fragments mapped
TPM: Transcripts per million
PTM: Post-translational modification
