# Supplementary material for: Genome-wide systematic characterization of the NRT2 gene family and its expression profile in wheat (Triticum aestivum L.) during plant growth and in response to nitrate deficiency
Source: BMC Plant Biol. 2023 Jul 7;23:353. doi: 10.1186/s12870-023-04333-5 (PMC10327373; doi:10.1186/s12870-023-04333-5)
Supplement: Supplementary file 1 — Additional file 1. A multiple sequence alignment of all the TaNRT2 proteins. [file 12870_2023_4333_MOESM1_ESM.docx]

Additional file 1. A multiple sequence alignment of all the TaNRT2 proteins.

OsNRT2.4---------------------------------------------------MVAMEKKTKLVEEEDGCYY 19

TaNRT2-7A--------------------------------------------------MVTMGKKV----DQEQSYY 15

TaNRT2-7B--------------------------------------------------MVTMGKKV----DQEQSYY 15

TaNRT2-7D--------------------------------------------------MVTMGKKV----DQEQSYY 15

AtNRT2.7---------------------------------------------------------------------- 0

AtNRT2.5---------------------------------------------------------------------- 0

OsNRT2.3--------------------------------------------------------------MEA-KPVA 7

ZmNRT2.5-----------------------------------------------------------MAEGEF-KPAA 10

TaNRT2-3A---------------------------------------------------------MEG--ES-KPAA 9

TaNRT2-3B---------------------------------------------------------MEG--ES-KPAA 9

TaNRT2-3D---------------------------------------------------------MEG--ES-KPAA 9

TaNRT2-1D---------------------------------------------------------MEGASNW-GPAA 11

TaNRT2-U.1--------------------------------------------------------MEGA----AAME 8

TaNRT2-2A--------------------------------------------------------------------- 0

TaNRT2-2D--------------------------------------------------------------------- 0

ZmNRT2.3------------------------------------------------------------------MASD 4

TaNRT2-6B.7-----------------------------------------------------------------ME 2

TaNRT2-6A.7-----------------------------------------------------------------ME 2

TaNRT2-6D.7-----------------------------------------------------------------ME 2

TaNRT2-6D.8-----------------------------------------------------------------ME 2

TaNRT2-6A.8-----------------------------------------------------------------ME 2

TaNRT2-6B.8------------------------------------------------------------------- 0

TaNRT2-6A.9-----------------------------------------------------------------ME 2

TaNRT2-6B.9-----------------------------------------------------------------ME 2

TaNRT2-6D.9-----------------------------------------------------------------ME 2

TaNRT2-6A.13----------------------------------------------------------------ME 2

TaNRT2-6B.11----------------------------------------------------------------ME 2

TaNRT2-6D.14----------------------------------------------------------------ME 2

TaNRT2-6A.12----------------------------------------------------------------ME 2

TaNRT2-6D.13----------------------------------------------------------------ME 2

TaNRT2-6D.11------------------------------------------------------------------ 0

TaNRT2-6D.12----------------------------------------------------------------ME 2

TaNRT2-6B.10----------------------------------------------------------------ME 2

TaNRT2-6A.11----------------------------------------------------------------ME 2

TaNRT2-6D.10----------------------------------------------------------------ME 2

TaNRT2-6A.10----------------------------------------------------------------ME 2

TaNRT2-U.2-------------------------------------------------------------------- 0

TaNRT2-6D.1-----------------------------------------------------------------ME 2

TaNRT2-6A.6-----------------------------------------------------------------ME 2

TaNRT2-6B.1-----------------------------------------------------------------ME 2

TaNRT2-6B.6-----------------------------------------------------------------ME 2

TaNRT2-6B.4-----------------------------------------------------------------ME 2

TaNRT2-6D.5------------------------------------------------------------------- 0

TaNRT2-6A.2-----------------------------------------------------------------ME 2

TaNRT2-6B.5-------MSTPALYPTINTQSIQDPKHQTTQPLATPSCRPLQVAKLSSKELAEKKPSSVRTGEADME 60

TaNRT2-6B.3-----------------------------------------------------------------ME 2

TaNRT2-6A.1-----------------------------------------------------------------ME 2

TaNRT2-6A.3-----------------------------------------------------------------ME 2

TaNRT2-6D.3-----------------------------------------------------------------ME 2

TaNRT2-6D.6-----------------------------------------------------------------ME 2

TaNRT2-6D.4-----------------------------------------------------------------ME 2

TaNRT2-6B.2-----------------------------------------------------------------ME 2

TaNRT2-6A.5-----------------------------------------------------------------ME 2

TaNRT2-6A.4-----------------------------------------------------------------ME 2

TaNRT2-6D.2------------------------------------------------------------------- 0

OsNRT2.1-------------------------------------------MDSSTV---GAPGSSLHGVTGREPAFA 24

OsNRT2.2-------------------------------------------MDSSTV---GAPGSSLHGVTGREPAFA 24

ZmNRT2.1---------------------------------------------MAAV---GAPGSSLHGVTGREPAFA 22

ZmNRT2.2---------------------------------------------MAAV---GAPGSSLHGVTGREPAFA 22

AtNRT2.6-------------------------------------------MAHNHSNEDGSIGTSLHGVTAREQVFS 27

AtNRT2.3-------------------------------------------MTHNHSNEEGSIGTSLHGVTAREQVFS 27

AtNRT2.4-----------------------------------------------MADGFGEPGSSMHGVTGREQSYA 23

AtNRT2.1-----------------------------------------------MGDSTGEPGSSMHGVTGREQSFA 23

AtNRT2.2------------------------------------------------MGSTDEPGSSMHGVTGREQSYA 22

------------------------------------------------------------------------------

OsNRT2.4-------------------------YDYGGYGDGVVDDEGRATELRPMALSRPHTQAFHLAWMSLFACFF 64

TaNRT2-7A------------------------NDWAHIDHG-VDADGRATELRPLALSRPHTQAFHLAWLSLFACFF 59

TaNRT2-7B------------------------SDWAHIDHG-VDADGRATELRPLALSRPHTQAFHLAWLSLFACFF 59

TaNRT2-7D------------------------SDWAHIDHG-VDADGRATELRPLALSRPHTQAFHLAWLSLFACFF 59

AtNRT2.7----------------MEPSQRNTKPPSFSDSTIPVDSDGRATVFRPFSLSSPHSRAFHLAWLSLFSCFF 54

AtNRT2.5----------MEVEGK-GGEAGTTTTTAPRRFALPVDAENKATTFRLFSVAKPHMRAFHLSWFQFFCCFV 59

OsNRT2.3----------MEVEGV-------EAAGGKPRFRMPVDSDLKATEFWLFSFARPHMASFHMAWFSFFCCFV 60

ZmNRT2.5----------MQVEAP-A---EAAAAPSKPRFRMPVDSDNKATEFWLFSFARPHMSAFHMSWFSFFCCFL 66

TaNRT2-3A---------MGVQ-----------AAPKGKFRMPVDSDNKATEFWLFSFARPHMSAFHLSWFSFFCCFV 58

TaNRT2-3B---------MGVQ-----------AAPKGKFRIPVDSDNKATEFWLFSFARPHMSAFHLSWFSFFCCFV 58

TaNRT2-3D---------MGVQ-----------AASKGKFRIPVDSDNKATEFWLFSFARPHMSAFHLSWFSFFCCFV 58

TaNRT2-1D---------MEVQ-----------AAPKAKFKIPVDDDSKATEFWLFSFSRSHMSAFHLSWFSFFCCFV 60

TaNRT2-U.1--------MEVQ-----------AAPKAKFRIPVDDDSKATEFWLFSFARPHMSAFHLSWFSFFCCFV 57

TaNRT2-2A----------MEKMETEM------AAPAKIFPLPVDSEHKAKSFRLFSFAAPHMRAFHLSWMAFFICFV 53

TaNRT2-2D-------------METEM------AAPAKMFPLAVDSEHKAKSFRLFSFAAPHMRAFHLSWMAFFVCFV 50

ZmNRT2.3----------AAHGSSLDG------VTPSSKFDLPVDSEHKAKTIRLLSFANPHMRTFHLSWMSFFTCVV 58

TaNRT2-6B.7-------MEIGSPGAI-------AASTNFSLPVDSEHKAKSIKIFSFGNPHMRAFHLGWMSFFTCVV 55

TaNRT2-6A.7-------MEIGSTGAT-------AASTNFSLPVDSEHKAKSIKIFSFSNPHMRAFHLGWMSFFTCVV 55

TaNRT2-6D.7-------MEIGSTGAT-------AASTNFSLPVDSEHKAKSIKIFSFGNPHMRAFHLGWMSFFTCVV 55

TaNRT2-6D.8-------MEIGSTGAT-------AASTNFSLPVDSEHKAKSIKIFSFGNPHMRAFHLGWMSFFTCVV 55

TaNRT2-6A.8-------MEIGSTGAT-------AASTTFSLPVDSEHKAKSIKIFSFSNPHMRAFHLGWMSFFTCVV 55

TaNRT2-6B.8-------MEIGSTGTT-------AASTNFSLPVDSEHKAKSIKIFSFGNPHMRAFHLGWMSFFTCVV 53

TaNRT2-6A.9-------MEAGSTSD--------TVAGRFSLPVDSENKAKSIKIFSFGNPHMRAFHLGWMSFFTCVV 54

TaNRT2-6B.9-------METGSTGD--------TVAGRFSLPVDSENKAKSIKIFSFGNPHMRAFHLAWMSFFTCVV 54

TaNRT2-6D.9-------MEAGSMGD--------TVAGRFSLPVDSENKAKSIKIFSFGNPHMRAFHLGWMSFFTCVV 54

TaNRT2-6A.13------MEVAST-PT------TPAPINFLLPVDSEHKAKSIKIFSFGNPHMRAFHLGWMSFFTCVV 55

TaNRT2-6B.11------MEVPST-PT------TPAPINFLLPVDSEHKAKSIKIFSFGNPHMRAFHLGWMSFFTCVV 55

TaNRT2-6D.14------MEVAST-PT------TPAPINFLLPVDSEHKAKSIKIFSFGNPHMRAFHLGWMSFFTCVV 55

TaNRT2-6A.12------MEVAST-PT------TPAPINFLLPVDSEHKAKSIKIFSFGNPHMRAFHLGWMSFFTCVV 55

TaNRT2-6D.13------MEVAST-PT------TPAPINFLLPVDSEHKAKSIKIFSFGNPHMRAFHLGWMSFFTCVV 55

TaNRT2-6D.11------MEAGSSAAA------ATPPITFSLPVDSEHKAMCIKIFSFGNPHMRAFHLGWMSFFTCVV 54

TaNRT2-6D.12------MEVGSPDAT------GAAPINFALPVDSEHKAKSIKIFSFGNPHMRAFHLGWMSFFTCVV 56

TaNRT2-6B.10------MEVGSPAAT------TAAPINFSLPVDSEHKAKSIKIFSFGNPHMRAFHLGWMSFFTCVV 56

TaNRT2-6A.11------TEVGSPATA------TAAPINFSLPVDSEHKAKSIKIFSFGNPHMRAFHLGWMSFFTCVV 56

TaNRT2-6D.10------TEVGSPAAA------TAAPINFSLPVDSEHKAKSIKIFSFGNPHMRAFHLGWMSFFTCVV 56

TaNRT2-6A.10------TEVGSPAAA------AAAPIHFSLLVDSEHKAKSIKIFSFGNPHMRAFHLGWMSFFTCVV 56

TaNRT2-U.2----------------------------------------------------MRAFHLGWMSFFTCVV 16

TaNRT2-6D.1-------VESSSHGAG------DEAASKFSLPVDSEHKAKSFRLFSFANPHMRTFHLSWISFFTCFV 56

TaNRT2-6A.6-------VESSSHGAG------DEAASKFSLPVDSEHKAKSFRLFSFANPHMRTFHLSWISFFTCFV 56

TaNRT2-6B.1-------VESSSHGAR------DEAVSKFSLPVDSEHKAKSFRLFSFANPHMRTFHLSWISFFTCFV 56

TaNRT2-6B.6-------VEAG--SHA------D-TASKFTLPVDSEHKAKSFRLFSFANPHMRTFHLSWISFFTCFI 53

TaNRT2-6B.4-------VEAS--AHG------DTAASKFTLPVDSEHKAKSFRLFSFANPHMRTFHLSWISFFTCFV 54

TaNRT2-6D.5------------------------------------------------------------------- 0

TaNRT2-6A.2-------VEAG--AHG------DMAASKFTLPVDSEHKAKSFRLFSFANPHMRTFHLSWISFFTCFV 54

TaNRT2-6B.5-------VEAS--AHG------DTAASKFTLPVDSEHKAKSFRLFSFANPHMRTFHLSWISFFTCFV 112

TaNRT2-6B.3-------VQAG--THG------DTATSKFTLPVDSEHKAKSIRLFSFANPHMRTFHLSWISFFTCFV 54

TaNRT2-6A.1-------VEAS--SHG------DAPASKFSLPVDSEHKAKSFRLFSFANPHMRTFHLSWISFFTCFV 54

TaNRT2-6A.3-------VESS--AHG------DAAASKFTLPVDSEHKAKSFRLFSFANPHMRTFHLSWISFFTCFV 54

TaNRT2-6D.3-------VEAG--AHG------DTAASKFTLPVDSEHKAKSFRLFSFANPHMRTFHLSWISFFTCFV 54

TaNRT2-6D.6-------VEAS--GHG------DAAASKFTLPVDSEHKAKSFRLFSFANPHMRTFHLSWISFFTCFV 54

TaNRT2-6D.4-------VEAG--AHG------DTAASKFTLPVDSEHKAKSFRLFSFANPHMRTFHLSWISFFTCFV 54

TaNRT2-6B.2-------VQAG--SHA------DAAASKFTLPVDSEHKAKSFRLFSFANPHMRTFHLSWISFFTCFV 54

TaNRT2-6A.5-------VQAG--SHA------DSAASKFTLPVDSEHKAKSFRLFSFANPHMRTFHLSWISFFTCFV 54

TaNRT2-6A.4-------VEAG--AHG------DTAASKFTLPVDSEHKAKSFRLFSFANPHMRTFHLSWISFFTCFV 54

TaNRT2-6D.2------------------------------------------------------------------- 0

OsNRT2.1----------FSTEVGGED------AAAASKFDLPVDSEHKAKTIRLLSFANPHMRTFHLSWISFFSCFV 78

OsNRT2.2----------FSTEVGGED------AAAASKFDLPVDSEHKAKTIRLLSFANPHMRTFHLSWISFFSCFV 78

ZmNRT2.1----------FSTEHE-EA------ASNGGKFDLPVDSEHKAKSVRLFSVANPHMRTFHLSWISFFTCFV 75

ZmNRT2.2----------FSTEHE-EA------ASNGGKFDLPVDSEHKAKSVRLFSVANPHMRTFHLSWISFFTCFV 75

AtNRT2.6----------FSVQEDVPSSQAVRTNDPTAKFALPVDSEHRAKVFKPLSFAKPHMRAFHLGWISFFTCFI 87

AtNRT2.3----------FSVDA---SSQTVQSDDPTAKFALPVDSEHRAKVFNPLSFAKPHMRAFHLGWLSFFTCFI 84

AtNRT2.4----------FSVESPAVP------SDSSAKFSLPVDTEHKAKVFKLLSFEAPHMRTFHLAWISFFTCFI 77

AtNRT2.1----------FSVQSPIVH------TDKTAKFDLPVDTEHKATVFKLFSFAKPHMRTFHLSWISFSTCFV 77

AtNRT2.2----------FSVDGSEP-------TNTKKKYNLPVDAEDKATVFKLFSFAKPHMRTFHLSWISFSTCFV 75

------------------------------------------------------------------------------

OsNRT2.4----------AAFAAPPILPAMRPALVLAPSDASAAAVASLSATLVGRLAMGPACDLLGPRRASGVASLV 124

TaNRT2-7A---------AAFAAPPILPALRPALVLAPSDASAAAVASLSAALVGRLAMGAACDLLGPRRASGVASLV 119

TaNRT2-7B---------AAFAAPPILPALRPALVLAPADASAAAVGSLSAALVGRLAMGPACDLLGPRRASGVASLV 119

TaNRT2-7D---------AAFAAPPILPALRPALVLAPSDASAAAVASLSAALVGRLAMGPACDLLGPRRASGVASLV 119

AtNRT2.7----------STFSIPPLVPVISSDLNLSASTVSAAGIASFAGSIFSRLAMGPLCDLIGPRTSSAILSFL 114

AtNRT2.5----------STFAAPPLLPVIRENLNLTATDIGNAGIASVSGAVFARIVMGTACDLFGPRLASAALTLS 119

OsNRT2.3----------STFA------------------------------VFARLAMGTACDLVGPRLASASLILL 90

ZmNRT2.5----------STFAAPPLLPLIRDTLGLTATDIGNAGIASVSGAVFARVAMGTACDLVGPRLASAAIILL 126

TaNRT2-3A---------STFAAPPLLPLIRDNLGLTGKDIGNAGIASVSGAVFARLAMGTACDLVGPRLASAAIILL 118

TaNRT2-3B---------STFAAPPLLPLIRDNLGLTGKDIGNAGIASVSGAVFARLAMGTACDLVGPRLASAAIILL 118

TaNRT2-3D---------STFAAPPLLPLIRDNLGLTGKDIGNAGIASVSGAVFARLAMGTACDLVGPRLASAAIILL 118

TaNRT2-1D---------STFAAPPLMPLIRDNLGLTAKDIGNAGVASVSGAVFARLAMGTACDLVGPRLASAAIILL 120

TaNRT2-U.1--------STFAAPPLMPLIRDNLGLTAKDIGNAGVASVSGAVFARLAMGTACDLVGPRLASAAIILL 117

TaNRT2-2A---------STFAAAPLIPIIRDNLNLTKRDISNASVASVSGSIFSRVAMGVVCDLLGPRYGCAFLVML 113

TaNRT2-2D---------STFAAAPLIPIIRDNLNLTKRDISNASVASVSGSIFSRVAMGVVCDLLGPRYGCAFLVLL 110

ZmNRT2.3----------STFAAAPLIPIIRENLGLTKADIGNAGVASVSGAIFSRLAMGAVCDLLGPRYGCAFVVML 118

TaNRT2-6B.7-------STFAAAPLIPIIRDNLNLTKADIGNAGVASVSGAIFSRLAMGAICDLLGPRYGGAFLIML 115

TaNRT2-6A.7-------STFAAAPLIPIIRDNLNLTKADIGNAGVASVSGAIFSRLAMGAICDLLGPRYGGAFLIML 115

TaNRT2-6D.7-------STFAAAPLIPIIRDNLNLTKADIGNAGVASVSGAIFSRLAMGAICDLLGPRYGGAFLIML 115

TaNRT2-6D.8-------STFAAAPLIPIIRDNLNLTKADIGNAGVASVSGAIFSRLAMGAICDLLGPRYGGAFLIML 115

TaNRT2-6A.8-------STFAAAPLIPIIRDNLNLTKADIGNAGVASVSGAIFSRLAMGAICDLLGPRYGGAFLIML 115

TaNRT2-6B.8-------STFAAAPLIPIIRDNLNLTKADICNAGVASVSGAIFSRLAMGAICDLLGPRYGGAFLIML 113

TaNRT2-6A.9-------STFAAAPLIPIIRDNLNLTKADIGNAGVASVSGAIFSRLAMGAICDLLGPRYGCAFLVML 114

TaNRT2-6B.9-------STFAAAPLIPIIRDNLNLTKADIGNAGVASVSGAIFSRLAMGAICDLLGPRYGCAFLVML 114

TaNRT2-6D.9-------STFAAAPLIPIIRDNLNLTKADIGNAGVASVSGAIFSRLAMGAICDLLGPRYGCAFLVML 114

TaNRT2-6A.13------STFAAAPLIPIIRDNLNLTKADIGNAGVASVSGAIFSRLAMGAICDLLGPRYGCAFLVML 115

TaNRT2-6B.11------STFAAAPLIPIIRDNLNLTKADIGNAGVASVSGAIFSRLAMGAICDLLGPRYGCAFLVML 115

TaNRT2-6D.14------STFAAAPLIPIIRDNLNLTKADIGNAGVASVSGAIFSRLAMGAICDLLGPRYGCAFLVML 115

TaNRT2-6A.12------STFAAAPLIPIIRDNLNLTKADIGNAGVASVSGAIFSRLAMGAICDLLGPRYGCAFLVML 115

TaNRT2-6D.13------STFAAAPLIPIIRDNLNLTKADIGNAGVASVSGAIFSRLAMGAICDLLGPRYGCAFLVML 115

TaNRT2-6D.11------STFAAAPLIPIIRDNLNLTKADIGNAGVASVSGAIFSRLAMGAICDLLGPCYGCAFLVML 114

TaNRT2-6D.12------STFAAAPLIPIIRDNLNLTKADIGNAGVASVSGAIFSRLAMGAICDLLGPRYGCAFLVML 116

TaNRT2-6B.10------STFAAAPLIPIIRDNLNLTKADIGNAGVASVSGAIFSRLAMGAICDLLGPRYGCAFLVML 116

TaNRT2-6A.11------STFAAAPLIPIIRDNLNLTKADIGNAGVASVSGAIFSRLAMGAICDLLGPRYGCAFLVML 116

TaNRT2-6D.10------STFAAAPLIPIIRDNLNLTKADIGNAGVASVSGAIFSRLAMGAICDLLGPRYGCAFLVML 116

TaNRT2-6A.10------STFAAAPLIPIIRDNLNLTKADIGNAGVASVSGAIFSRLAMGAICDLLGPRYGCAFLVML 116

TaNRT2-U.2--------STFAAAPLIPIIRDNLNLTKADIGNAGVASVSGAIFSRLAMGAICDLLGPRYGCAFLVML 76

TaNRT2-6D.1-------STFAAAPLVPIIRDNLNLAKADIGNAGVASVSGSIFSRLAMGAICDLLGPRYGCAFLVML 116

TaNRT2-6A.6-------STFAAAPLVPIIRDNLNLAKADIGNAGVASVSGSIFSRLAMGAICDLLGPRYGCAFLVML 116

TaNRT2-6B.1-------STFAAAPLVPIIRDNLNLAKADIGNAGVASVSGSIFSRLAMGAICDLLGPRYGCAFLVML 116

TaNRT2-6B.6-------STFARRLSSPSSATTSTLPRPTSAM--------------LAWHPCPAPS-------SRGS 92

TaNRT2-6B.4-------STFAAAPLVPIIRDNLNLAKADIGNAGVASVSGSIFSRLAMGAICDLLGPRYGCAFLVML 114

TaNRT2-6D.5------------------------------------------------------------------- 0

TaNRT2-6A.2-------STFAAAPLVPIIRDNLNLAKADIGNAGVASVSGSIFSRLAMGAICDLLGPRYGCAFLVML 114

TaNRT2-6B.5-------STFAAAPLVPIIRDNLNLAKADIGNAGVASVSGSIFSRLAMGAICDLLGPRYGCAFLVML 172

TaNRT2-6B.3-------STFAAAPLVPIIRDNLNLAKADIGNAGVASVSGSIFSRLAMGAVCDLLGPRYGCAFLVML 114

TaNRT2-6A.1-------STFAAAPLVPIIRDNLNLAKADIGNAGVASVSGSIFSRLAMGAICDLLGPRYGCAFLVML 114

TaNRT2-6A.3-------STFAAAPLVPIIRDNLNLAKADIGNAGVASVSGSIFSRLAMGAVCDLLGPRYGCAFLVML 114

TaNRT2-6D.3-------STFAAAPLVPIIRDNLNLAKADIGNAGVASVSGSIFSRLAMGAVCDLLGPRYGCAFLVML 114

TaNRT2-6D.6-------STFAAAPLVPIIRDNLNLAKADIGNAGVASVSGSIFSRLAMGAICDLLGPRYGCAFLVML 114

TaNRT2-6D.4-------STFAAAPLVPIIRDNLNLAKADIGNAGVASVSGSIFSRLAMGAICDLLGPRYGCAFLVML 114

TaNRT2-6B.2-------STFAAAPLVPIIRDNLNLAKADIGNAGVASVSGSIFSRLAMGAICDLLGPRYGCAFLVML 114

TaNRT2-6A.5-------STFAAAPLVPIIRDNLNLAKADIGNAGVASVSGSIFSRLAMGAICDLLGPRYGCAFLVML 114

TaNRT2-6A.4-------STFAAAPLVPIIRDNLNLAKADIGNAGVASVSGSIFSRLAMGAVCDLLGPRYGCAFLVML 114

TaNRT2-6D.2-----------------------------------------------------------------ML 2

OsNRT2.1----------STFAAAPLVPIIRDNLNLTKADIGNAGVASVSGSIFSRLAMGAICDMLGPRYGCAFLIML 138

OsNRT2.2----------STFAAAPLVPIIRDNLNLTKADIGNAGVASVSGSIFSRLAMGAICDMLGPRYGCAFLIML 138

ZmNRT2.1----------STFAAAPLVPIIRDNLNLTKADIGNAGVASVSGSIFSRLTMGAVCDLLGPRYGCAFLIML 135

ZmNRT2.2----------STFAAAPLVPIIRDNLNLTKADIGNAGVASVSGSIFSRLTMGAVCDLLGPRYGCAFLIML 135

AtNRT2.6----------STFAAAPLVPVIRDNLDLTKTDIGNAGVASVSGAIFSRLAMGAVCDLLGARYGTAFSLML 147

AtNRT2.3----------STFAAAPLVPIIRDNLDLTKTDIGNAGVASVSGAIFSRLAMGAVCDLLGARYGTAFSLML 144

AtNRT2.4----------STFAAAPLVPIIRDNLNLTRQDVGNAGVASVSGSIFSRLVMGAVCDLLGPRYGCAFLVML 137

AtNRT2.1----------STFAAAPLVPIIRENLNLTKQDIGNAGVASVSGSIFSRLVMGAVCDLLGPRYGCAFLVML 137

AtNRT2.2----------STFAAAPLIPIIRENLNLTKHDIGNAGVASVSGSIFSRLVMGAVCDLLGPRYGCAFLVML 135

------------------------------------------------------------------------------

OsNRT2.4----------CALALALAAVFASSPAGFVALRFVAGLSLANFVANQHWMSRIFAPSAVGLANAVAAGWAN 184

TaNRT2-7A---------CALALALAAVYASSPAGFVALRFCAGLSLSNFVANQHWMSRIFAPSGVGLANAVAAGWAN 179

TaNRT2-7B---------CALALALAAVYASSPAGFVALRFCAGLSLSNFVANQHWMSRIFAPSGVGLANAVAAGWAN 179

TaNRT2-7D---------CALALALAAVYASSPAGFVALRFCAGLSLSNFVANQHWMSRIFAPSGVGLANAVAAGWAN 179

AtNRT2.7----------TAPVI-LSASLVSSPTSFILVRFFVGFSLANFVANQYWMSSMFSGNVIGLANGVSAGWAN 173

AtNRT2.5----------TAPAV-YFTAGIKSPIGFIMVRFFAGFSLATFVSTQFWMSSMFSGPVVGSANGIAAGWGN 178

OsNRT2.3----------TTPAV-YCSSIIQSPSGYLLVRFFTGISLASFVSAQFWMSSMFSAPKVGLANGVAGGWGN 149

ZmNRT2.5----------TTPAV-YYSAVIDSASSYLLVRFFTGFSLASFVSTQFWMSSMFSPPKVGLANGVAGGWGN 185

TaNRT2-3A---------TTPAV-YCSAIIDSASSFLLVRFFTGFSLASFVSTQFWMSSMFSSPKVGLANGVAGGWGN 177

TaNRT2-3B---------TTPAV-YCSAIIDSASSFLLVRFFTGFSLASFVSTQFWMSSMFSSPKVGLANGVAGGWGN 177

TaNRT2-3D---------TTPAV-YCSAIIDSASSFLLVRFFTGFSLASFVSTQFWMSSMFSSPKVGLANGVAGGWGN 177

TaNRT2-1D---------TTPAV-YCTSIINSASSFLLARFFTGFSLASFVSTQFWMSSMFSAPKVGLANGVAGGWGN 179

TaNRT2-U.1--------TTPAV-YCTSIINSASSFLLARFFTGFSLASFVSTQFWMSSMFSAPKVGLANGVAGGWGN 176

TaNRT2-2A---------TAPAV-FCMSLVHDPAGYIMVRFLIGFSLATLISCQYWMSTMFSGNIIGAVNGLAAGWGN 172

TaNRT2-2D---------TAPAV-FCMSLVHDPAGYIMVRFLIGFSLATLISCQYWMSTMFSGNIIGAVNGLAAGWGN 169

ZmNRT2.3----------AAPAV-FCMAVIDSAAGYVACRFLIGFSLATFVSCQYWTSTMFNIKIIGTVNALASGWGD 177

TaNRT2-6B.7-------SAPAV-FCMSVIDSPAGYIIVRFLIGVSLATFVSCQYWISTMFNSKIIGTVGGLTAGWGD 174

TaNRT2-6A.7-------SAPAV-FCMSVIDSPAGYITVRFLIGVSLATFVSCQYWISTMFNSKIIGTVGGLTAGWGD 174

TaNRT2-6D.7-------SAPAV-FCMSVVDSPAGYIIVRFLIGVSLATFVSCQYWISTMFNSKIIGTVGGLTAGWGD 174

TaNRT2-6D.8-------SAPAV-FCMSVIDSPAGYITVRFLIGVSLATFVSCQYWISTMFNSKIIGTVGGLTAGWGD 174

TaNRT2-6A.8-------SAPAV-FCMSVIDSPAGYITVRFLIGVSLATFVSCQYWISTMFNSKIIGTVGGLTAGWGD 174

TaNRT2-6B.8-------SAPAV-FCMSVIDSPAGYITVRFLIGVSLATFVSCQYWISTMFNSKIIGTVGGLTAGWGD 172

TaNRT2-6A.9-------SAPAV-FCMSIIDGPAGYITIRFLIGVSLATFVSCQYWVSTMFNSKIIGTVGGLTAGWGD 173

TaNRT2-6B.9-------SAPAV-FCMSIIDGPAGYITIRFLIGVSLATFVSCQYWVSTMFNSKIIGTVGGLTAGWGD 173

TaNRT2-6D.9-------SAPAV-FCMSIIDGPAGYITIRFLIGVSLATFVSCQYWVSTMFNSKIIGTVGGLTAGWGD 173

TaNRT2-6A.13------SAPAV-FCMSVIDGPSGYITIRFLIGVSLATFVSCQYWVSTMFNSKIIGTVGGLTAGWGD 174

TaNRT2-6B.11------SAPAV-FCMSVIDGPGGYITIRFLIGVSLATFVSCQYWVSTMFNSKIIGTVGGLTAGWGD 174

TaNRT2-6D.14------SAPAV-FCMSVIDGPSGYITIRFLIGVSLATFVSCQYWVSTMFNSKIIGTVGGLTAGWGD 174

TaNRT2-6A.12------SAPAV-FCMSIIDGPGGYITIRFLIGVSLATFVSCQYWVSTMFNSKIIGTVGGLTAGWGD 174

TaNRT2-6D.13------SAPAV-FCMSVIDGPGGYITIRFLIGISLATFVSCQYWVSTMFNSKIIGTVGGLTAGWGD 174

TaNRT2-6D.11------SAPAV-FCMSVIDGPGGYITIRFLIGVSLATFVSCQYWISTMFSSKIIGMVGGLMAGWGD 173

TaNRT2-6D.12------SAPAV-FCMSVIDGPGGYITIRFLIGVSLATFVSCQYWISTMFNSKIIGTVGGLTAGWGD 175

TaNRT2-6B.10------SAPAV-FCMSVIDGPGGYITIRFLIGVSLATFVSCQYWISTMFNSKIIGTVGGLTAGWGD 175

TaNRT2-6A.11------SAPAV-FCMSVIDGPGGYITIRFLIGVSLATFVSCQYWISTMFNSKIIGTVGGLTAGWGD 175

TaNRT2-6D.10------SAPAV-FCMSVIDGPGGYITIRFLIGVSLATFVSCQYWISTMFNSKIIGTVGGLTAGWGD 175

TaNRT2-6A.10------SAPAV-FCMSVIDGPGGYITIRFLIGVSLATFVSCQYWISTMFNSKIIGTVGGLTAGWGD 175

TaNRT2-U.2--------SAPAV-FCMSVIDGPGGYITIRFLIGVSLATFVSCQYWISTMFNSKIIGTVGGLTAGWGD 135

TaNRT2-6D.1-------AAPTV-FCMSLIDDAAGYITVRFLIGFSLATFVSCQYWMSTMFNSKIIGTVNGLAAGWGN 175

TaNRT2-6A.6-------AAPTV-FCMSLIDDAAGYITVRFLIGFSLATFVSCQYWMSTMFNSKIIGTVNGLAAGWGN 175

TaNRT2-6B.1-------AAPTV-FCMSLIDDAAGYITVRFLIGFSLATFVSCQYWMSTMFNSKIIGTVNGLAAGWGN 175

TaNRT2-6B.6-------PWEPF-FCMAVIDDASGYIAVRFLIGFSLATFVSCQYWMSTMFNSKIIGTVNGLAAGWGN 151

TaNRT2-6B.4-------SAPTV-FCMAAIDDASGYIAVRFLIGFSLATFVSCQYWMSTMFNSKIIGTVNGLAAGWGN 173

TaNRT2-6D.5------------------------------------------------------------------- 0

TaNRT2-6A.2-------SAPTV-FCMAVIDDASGYIAVRFLIGFSLATFVSCQYWMSTMFNSKIIGTVNGLAAGWGN 173

TaNRT2-6B.5-------SAPTV-FCMAVIDDASGYIAVRFLIGFSLATFVSCQYWMSTMFNSKIIGTVNGLAAGWGN 231

TaNRT2-6B.3-------SAPTV-FCMAVIDDASGYIAVRFLIGFSLATFVSCQYWMSTMFNSKIIGTVNGLAAGWGN 173

TaNRT2-6A.1-------SAPTV-FCMAVIDDASGYIAVRFLIGFSLATFVSCQYWMSTMFNSKIIGTVNGLAAGWGN 173

TaNRT2-6A.3-------SAPTV-FCMAVIDDASGYIAVRFLIGFSLATFVSCQYWMSTMFNSKIIGTVNGLAAGWGN 173

TaNRT2-6D.3-------SAPTV-FCMAVIDDASGYIAVRFLIGFSLATFVSCQYWMSTMFNSKIIGTVNGLAAGWGN 173

TaNRT2-6D.6-------SAPTV-FCMAVIDDASGYIAVRFLIGFSLATFVSCQYWMSTMFNSKIIGTVNGLAAGWGN 173

TaNRT2-6D.4-------SAPTV-FCMAVIDDASGYIAVRFLIGFSLATFVSCQYWMSTMFNSKIIGTVNGLAAGWGN 173

TaNRT2-6B.2-------SAPTV-FCMAVIDDASGYIAVRFLIGFSLATFVSCQYWMSTMFNSKIIGTVNGLAAGWGN 173

TaNRT2-6A.5-------SAPTV-FCMSVIDDASGYIAVRFLIGFSLATFVSCQYWMSTMFNSKIIGTVNGLAAGWGN 173

TaNRT2-6A.4-------SAPTV-FCMAVIDDASGYIAVRFLIGFSLATFVSCQYWMSTMFNSKIIGTVNGLAAGWGN 173

TaNRT2-6D.2-------SAPTV-FCMAVIDDASGYIAVRFLIGFSLATFVSCQYWMSTMFNSKIIGTVNGLAAGWGN 61

OsNRT2.1----------AAPTV-FCMSLIDSAAGYIAVRFLIGFSLATFVSCQYWMSTMFNSKIIGLVNGLAAGWGN 197

OsNRT2.2----------AAPTV-FCMSLIDSAAGYIAVRFLIGFSLATFVSCQYWMSTMFNSKIIGLVNGLAAGWGN 197

ZmNRT2.1----------SAPTV-FCMSLIDDAAGYITVRFLIGFSLATFVSCQYWMSTMFSSKIIGTVNGLAAGWGN 194

ZmNRT2.2----------SAPTV-FCMSLIDDAAGYIAVRFLIGFSLATFVSCQYWMSTMFSSKIIGTVNGLAAGWGN 194

AtNRT2.6----------TAPAV-FSMSFVADAGSYLAVRFMIGFCLATFVSCQYWTSVMFTGKIIGLVNGCAGGWGD 206

AtNRT2.3----------TAPTV-FSMSFVGGPSGYLGVRFMIGFCLATFVSCQYWTSVMFNGKIIGLVNGCAGGWGD 203

AtNRT2.4----------SAPTV-FSMSFVGGAGGYITVRFMIGFCLATFVSCQYWMSTMFNGQIIGLVNGTAAGWGN 196

AtNRT2.1----------SAPTV-FSMSFVSDAAGFITVRFMIGFCLATFVSCQYWMSTMFNSQIIGLVNGTAAGWGN 196

AtNRT2.2----------SAPTV-FSMSFVSDAAGFITVRFMIGFCLATFVSCQYWMSTMFNSQIIGLVNGTAAGWGN 194

------------------------------------------------------------------------------

OsNRT2.4----------VGSAAAQVVMPVAYDAVVLRLGVPVTVAWRVTYLLPCAMLVTTGLAVLAFPYDLPGGGGG 244

TaNRT2-7A---------VGSAAAQVVMPLAYDLIVLRLGVPITVAWRVAYLIPCAMLITTGLAVLAFPYDLPSGCA- 238

TaNRT2-7B---------VGSAAAQVVMPLAYDLIVLRLGVPITVAWRVAYLIPCAMLITTGLAVLAFPYDLPSGCA- 238

TaNRT2-7D---------VGSAAAQVVMPLAYDLIVLRLGVPITVAWRVAYLIPCAMLIATGLAVLAFPYDLPSGCT- 238

AtNRT2.7----------VGAGISQLLMPLIYSTIAEF--LPRAVAWRVSFVFPAIFQVTTAVLVLLYGQDTPHGNRK 231

AtNRT2.5----------LGGGATQLIMPIVFSLIR-NMGATKFTAWRIAFFIPGLFQTLSAFAVLLFGQDLPDGDYW 237

OsNRT2.3----------LGGGAVQLLMPLVYEAIH-KIGSTPFTAWRIAFFIPGLMQTFSAIAVLAFGQDMPGGNYG 208

ZmNRT2.5----------LGGGAVQLIMPLVFEAIR-KAGATPFTAWRVAFFVPGLLQTLSAVAVLAFGQDMPDGNYR 244

TaNRT2-3A---------LGGGAVQFIMPLVYEIVR-KIGSTDFVAWRIAFFIPGIMQTFSAIAVLAFGQDMPDGNYR 236

TaNRT2-3B---------LGGGAVQFIMPLVFEVVR-KIGSTDFVAWRVAFFIPGIMQTFSAIAVLAFGQDMPDGNYR 236

TaNRT2-3D---------LGGGAVQFIMPLVFEVVR-KIGSTDFVAWRVAFFIPGIMQTFSAIAVLAFGQDMPDGNYR 236

TaNRT2-1D---------LGGGAVQLLMPFVFEAVR-KIGSTKFVAWRVAFFIPGIMQTVSAIAVLALGQDMPDGNYR 238

TaNRT2-U.1--------LGGGAVQLLMPFVFEAVR-KMGSTKFVAWRVAFFIPGIMQTVSAIAVLALGQDMPDGNYR 235

TaNRT2-2A---------VGGGATQLVMPLVYEAIRSRCGATPFSAWRVAYFGPGTLHIVVGIMVLTLGQDLPDGNLW 232

TaNRT2-2D---------VGGGATQLIMPLVYEAIRSRCGATPFSAWRVAYFGPGTLHIVVGIMVLTLGQDLPDGNLW 229

ZmNRT2.3----------MGGGATQLIMPFVYEAIL-RCGATPFAAWRIAYFVPGIMHIAVGILVLTAGQDLPDGNLR 236

TaNRT2-6B.7-------MGGGATQLIMPFVFDAIK-ACGATRFTAWRIAYFVPGMMLVVIGLLVLTLGQDLPDGNLR 233

TaNRT2-6A.7-------MGGGATQLIMPFVFDAIK-ACGATRFTAWRIAYFVPGMMLVVMGLLVLTLGQDLPDGNLR 233

TaNRT2-6D.7-------MGGGATQLIMPFVFDAIK-ACGATRFTAWRIAYFVPGMMLVVMGLLVLTLGQDLPDGNLR 233

TaNRT2-6D.8-------MGGGATQLIMPFVFDAIK-ACGATRFTAWRIAYFVPGMMLVVMGLLVLTLGQDLPDGNLR 233

TaNRT2-6A.8-------MGGGATQLIMPFVFDAIK-ACGATRFTAWRIAYFVPGMMLVVMGLLVLTLGQDLPDGNLR 233

TaNRT2-6B.8-------MGGGATQLIMPFVFDAIK-ACGATRFTAWRIAYFVPGMMLVVMGLLVLTLGQDLPDGNLR 231

TaNRT2-6A.9-------MGGGATQLIMPLVFDAII-ACDATPFTAWRIAYFVPGLMLVVMGLLVLTMGQDLPDGNLR 232

TaNRT2-6B.9-------MGGGATQLIMPLVFDAII-ACGATPFTAWRIAYFVPGLMLVVMGLLVLTMGQDLPDGNLR 232

TaNRT2-6D.9-------MGGGATQLIMPLVFDAII-ACGATPFTAWRIAYFVPGLMLVVMGLLVLTTGQDLPDGNMR 232

TaNRT2-6A.13------MGGGATQLIMPLVFDAIL-ACGATPFMAWRLAYFVPGMMLVVMGLLVLTMGQDLPDGNLR 233

TaNRT2-6B.11------MGGGATQLIMPLIFDAIL-ACGATPFTAWRLAYFVPGMMLVVMGLLVLTMGQDLPDGNLR 233

TaNRT2-6D.14------MGGGATQLIMPLVFDAIL-ACGATPFTAWRLAYFVPGMMLVVMGLLVLTMGQDLPDGNLR 233

TaNRT2-6A.12------MGGGATQLIMPLVFDAIL-ACGATPFTAWRLAYFVPGMMLVVMGLLVLTMGQDLPDGNLR 233

TaNRT2-6D.13------MGGGATQLIMPLVFDAIL-ACGATPFTAWRLAYFVPGMMLVVMGLLVLTMGQDLPDGNLR 233

TaNRT2-6D.11------MGGGATQLIMPLVFDGIL-ACGATRFTAWRIAYFVPGMMLVLMGLLVLTMGQDLPDGNLR 232

TaNRT2-6D.12------MGGGATQLIMPLVFDGIL-ACGATRFTAWRLAYFVPGMMLVVMGLLVLTMGQDLPDGNLG 234

TaNRT2-6B.10------MGGGATQLIMPLVFDGIL-ACGATPFTAWRLAYFVPGMMLVVMGLLVLTLGQDLPDGNLR 234

TaNRT2-6A.11------MGGGATQLIMPLVFDGIL-ACGATRFTAWRIAYFVPGMMLVVMGLLVLTMGQDLPDGNLR 234

TaNRT2-6D.10------MGGGATQLIMPLVFDGIL-ACGATSFMAWRIAYFVPGMMLVVMGLLVLTMGQDLPDGNLR 234

TaNRT2-6A.10------MGGGATQLIMPLVFDGIL-ACGATSFTAWRIAYFVPGMMLVVMGLLVLTMGQDLPDGNLR 234

TaNRT2-U.2--------MGGGATQLIMPLVFDGIL-ACGATSFTAWRIAYFVPGMMLVVMGLLVLTMGQDLPDGNLR 194

TaNRT2-6D.1-------MGGGATQLIMPLVFHAIQ-KCGATPFVAWRIAYFVPGLMHVVMGLLVLTMGQDLPDGNLA 234

TaNRT2-6A.6-------MGGGATQLIMPLVFHAIQ-KCGATPFVAWRIAYFVPGMMHIVMGLMVLTMGQDLPDGNLA 234

TaNRT2-6B.1-------MGGGATQLIMPLVFHAIQ-KCGATPFVAWRIAYFVPGMMHIVMGLMVLTMGQDLPDGNLA 234

TaNRT2-6B.6-------MGGGATQLIMPLVFHAIQ-KCGATPFVAWRIAYFVPGMMHIVMGLLVLTMGQDLPDGNLA 210

TaNRT2-6B.4-------MGGGATQLIMPLVFHAIQ-KCGATPFVAWRIAYFVPGMMHIVMGLLVLTMGQDLPDGNLA 232

TaNRT2-6D.5-------------------------------------MAYFVPGMMHIVMGLLVLTMGQDLPDGNLA 30

TaNRT2-6A.2-------MGGGATQLIMPLVFHAIQ-KCGATPFVAWRIAYFVPGMMHIVMGLLVLTMGQDLPDGNLA 232

TaNRT2-6B.5-------MGGGATQLIMPLVFHAIQ-KCGATPFVAWRIAYFVPGMMHIVMGLLVLTMGQDLPDGNLA 290

TaNRT2-6B.3-------MGGGATQLIMPLVFHAIQ-KCGATPFVAWRIAYFVPGMMHIVMGLLVLTLGQDLPDGNLA 232

TaNRT2-6A.1-------MGGGATQLIMPLVFHAIQ-KCGATPFVAWRIAYFVPGMMHIVMGLLVLTMGQDLPDGNLA 232

TaNRT2-6A.3-------MGGGATQLIMPLVFHAIQ-KCGATPFVAWRIAYFVPGMMHIVMGLLVLTMGQDLPDGNLA 232

TaNRT2-6D.3-------MGGGATQLIMPLVFHAIQ-KCGATPFVAWRIAYFVPGMMHIVMGLLVLTMGQDLPDGNLA 232

TaNRT2-6D.6-------MGGGATQLIMPLVFHAIQ-KCGATPFVAWRIAYFVPGMMHIVMGLLVLTMGQDLPDGNLA 232

TaNRT2-6D.4-------MGGGATQLIMPLVFHAIQ-KCGATPFVAWRIAYFVPGMMHIVMGLLVLTMGQDLPDGNLA 232

TaNRT2-6B.2-------MGGGATQLIMPLVFHAIQ-KCGATPFVAWRIAYFVPGMMHIVMGLLVLTMGQDLPDGNLA 232

TaNRT2-6A.5-------MGGGATQLIMPLVFHAIQ-KCGATPFVAWRIAYFVPGMMHIVMGLLVLTMGQDLPDGNLA 232

TaNRT2-6A.4-------MGGGATQLIMPLVFHAIQ-KCGATPFVAWRIAYFVPGMMHIVMGLLVLTMGQDLPDGNLA 232

TaNRT2-6D.2-------MGGGATQLIMPLVFHAIQ-KCGATPFVAWRIAYFVPGMMHIVMGLLVLTMGQDLPDGNLA 120

OsNRT2.1----------MGGGATQLIMPLVYDVIR-KCGATPFTAWRLAYFVPGTLHVVMGVLVLTLGQDLPDGNLR 256

OsNRT2.2----------MGGGATQLIMPLVYDVIR-KCGATPFTAWRLAYFVPGTLHVVMGVLVLTLGQDLPDGNLR 256

ZmNRT2.1----------MGGGATQLIMPLVYDVIR-KCGATPFTAWRLAYFVPGLMHVVMGVLVLTLGQDLPDGNLR 253

ZmNRT2.2----------MGGGATQLIMPLVYDVIR-KCGATPFTAWRLAYFVPGLMHVVMGVLVLTLGQDLPDGNLR 253

AtNRT2.6----------MGGGVTQLLMPMVFHVIK-LTGATPFTAWRFAFFIPGILQIVMGILVLTLGQDLPDGNLS 265

AtNRT2.3----------MGGGVTQLLMPMVFHVIK-LAGATPFMAWRIAFFVPGFLQVVMGILVLSLGQDLPDGNLS 262

AtNRT2.4----------MGGGVTQLLMPMVYEIIR-RLGSTSFTAWRMAFFVPGWMHIIMGILVLTLGQDLPDGNRS 255

AtNRT2.1----------MGGGITQLLMPIVYEIIR-RCGSTAFTAWRIAFFVPGWLHIIMGILVLNLGQDLPDGNRA 255

AtNRT2.2----------MGGGITQLLMPIVYEIIR-RCGSTAFTAWRIAFFVPGWLHIIMGILVLTLGQDLPGGNRA 253

------------------------------------------------.::.-*--:----..-**----*-*-*---

OsNRT2.4----------RCPGGGG-------------GRRRSFWAVVRGGVGDYRAWLLGLTYGHCYGVELIMENVA 291

TaNRT2-7A---------YAGGGKG-------------AKGEGFWKVVRGGVCDYRAWVLALTYGYCYGVELIMENVA 285

TaNRT2-7B---------YAGGAKR-------------AKGEGFWNVVRGGVSDYRAWVLALTYGYCYGVELIMENVA 285

TaNRT2-7D---------YAGGA----------------KGEGFWKVVRGGVSDYRAWVLALTYGYCYGVELIMENVA 282

AtNRT2.7----------NSNQNKLTIPEEEEVLVVEEDERSSFVEILIGGLGNYRAWILALLYGYSYGVELTTDNVI 291

AtNRT2.5----------AMHKSGE-------------REKDDVGKVISNGIKNYRGWITALAYGYCFGVELTIDNII 284

OsNRT2.3----------KLHKTGD-------------MHKDSFGNVLRHALTNYRGWILALTYGYSFGVELTIDNVV 255

ZmNRT2.5----------KLHRSGD-------------MHKDSFGNVLRHAVTNYRAWILALTYGYCFGVELAVDNIV 291

TaNRT2-3A---------KLHKSGE-------------MHKDSFGNVLRHAVTNYRAWILALTYGYCFGVELAVDNIV 283

TaNRT2-3B---------KLHKSGE-------------MHKDSFGNVLRHAVTNYRAWILALTYGYCFGVELAVDNIV 283

TaNRT2-3D---------KLHKSGE-------------MHKDSFGNVLRHAVTNYRAWILALTYGYCFGVELAVDNIV 283

TaNRT2-1D---------KLHKSGE-------------MHKDSFGNVLRHAVTNYRAWILALTYGYSFGVELAVDNIV 285

TaNRT2-U.1--------KLHKSGE-------------MHKDSFGNVLRHAVTNYRAWILALTYGYSFGVELAVDNIV 282

TaNRT2-2A---------SLQNKGQ-------------VAKDKFAKVAWGAITNYRSWVFVLLYGYSAGVELCTDNVI 279

TaNRT2-2D---------SLQNKGQ-------------VAKDKFAKVAWGAITNYRSWIFVLLYGYSAGVELCTNNVI 276

ZmNRT2.3----------SLRKQQQQQQQ-GDGGDASCCRRDSFSRVLWHAVANYRTWVFVFVYGYSMGVQLTTNNII 295

TaNRT2-6B.7-------NLQKNGD-------------MNKDKFSKVLRGAVTNYRTWIFVFIYGYCMGVELTSNNVI 280

TaNRT2-6A.7-------NLQKNGD-------------MNKDKFSKVLRGAVTNYRTWIFVFIYGYCMGVELTSNNVI 280

TaNRT2-6D.7-------NLQKNGD-------------MNKDKFSKVLRGAVTNYRTWIFVFIYGYCMGVELTSNNVI 280

TaNRT2-6D.8-------SLQKNGG-------------MNKDKFSKVLRGAITNYRTWIFVFIYGYCMGVELTSNNVI 280

TaNRT2-6A.8-------SLQKNGG-------------MNKDKFSKVLRGAITNYRTWIFVFIYGYCMGVELTSNNVI 280

TaNRT2-6B.8-------SLQKNGG-------------MNKDKFSKVLRGAITNYRTWIFVFIYGYCMGVELTSNNVI 278

TaNRT2-6A.9-------SLQKNGD-------------MNKDKFSNVLRGAVTNYRTWIFVFIYGYCMGVELTTNNVI 279

TaNRT2-6B.9-------SLQKNGD-------------MNKDKFSNVLRGAVTNYRTWIFVFIYGYCMGVELTTNNVI 279

TaNRT2-6D.9-------SLQKNGD-------------MNKDKFSNVLRGAVTNYRTWIFVFIYGYCMGVELTTNNVI 279

TaNRT2-6A.13------SLQKNGD-------------MNKDKFSNVLRGAVTNYRTWIFVFIYGYCMGVELTTNNVI 280

TaNRT2-6B.11------SLQKNGD-------------MNKDKFSNVLRGAVTNYRTWIFVFIYGYCMGVELTTNNVI 280

TaNRT2-6D.14------SLQKNGD-------------MNKDKFSNVLRGAVTNYRTWIFVFIYGYCMGVELTTNNVI 280

TaNRT2-6A.12------SLQKNGD-------------MNKDKFSNVLRGAVTNYRTWIFVFIYGYCMGVELTTNNVI 280

TaNRT2-6D.13------SLQKNGD-------------MNKDKFSNVLRGAVTNYRTWIFVFIYGYCMGVELTTNNVI 280

TaNRT2-6D.11------SLQKNGD-------------MNKDKFSKVLRGAVTNYRTWIFVFIYGYCMGVELTTNNVI 279

TaNRT2-6D.12------SLQKNGD-------------MNKDKFSKVLRGAVTNYRTWIFVFIYGYCMGVELTTNNVI 281

TaNRT2-6B.10------SLQKNGD-------------MNKDKFTKVLQGAVTNYRTWIFVFIYGYCMGVELTTNNVI 281

TaNRT2-6A.11------SLQKNGD-------------MNKDKFSNVLRGAVTNYRTWIFVFIYGYCMGVELTTNNVI 281

TaNRT2-6D.10------SLQKNGD-------------MNKDKFSKVLRGAVTNYRTWIFVFIYGYCMGVELTTNNVI 281

TaNRT2-6A.10------SLQKNGD-------------MNKDKFTKVLRGAVTNYRTWIFVFIYGYCMGVELTTNNVI 281

TaNRT2-U.2--------NLQKNGD-------------MNKDKFTKVLRGAVTNYRTWIFVFIYGYCMGIELTTNNVI 241

TaNRT2-6D.1-------SLQKKGD-------------MAKDKFSKVVWGAVTNYRTWIFVLLYGYCMGVELTTDNVI 281

TaNRT2-6A.6-------SLQKKGD-------------VAKDKFSKVLWGAVTNYRTWIFVLLYGYCMGVELTTDNVI 281

TaNRT2-6B.1-------SLQKKGD-------------MAKDKFSKVVWGAVTNYRTWIFVLLYGYCMGVELTTDNVI 281

TaNRT2-6B.6-------SLQKKGD-------------MAKDKFSKVLWGAVTNYRTWIFVLLYGYCMGVELTTDNVI 257

TaNRT2-6B.4-------SLQKKGD-------------MAKDKFSKVLWGAVTNYRTWIFVLLYGYCMGVELTTDNVI 279

TaNRT2-6D.5-------SLQKKGD-------------MAKDKFSKVLWGAVTNYRTWIFVLLYGYCMGVELTTDNVI 77

TaNRT2-6A.2-------SLQKKGD-------------MAKDKFSKVLWGAVTNYRTWIFVLLYGYCMGVELTTDNVI 279

TaNRT2-6B.5-------SLQKKGD-------------MAKDKFSKVLWGAVTNYRTWIFVLLYGYCMGVELTTDNVI 337

TaNRT2-6B.3-------SLQKKGD-------------MAKDKFSKVLWGAVTNYRTWIFVLLYGYCMGVELTTDNVI 279

TaNRT2-6A.1-------SLQKKGD-------------MAKDKFSKVLWGAVTNYRTWIFVLLYGYCMGVELTTDNVI 279

TaNRT2-6A.3-------SLQKKGD-------------MAKDKFSKVLWGAVTNYRTWIFVLLYGYCMGVELTTDNVI 279

TaNRT2-6D.3-------SLQKKGD-------------MAKDKFSKVLWGAVTNYRTWIFVLLYGYCMGVELTTDNVI 279

TaNRT2-6D.6-------SLQKKGD-------------MAKDKFSKVLWGAVTNYRTWIFVLLYGYCMGVELTTDNVI 279

TaNRT2-6D.4-------SLQKKGD-------------MAKDKFSKVLWGAVTNYRTWIFVLLYGYCMGVELTTDNVI 279

TaNRT2-6B.2-------SLQKKGD-------------MAKDKFSKVLWGAVTNYRTWIFVLLYGYCMGVELTTDNVI 279

TaNRT2-6A.5-------SLQKKGD-------------MAKDKFSKVLWGAVTNYRTWIFVLLYGYCMGVELTTDNVI 279

TaNRT2-6A.4-------SLQKKGD-------------MAKDKFSKVLWGAVTNYRTWIFVLLYGYCMGVELTTDNVI 279

TaNRT2-6D.2-------SLQKKGD-------------MAKDKFSKVLWGAVTNYRTWIFVLLYGYCMGVELTTDNVI 167

OsNRT2.1----------SLQKKGD-------------VNRDSFSRVLWYAVTNYRTWIFVLLYGYSMGVELTTDNVI 303

OsNRT2.2----------SLQKKGD-------------VNRDSFSRVLWYAVTNYRTWIFVLLYGYSMGVELTTDNVI 303

ZmNRT2.1----------SLQKKGN-------------VNKDSFSKVMWYAVINYRTWIFVLLYGYCMGVELTTDNVI 300

ZmNRT2.2----------SLQKKGN-------------VNKDSFSKVMWYAVINYRTWIFVLLYGYCMGVELTTDNVI 300

AtNRT2.6----------TLQKSGQ-------------VSKDKFSKVFWFAVKNYRTWILFMLYGFSMGVELTINNVI 312

AtNRT2.3----------TLQKSGQ-------------VSKDKFSKVFWFAVKNYRTWILFVLYGSSMGIELTINNVI 309

AtNRT2.4----------TLEKKGA-------------VTKDKFSKVLWYAITNYRTWVFVLLYGYSMGVELTTDNVI 302

AtNRT2.1----------TLEKAGE-------------VAKDKFGKILWYAVTNYRTWIFVLLYGYSMGVELSTDNVI 302

AtNRT2.2----------AMEKAGE-------------VAKDKFGKILWYAVTNYRTWIFVLLYGYSMGVELSTDNVI 300

-------------------------------------------.--:---.:-:**-*:--.-**-.-*::*--:*:-

OsNRT2.4----------ADFFRRRFRLPMEAAGAAAACFGAMNAVARPAGGVASDEVARRFGMRGRLWALWAVQSAG 351

TaNRT2-7A---------ADFFRRRFRLPMEAAGAAAACFGVMNTVARPAGGVASDVVGRRFGMRGRLWALWAVQSTG 345

TaNRT2-7B---------ADFFRRRFRLPMEAAGAAAACFGVMNTVARPAGGVASDEVGRRFGMRGRLWALWAVQSTG 345

TaNRT2-7D---------ADFFRRRFRLPMEAAGAAAACFGVMNTVARPAGGVASDVVGRRFGMRGRLWALWAVQSTG 342

AtNRT2.7----------AGYFYERFGVNLEAAGTIAASFGISNIASRPAGGMISDALGKRFGMRGRLWGLWIVQSVA 351

AtNRT2.5----------AEYFFDRFHLKLQTAGIIAASFGLANFFARPGGGIFSDFMSRRFGMRGRLWAWWIVQTSG 344

OsNRT2.3----------HQYFYDRFDVNLQTAGLIAASFGMANIISRPGGGLLSDWLSSRYGMRGRLWGLWTVQTIG 315

ZmNRT2.5----------AQYFYDRFGVKLSTAGFIAASFGMANIVSRPGGGLLSDWLSSRFGMRGRLWGLWVVQTIG 351

TaNRT2-3A---------AQYFYDRFDVNLHTAGLIAASFGMANIISRPGGGLMSDWLSDRFGMRGRLWGLWVVQTIG 343

TaNRT2-3B---------AQYFYDRFDVNLHTAGLIAASFGMANIISRPGGGLMSDWLSDRFGMRGRLWGLWIVQTIG 343

TaNRT2-3D---------AQYFYDRFDVNLHTAGLIAASFGMANIISRPGGGLMSDWLSDRFGMRGRLWGLWIVQTIG 343

TaNRT2-1D---------AEYFYDRFDVNLHTAGLIAATFGLANIVSRPGGGLMSDWLSQRYGMRGRLWGLWVMQTIG 345

TaNRT2-U.1--------AEYFYDRFDVNLKTAGLIAATFGLANIVSRPGGGLMSDWLSQRYGMRGRLWGLWVMQTIG 342

TaNRT2-2A---------AEYYYDHFHLGLRTAGTIAASFGLANIFVRSMGGYFSDVGARYFGMRARLWNIWILQTAG 339

TaNRT2-2D---------AEYYYDHFHLGLRTAGTIAASFGLANIFVRSMGGYFSDVGARYFGMRARLWNIWILQTAG 336

ZmNRT2.3----------AEFYYDQFELDIRVAGIIAACFGMANLVSRPLGGVLSDLGARYWGMRARLWNIWILQTAG 355

TaNRT2-6B.7-------AGYYYDSFYLDLRKAGIIAACFGLANIFARPMGGYLSDLGARYFGMRARLWNIWILQTAG 340

TaNRT2-6A.7-------AGYYYDSFYLDLRKAGIIAACFGLANIFARPMGGYLSDLGARYFGMRARLWNIWILQTAG 340

TaNRT2-6D.7-------AGYYYDSFYLDLRKAGIIAACFGLANIFARPMGGYLSDLGARYFGMRARLWNIWILQTAG 340

TaNRT2-6D.8-------AGYYYDSFYLDLRKAGIIAACFGLANIFARPMGGYLSDLGARYFGMRARLWNIWILQTAG 340

TaNRT2-6A.8-------AGYYYDSFYLDLRKAGIIAACFGLANIFARPMGGYLSDLGARYFGMRARLWNIWILQTAG 340

TaNRT2-6B.8-------AGYYYDSFYLDLRKAGIIAACFGLANIFARPMGGYLSDLGARYFGMRARLWNIWILQTAG 338

TaNRT2-6A.9-------AEYYYDSFHLDLRAAGTIAACFGLVNIFARPMGGYLSDLGARYFGMRARLWNIWILQTAG 339

TaNRT2-6B.9-------AEYYYDSFHLDLRAAGTIAACFGLANIFARPMGGYLSDLGARYFGMRARLWNIWILQTAG 339

TaNRT2-6D.9-------AEYYYDSFHLDLRAAGTIAASFGLANIFARPMGGYLSDLGARYFGMRARLWNIWILQTAG 339

TaNRT2-6A.13------AEYYYDSFHLDLRAAGTIAACFGLANIFARPMGGYLSDLSARYFGMRARLWNIWILQTAG 340

TaNRT2-6B.11------AEYYYDSFHLDLRAAGTIAACFGLANVFARPMGGYLSDLAARYFGMRARLWNIWILQTAG 340

TaNRT2-6D.14------AEYYYDSFHLDLRAAGTIAACFGLANVFARPMGGYLSDLGARYFGMRARLWNIWILQTAG 340

TaNRT2-6A.12------AEYYYDSFHLDLRAAGTIAACFGLANVFARPMGGYLSDLGARYFGMRARLWNIWILQTAG 340

TaNRT2-6D.13------AEYYYDSFHLDLRAAGTIAACFGLANVFARPMGGYLSDLGARYFGMRARLWNIWILQTAG 340

TaNRT2-6D.11------AEYYYDSFHLDLRAAGTIAASFGLANIFARPMGGYLSDLGARYFGMRARLWNIWILQTAG 339

TaNRT2-6D.12------AEYYYDSFHLDLRAAGTIAASFGLANIFARPMGGYLSDLGARYFGMRARLWNVWILQTAG 341

TaNRT2-6B.10------AEYYYDSFHLDLRAAGTIAASFGLANIFARPMGGYLSDLGARYFGMRARLWNIWILQTAG 341

TaNRT2-6A.11------AEYYYDSFHLDLRAAGTIAASFGLANIFARPMGGYLSDLGARYFGMRARLWNIWILQTAG 341

TaNRT2-6D.10------AEYYYDSFHLDLRAAGTIAASFGLANIFARPMGGYLSDLGARYFGMRARLWNIWILQTAG 341

TaNRT2-6A.10------AEYYYDSFHLDLRAAGTIAASFGLANIFARPMGGYLSDLGARYFGMRARLWNIWILQTAG 341

TaNRT2-U.2--------AEYYYDSFHLDLRAAGTI------------------------------------------ 259

TaNRT2-6D.1-------AEYYFDHFHLDLRTSGTIAACFGMANLVARPMGGYLSDLGARYFGMRARLWNIWILQTAG 341

TaNRT2-6A.6-------AEYYFDHFHLDLRTSGTIAACFGMANLVARPMGGYLSDLGARYFGMRARLWNIWILQTAG 341

TaNRT2-6B.1-------AEYYFDHFHLDLRTSGTIAACFGMANIVARPVGGYLSDLGARYFGMRARLWNIWILQTAG 341

TaNRT2-6B.6-------AEYYYDHFHLDLRAAGTIAACFGMANIVARPMGGYLSDLGARYFGMHARLWNIWILQTTG 317

TaNRT2-6B.4-------AEYYYDHFHLDLRAAGTIAACFGMANIVARPMGGYLSDLGARYFGMRARLWNIWILQTAG 339

TaNRT2-6D.5-------AEYYYDHFHLDLRAAGTIAACFGMANIVARPMGGYLSDLGARYFGMRARLWNIWILQTAG 137

TaNRT2-6A.2-------AEYYYDHFHLDLRAAGTIAACFGMANIVARPMGGYLSDLGARYFGMRARLWNIWILQTAG 339

TaNRT2-6B.5-------AEYYYDHFHLDLRAAGTIAACFGMANIVARPMGGYLSDLGARYFGMRARLWNIWILQTAG 397

TaNRT2-6B.3-------AEYYYDHFHLDLRAAGTIAACFGMANIVARPMGGYLSDLGARYFGMRARLWNIWILQTAG 339

TaNRT2-6A.1-------AEYYYDHFHLDLRAAGTIAACFGMANIVARPMGGYLSDLGARYFGMRARLWNIWILQTAG 339

TaNRT2-6A.3-------AEYYYDHFHLDLRAAGTIAACFGMANIVARPMGGYLSDLGARYFGMRARLWNIWILQTAG 339

TaNRT2-6D.3-------AEYYYDHFHLDLRAAGTIAACFGMANIVARPMGGYLSDLGARYFGMRARLWNIWILQTAG 339

TaNRT2-6D.6-------AEYYYDHFHLDLRAAGTIAACFGMANIVARPMGGYLSDLGARYFGMRARLWNIWILQTAG 339

TaNRT2-6D.4-------AEYYYDHFHLDLRAAGTIAACFGMANIVARPMGGYLSDLGARYFGMRARLWNIWILQTAG 339

TaNRT2-6B.2-------AEYYYDHFHLDLRAAGTIAACFGMANIVARPMGGYLSDLGARYFGMRARLWNIWILQTAG 339

TaNRT2-6A.5-------AEYYYDHFHLDLRAAGTIAACFGMANIVARPMGGYLSDLGARYFGMRARLWNIWILQTAG 339

TaNRT2-6A.4-------AEYYYDHFHLDLRAAGTIAACFGMANIVARPMGGYLSDLGARYFGMRARLWNIWILQTAG 339

TaNRT2-6D.2-------AEYYYDHFHLDLRAAGTIAACFGMANIVARPMGGYLSDLGARYFGMRARLWNIWILQTAG 227

OsNRT2.1----------AEYFYDRFDLDLRVAGIIAASFGMANIVARPTGGLLSDLGARYFGMRARLWNIWILQTAG 363

OsNRT2.2----------AEYFYDRFDLDLRVAGIIAASFGMANIVARPTGGLLSDLGARYFGMRARLWNIWILQTAG 363

ZmNRT2.1----------AEYMYDRFDLDLRVAGTIAACFGMANIVARPMGGIMSDMGARYWGMRARLWNIWILQTAG 360

ZmNRT2.2----------AEYMYDRFDLDLRVAGTIAACFGMANIVARPMGGIMSDMGARYWGMRARLWNIWILQTAG 360

AtNRT2.6----------SGYFYDRFNLTLHTAGIIAASFGMANFFARPFGGYASDVAARLFGMRGRLWILWILQTVG 372

AtNRT2.3----------SGYFYDRFNLKLQTAGIVAASFGMANFIARPFGGYASDVAARVFGMRGRLWTLWIFQTVG 369

AtNRT2.4----------AEYFFDRFHLKLHTAGIIAASFGMANFFARPIGGWASDIAARRFGMRGRLWTLWIIQTLG 362

AtNRT2.1----------AEYFFDRFHLKLHTAGLIAACFGMANFFARPAGGYASDFAAKYFGMRGRLWTLWIIQTAG 362

AtNRT2.2----------AEYFFDRFHLKLHTAGIIAACFGMANFFARPAGGWASDIAAKRFGMRGRLWTLWIIQTSG 360

--------------------:----*-:-:--:*--------------------------------------------

OsNRT2.4----------AALCVLVGRMGAAEAPSLAATVAVMVACAAFVQAASGLTFGIVPFVCKRSLGVVSGMTAS 411

TaNRT2-7A---------AVLCVLVGRMGATEAPSLAATMAVMVACGAFVQAASGLTFGIVPFVSKRSMGVVSGMTAS 405

TaNRT2-7B---------AVLCVLVGRMGATEAPSLAATMAVMVACGAFVQAASGLTFGIVPFVSKRSMGVVSGMTAS 405

TaNRT2-7D---------AVLCVMVGRMGATEAPSLAATMAVMVACGAFVQAASGLTFGIVPFVSKRSMGVVSGMTAS 402

AtNRT2.7----------GLLCVLLGRVN-----SLWGSILVMWVFSVFVQAASGLVFGVVPFVSTRSLGVVAGITGS 406

AtNRT2.5----------GVLCACLGQIS-----SLTVSIIVMLVFSVFVQAACGLTFGVVPFISRRSLGVVSGMTGA 399

OsNRT2.3----------GVLCVVLGIVDF----SFAASVAVMVLFSFFVQAACGLTFGIVPFVSRRSLGLISGMTGG 371

ZmNRT2.5----------GVLCVVLGAVDY----SFAASVAVMILFSMFVQAACGLTFGIVPFVSRRSLGLISGMTGG 407

TaNRT2-3A---------GVLCVVLGVVDY----SFGASVAVMILFSFFVQAACGLTFGIVPFVSRRSLGLISGMTGG 399

TaNRT2-3B---------GILCVVLGVVDY----SFGASVAVMILFSFFVQAACGLTFGIVPFVSRRSLGLISGMTGG 399

TaNRT2-3D---------GILCVVLGVVDY----SFGASVAVMILFSFFVQAACGLTFGIVPFVSRRSLGLISGMTGG 399

TaNRT2-1D---------GVLCVVLGIVDY----SFGASVAVMILFSLFCQAACGLTFGIVPFVSRRSLGLISGMTGG 401

TaNRT2-U.1--------GVLCVVLGIVDY----SFGASVAVMILFSLFCQAACGLTFGIVPFVSRRSLGLISGMTGG 398

TaNRT2-2A---------GAFCFWLGRAS-----SLPASVTAMVLFSICAQAAEGAIFAVIPFVSRRSLGIVSGMTGA 394

TaNRT2-2D---------GAFCFWLGRAS-----SLPASVTAMVLFSICAQAAEGAIFAVIPFVSRRSLGIVSGMTGA 391

ZmNRT2.3----------GAFCFWLGRAS-----ELPASVTAMVLFSFCAQAACGATFGVIPFVSRRSLGVISGLTGA 410

TaNRT2-6B.7-------GVFCLCLGRAS-----TLPTSIACMVLYSICVEAACGAVYGVIPFVSRRSLGLVSGMTGA 395

TaNRT2-6A.7-------GVFCLCLGRAS-----TLPTSIACMVLYSICVEAACGAVYGVIPFVSRRSLGLVSGMTGA 395

TaNRT2-6D.7-------GVFCLCLGRAS-----TLPTSIACMVLYSICVEAACGAVYGVIPFVSRRSLGLVSGMTGA 395

TaNRT2-6D.8-------GVFCLCLGRAS-----TLPTSIACMVLYSICVEAACGAVYGVIPFVSRRSLGLVSGMTGA 395

TaNRT2-6A.8-------GVFCLCLGRAS-----TLPTSIVCMVLYSICVEAACGAVYGVIPFVSRRSLGLVSGMTGA 395

TaNRT2-6B.8-------GVFCLCLGRAS-----TLPTSIVCMVLYSICVEAACGAVYGVIPFVSRRSLGLVSGMTGA 393

TaNRT2-6A.9-------GAFCLCLGRAS-----ALPTSITCMVLYSICIEAACGAVYGVIPFVSRRSLGLISGMSGA 394

TaNRT2-6B.9-------GAFCLCLGRAS-----TLPTSITCMVLYSICVEAACGAVYGVIPFVSKRSLGLISGMSGA 394

TaNRT2-6D.9-------GAFCLCLGRAS-----TLPTSITCMVLYSICVEAACGAVYGVIPFVSRRSLGLISGMSGA 394

TaNRT2-6A.13------GAFCICLGRAS-----SLPTSITCMVLYSICVEAACGAVYGVIPFVSRRSLGLISGMSGA 395

TaNRT2-6B.11------GAFCICLGRAS-----TLPTSITCMVLYSICVEAACGAVYGVIPFVSRRSLGLISGMSGA 395

TaNRT2-6D.14------GAFCLCLGRAT-----TLPTSITCMVLYSICVEAACGAVYGVIPFVSRRSLGLISGMSGA 395

TaNRT2-6A.12------GAFCLCLGSAT-----TLPTSITCMVLYSICVEAACGAVYGVIPFVSRRSLGLISGMSGA 395

TaNRT2-6D.13------GAFCLCLGRAT-----TLPTSITCMVLYSICVEAACGAVYGVIPFVSRRSLGLISGMSGA 395

TaNRT2-6D.11------GVLCICLGRAS-----SLPTSVTCMVLYSTCVEDACGAVYGVIPFVSRRSLGLISGMSGA 394

TaNRT2-6D.12------GVFCICLGRAS-----SLPISVTFMVLYSISVEAACGAVYGVIPFISRRSLGLISGMSGA 396

TaNRT2-6B.10------GVFCICLGRAS-----SLPTSVTCMVLYSICVEAACGAVYGVIPFVSRRSLGLISGMSGA 396

TaNRT2-6A.11------GVFCICLGRAS-----SLPTSVTCMVLYSICVEAACGAVYGVIPFVSRRSLGLISGMSGA 396

TaNRT2-6D.10------GVFCICLGRAS-----SLPTSVTCMVLYSICVEAACGAVYGVIPFVSRRSLGLISGMSGA 396

TaNRT2-6A.10------GVFCICLGRAS-----SLPTSVTCMVLYSICVEAACGAVYGVIPFVSRRSLGLISGMSGA 396

TaNRT2-U.2-------------------------------------------------------------------- 259

TaNRT2-6D.1-------GAFCLWLGRAK-----ALPESITAMVLFSICAQAACGAIFGVIPFVSRRSLGIISGLSGA 396

TaNRT2-6A.6-------GAFCLWLGRAK-----ALPESVTAMVLFSVCAQAACGAVFGVIPFVSRRSLGIISGMSGA 396

TaNRT2-6B.1-------GAFCLWLGRAK-----ALPESITAMVLFSICAQAACGAVFGVIPFVSRRSLGIISGLSGA 396

TaNRT2-6B.6-------GAFCIWLGRAS-----ALPASVTAMVLFSICAQAACGAVFRVAPFVSRRSLGIISALTGA 372

TaNRT2-6B.4-------GAFCIWLGRAS-----ALPASVTAMVLFSICAQAACGAVFGVAPFVSRRSLGIISGLTGA 394

TaNRT2-6D.5-------GAFCIWLGRAL-----ALPASVTAMVLFSICAQAACGAVFGVAPFVSRRSLGIISGLTGA 192

TaNRT2-6A.2-------GAFCIWLGRAS-----ALPASVTAMVLFSICAQAACGAVFGVAPFVSRRSLGIISGLTGA 394

TaNRT2-6B.5-------GAFCIWLGRAS-----ALPASVTAMVLFSICAQAACGAVFGVAPFVSRRSLGIISGLTGA 452

TaNRT2-6B.3-------GAFCIWLGRAS-----ALPASVTAMVLFSICAQAACGAVFGVAPFVSRRSLGIISGLTGA 394

TaNRT2-6A.1-------GAFCIWLGRAS-----ALPASVTAMVLFSICAQAACGAVFGVAPFVSRRSLGIISGLTGA 394

TaNRT2-6A.3-------GAFCIWLGRAS-----ALPASVTAMVLFSICAQAACGAVFGVAPFVSRRSLGIISGLTGA 394

TaNRT2-6D.3-------GAFCIWLGRAS-----ALPASVTAMVLFSICAQAACGAVFGVAPFVSRRSLGIISGLTGA 394

TaNRT2-6D.6-------GAFCIWLGRAS-----ALPASVTAMVLFSICAQAACGAVFGVAPFVSRRSLGIISGLTGA 394

TaNRT2-6D.4-------GAFCIWLGRAS-----ALPASVTAMVLFSICAQAACGAVFGVAPFVSRRSLGIISGLTGA 394

TaNRT2-6B.2-------GAFCIWLGRAS-----ALPASVTAMVLFSICAQAACGAVFGVAPFVSRRSLGIISGLTGA 394

TaNRT2-6A.5-------GAFCIWLGRAS-----ALPASVTAMVLFSICAQAACGAVFGVAPFVSRRSLGIISGLTGA 394

TaNRT2-6A.4-------GAFCIWLGRAS-----ALPASVTAMVLFSICAQAACGAVFGVAPFVSRRSLGIISGLTGA 394

TaNRT2-6D.2-------GAFCIWLGRAS-----ALPASVTAMVLFSICAQAACGAVFGVAPFVSRRSLGIISGLTGA 282

OsNRT2.1----------GAFCLLLGRAS-----TLPTSVVCMVLFSFCAQAACGAIFGVIPFVSRRSLGIISGMTGA 418

OsNRT2.2----------GAFCLLLGRAS-----TLPTSVVCMVLFSFCAQAACGAIFGVIPFVSRRSLGIISGMTGA 418

ZmNRT2.1----------GAFCLWLGRAS-----TLPVSVVAMVLFSFCAQAACGAIFGVIPFVSRRSLGIISGMTGA 415

ZmNRT2.2----------GAFCLWLGRAS-----TLPVSVVAMVLFSFCAQAACGAIFGVIPFVSRRSLGIISGMTGA 415

AtNRT2.6----------ALFCIWLGRAS-----SLPIAILAMMLFSMGTQAACGALFGVAPFVSRRSLGLISGLTGA 427

AtNRT2.3----------ALFCIWLGRAS-----SLPIAILAMMLFSIGTQAACGALFGVAPFVSRRSLGLISGLTGA 424

AtNRT2.4----------GFFCLWLGRAT-----TLPTAVVFMILFSLGAQAACGATFAIIPFISRRSLGIISGLTGA 417

AtNRT2.1----------GLFCVWLGRAN-----TLVTAVVAMVLFSMGAQAACGATFAIVPFVSRRALGIISGLTGA 417

AtNRT2.2----------GLFCVWLGRAN-----TLVTAVVSMVLFSLGAQAACGATFAIVPFVSRRALGIISGLTGA 415

------------------------------------------------------------------------------

OsNRT2.4----------GGAVGAIVTNRLFFSGSRYTVEEAISCTGITSLLC--------TLPVALIHFRRQGGMFC 463

TaNRT2-7A---------GRRGCRRDRDKPV-------VLQQLQVHGGGGHLIHWPHQPPLHAPCGAHLFPTLGRDAL 458

TaNRT2-7B---------GGAVGAIVTNRLFFSSSRYTVEEAISFTGLTSLLC--------TLPVALIYFPRLGGMLC 457

TaNRT2-7D---------GGAVGAIVTNRLFFSSSRYTVEEAISFTGLTSLLC--------TLPVALIYFPRLGGMLC 454

AtNRT2.7----------GGTVGAVVTQFLLFSGDDVRKQRSISLMGLMTFVF--------ALSVTSIYFPQWGGMCC 458

AtNRT2.5----------GGNVGAVLTQLIFFKGSTYTRETGITLMGVMSIAC--------SLPICLIYFPQWGGMFC 451

OsNRT2.3----------GGNVGAVLTQYIFFHGTKYKTETGIKYMGLMIIAC--------TLPVMLIYFPQWGGMLV 423

ZmNRT2.5----------GGNVGAVLTQLIFFHGSKYKTETGIKYMGFMIIAC--------TLPITLIYFPQWGGMFL 459

TaNRT2-3A---------GGNVGAVLTQVIFFRGTKYKTETGIMYMGLMILAC--------TLPITLIYFPQWGGMFA 451

TaNRT2-3B---------GGNVGAVLTQVIFFRGTTYKTETGIMYMGLMILAC--------TLPITLIYFPQWGGMFA 451

TaNRT2-3D---------GGNVGAVLTQVIFFRGTTYKTETGIMYMGLMILAC--------TLPITLIYFPQWGGMFA 451

TaNRT2-1D---------GGNVGAVLTQVIFFRGGKYKTETGIMYMGIMILAC--------TLPVAFIYFPQWGGMLA 453

TaNRT2-U.1--------GGNVGAVLTQVIFFRGGKYKTETGIMYMGIMILAC--------TLPVAFIYFPQWGGMLA 450

TaNRT2-2A---------GGTFGAAFNQLLFFTSSNYGTGQGLQYMGIVTMAC--------TLPVMLVHFPQWGSMLF 446

TaNRT2-2D---------GGTLGAAFNQLLFFTSSKYGTGQGLQYMGIVTMAC--------TLPVMLVHFPQWGSMLF 443

ZmNRT2.3----------GGNVGAGLTQLLFFTTSSYSTRKGIENMGIMAMAC--------TLPLVLVHFPQWGSMLL 462

TaNRT2-6B.7-------GGNVGGGLTQLLFFTSSQYTTSKGLQYMGIMIMAC--------TLPVILVHFPQWGSMLV 447

TaNRT2-6A.7-------GGNVGGGLTQLLFFTSSQYTTGKGLQYMGIMIMAC--------TLPVILVHFPQWGSMLV 447

TaNRT2-6D.7-------GGNVGGGLTQLLFFTSSQYTTGKGLQYMGIMIMAC--------TLPVILVHFPQWGSMLV 447

TaNRT2-6D.8-------GGNVGGGLTQLLFFTSSQYTTGKGLQYMGIMIMAC--------TLPVILVHFPQWGSMLV 447

TaNRT2-6A.8-------GGNVGGGLTQLLFFTSSQYTTGKGLQYMGIMIIAC--------TLPVVLVHFPQWGSMLV 447

TaNRT2-6B.8-------GGNVGGGLTQLLFFTSSQYTTGKGLQYMGIMIIAC--------TLPVVLVHFPQWGSMLV 445

TaNRT2-6A.9-------GGNVGGGLTQFLFFTSSQYSTGKGLQYMGIMVMAC--------TLPVALVHFPQWGSMLL 446

TaNRT2-6B.9-------GGNVGGGLTQFLFFTSSQYSTGKGLQYMGIMVMAC--------TLPVALVHFPQWGSMLL 446

TaNRT2-6D.9-------GGNVGGGLTQFLFFTSSQYSTGKGLQYMGIMVMAC--------TLPVALIHFPQWGSMLL 446

TaNRT2-6A.13------GGNVGGGLTQFLFFTSSQYSTGKGLQYMGIMVMAC--------TLPVALVHFPQWGSMLL 447

TaNRT2-6B.11------GGNVGGGLTQFLFFTSSQYSTGKGLQYMGIMVMAC--------TLPVALVHFPQWGSMLL 447

TaNRT2-6D.14------GGNVGGGLTQFLFFTSSQYSTGKGLQYMGIMVMAC--------TLPVALVHFPQWGSMLL 447

TaNRT2-6A.12------GGNVGGGLTQFLFFTSSQYSTGKGLQYMGIMVMAC--------TLPVALVHFPQWGSMLL 447

TaNRT2-6D.13------GGNVGGGLTQFLFFTSSQYSTGKGLQYMGIMVMAC--------TLPVALVHFPQWGSMLL 447

TaNRT2-6D.11------GGNVGGGLTQFLFFTSSQYTTSKGLQYMGIMIMVC--------TLPVALVHFPQWGSMLL 446

TaNRT2-6D.12------GGNVGGGLTQFLFFTSSQYTTGKGLQYMGIMIIVC--------TLPVTLVHFPQWGSMLL 448

TaNRT2-6B.10------GGNVGGGLTQFLFFTSSQYTTGKGLQYMGIMIMVC--------TLPIALVHFPQWGSMLL 448

TaNRT2-6A.11------GGNVGGGLTQFLFFTSSQYSTGKGLQYMGIMIMVC--------TLPITLVHFPQWGSMLL 448

TaNRT2-6D.10------GGNVGGGLTQFLFFTSSQYTTGKGLQYMGIMIMVC--------TLPVALVHFPQWGSMLL 448

TaNRT2-6A.10------GGNVGGGLTQFLFFTSSQYTTGKGLQYMGIMIMVC--------TLPVALVHFPQWGSMLL 448

TaNRT2-U.2-------------------------------------------------------------------- 259

TaNRT2-6D.1-------GGNFGAGLTQLLFFTSSKYGTGMGLEYMGIMIMAC--------TLPVVLVHFPQWGSMLL 448

TaNRT2-6A.6-------GGNFGAGLTQLLFFTSSKYGTGRGLEYMGIMIMAC--------TLPVALVHFPQWGSMLL 448

TaNRT2-6B.1-------GGNFGAGLTQLLFFTSSKYGTGRGLEYMGIMIMAC--------TLPVALVHFPQWGSMLL 448

TaNRT2-6B.6-------GGNVGAGLTQLLFFTSSQYSTGRGLEYMGIMIMAC--------TLPVALVHFPQWGSMFF 424

TaNRT2-6B.4-------GGNVGAGLTQLLFFTSSQYSTGRGLEYMGIMIMAC--------TLPVALVHFPQWGSMFF 446

TaNRT2-6D.5-------GGNVGAGLTQLLFFTSSQYSTGRGLEYMGIMIMAC--------TLPVALVHFPQWGSMFF 244

TaNRT2-6A.2-------GGNVGAGLTQLLFFTSSQYSTGRGLEYMGIMIMAC--------TLPVALVHFPQWGSMFF 446

TaNRT2-6B.5-------GGNVGAGLTQLLFFTSSQYSTGRGLEYMGIMIMAC--------TLPVALVHFPQWGSMFF 504

TaNRT2-6B.3-------GGNVGAGLTQLLFFTSSQYSTGRGLEYMGIMIMAC--------TLPVTLVHFPQWGSMFF 446

TaNRT2-6A.1-------GGNVGAGLTQLLFFTSSQYSTGRGLEYMGIMIMAC--------TLPVALVHFPQWGSMFF 446

TaNRT2-6A.3-------GGNVGAGLTQLLFFTSSQYSTGRGLEYMGIMIMAC--------TLPITLVHFPQWGSMFF 446

TaNRT2-6D.3-------GGNVGAGLTQLLFFTSSQYSTGRGLEYMGIMIMAC--------TLPVALVHFPQWGSMFF 446

TaNRT2-6D.6-------GGNVGAGLTQLLFFTSSQYSTGRGLEYMGIMIMAC--------TLPVALVHFPQWGSMFF 446

TaNRT2-6D.4-------GGNVGAGLTQLLFFTSSQYSTGRGLEYMGIMIMPC--------TLPVALVHFPQWGSMFF 446

TaNRT2-6B.2-------GGNVGAGLTQLLFFTSSQYSTGRGLEYMGIMIMAC--------TLPVALVHFPQWGSMFF 446

TaNRT2-6A.5-------GGNVGAGLTQLLFFTSSQYSTGRGLEYMGIMIMAC--------TLPVALVHFPQWGSMFF 446

TaNRT2-6A.4-------GGNVGAGLTQLLFFTSSQYSTGRGLEYMGIMIMAC--------TLPVTLVHFPQWGSMFF 446

TaNRT2-6D.2-------GGNVGAGLTQLLFFTSSQYSTGRGLEYMGIMIMAC--------TLPVALVHFPQWGSMFF 334

OsNRT2.1----------GGNFGAGLTQLLFFTSSRYSTGTGLEYMGIMIMAC--------TLPVVLVHFPQWGSMFL 470

OsNRT2.2----------GGNFGAGLTQLLFFTSSRYSTGTGLEYMGIMIMAC--------TLPVVLVHFPQWGSMFL 470

ZmNRT2.1----------GGNFGAGLTQLLFFTSSTYSTGRGLEYMGIMIMAC--------TLPVVFVHFPQWGSMFF 467

ZmNRT2.2----------GGNFGAGLTQLLFFTSSTYSTGRGLEYMGIMIMAC--------TLPVVFVHFPQWGSMFF 467

AtNRT2.6----------GGNFGSGVTQLLFFSSSRFSTAEGLSLMGVMAVVC--------SLPVAFIHFPQWGSMFL 479

AtNRT2.3----------GGNFGSGLTQLLFFSSARFSTAEGLSLMGVMAVLC--------TLPVAFIHFPQWGSMFL 476

AtNRT2.4----------GGNFGSGLTQLVFFSTSTFSTEQGLTWMGVMIMAC--------TLPVTLVHFPQWGSMFL 469

AtNRT2.1----------GGNFGSGLTQLLFFSTSHFTTEQGLTWMGVMIVAC--------TLPVTLVHFPQWGSMFL 469

AtNRT2.2----------GGNFGSGLTQLVFFSTSRFTTEEGLTWMGVMIVAC--------TLPVTLIHFPQWGSMFF 467

------------------------------------------------------------------------------

OsNRT2.4----------GPSATID-------GDGDVDDDDDYMLLK------------------------------- 485

TaNRT2-7A---------RPLGNRHRR--------P------------------------------------------ 468

TaNRT2-7B---------GPSESATVDHDGHDDDDDVNKDDDYMLLK------------------------------- 486

TaNRT2-7D---------GPSESDTVDHDGHDDDDDVNKDDDYMLLK------------------------------- 483

AtNRT2.7----------GPSSS---SEEEDISRGLLVEDEDEEGKVVSGSLRPVC---------------------- 493

AtNRT2.5----------GPSSK--KVTEEDYYLAEWNDEEKEKNLHI-GSQKFAETSISERGRATTTHPQT------ 502

OsNRT2.3----------GPRKG---ATAEEYYSREWSDHEREKGFNA-ASVRFAENSVREGGRSSANGGQPRHTVPV 479

ZmNRT2.5----------GPRPG---ATAEDYYNREWTAHECDKGFNT-ASVRFAENSVREGGRSGSQS--KHTTVPV 513

TaNRT2-3A---------GPRKG---ATAEEYYSQEWTEEERAKGYSA-ATERFAENSVREGGRRAASGSQSRHTVPV 507

TaNRT2-3B---------GPRKG---ATAEEYYSQEWTEEERAKGYSA-ATERFAENSVREGGRRAASGSQSRHTVPV 507

TaNRT2-3D---------GPRKG---ATAEEYYSQEWTEEERAKGYSA-ATERFAENSVREGGRRATSGSQSRHTVPV 507

TaNRT2-1D---------GPRPG---ATADDYYNGEWTAEERDKGYNA-ATKRFAENSVREGGRRAASGSDSRHTVPV 509

TaNRT2-U.1--------GPRKG---ATADDYYNGEWTAEEREKGYNA-ATKRFAENSVREGGRRAASGSRSRHTVPV 506

TaNRT2-2A---------PANVG---ADEEKYYGAEWSEEEKSKGLNA-RTVKFAQNCRSERGRHRNVILANDT---- 498

TaNRT2-2D---------PANVG---ADEDKYYGAEWSEEEKSKGLNA-RTVKFAQNCRSERGRHRNAILANDT---- 495

ZmNRT2.3----------PPSAD---ADEERYYASEWSEDEKSVGRHS-ASLKFAENSRSERGKRNAVAVLATAAAT- 517

TaNRT2-6B.7-------PPSPD---ATEEEYYAAEWTEEEKGKGLHM-AGLKFAENSISERGRRNAILAVP--ATPP 501

TaNRT2-6A.7-------PPSAD---ATEEEYYAAEWTEEEKSKGLHM-AGLKFAENSISERGKRNAILAVP--ATPP 501

TaNRT2-6D.7-------PPSMD---ATEEEYYAAEWTEEEKGKGLHM-AGLKFAENSISERGRRNAILAVP--ATPP 501

TaNRT2-6D.8-------PPSAD---ATEEEYYAAEWTEEEKGKGLHM-AGLKFAENSISERGRRNAILAVP--ATPP 501

TaNRT2-6A.8-------PPSAD---ATEEEYYSTEWTEEEKGKGLHM-AGLKFAENSISERGRRNAILAVP--ATPP 501

TaNRT2-6B.8-------PPSAD---ATEGEYYSSEWTEEEKGKGLHM-AGLKFAENSISERGRRNAILAVP--ATPP 499

TaNRT2-6A.9-------PPSAN---ATEEDYYGAEWTEEEKNKGLHI-TSLKFAENSISERGRRNAILAAP--VTPP 500

TaNRT2-6B.9-------PPSAD---ATEEDYYGAEWTEEEKNKGLHI-ASLKFAENSISERGRRNAILAAP--ATPP 500

TaNRT2-6D.9-------PPSAD---ATEEDYYGAEWTEEEKNKGLHI-ASLKFAENSISERGRRNAILAAP--TTPP 500

TaNRT2-6A.13------PPTAS---ATEEDYYAAEWTEEERSKGLHN-AGIKFAENSVSERGRRNAILAVP--ASPP 501

TaNRT2-6B.11------PPTEG---ATEEDYYAAEWTEEEKSKGLHN-AGIKFAENSVSERGRRNAILAVP--ASPP 501

TaNRT2-6D.14------PPTAG---ATEEEYYAAEWTEEEKSKGLHN-AGIKFAENSVSERGRRNAILAVP--ATPS 501

TaNRT2-6A.12------PPTTG---ATEEEYYAAEWTEEEKSKGLHN-AGIKFAENSVSERGRRNTILAVP--ATPP 501

TaNRT2-6D.13------PPTAG---ATEEDYYAAEWTEEEKSKGLHN-AGIKFAENSVSERGRRNAILAVP--GTPP 501

TaNRT2-6D.11------PPSTD---ATEEEYYGAKWTEEEKSKGLHI-AGQKFAENSISERGKRNAILAVP--SSQP 500

TaNRT2-6D.12------PPRAD---ATEEEYYGAEWTEEEKSKGLHL-AGVKFAENSVSERGKRNAILAVP--SSPP 502

TaNRT2-6B.10------PPSAD---ATEEDYYSAEWTEEEKSKGLHL-GGLKFAENSVSERGKRNAILAVP--SSPP 502

TaNRT2-6A.11------PPSAD---AAEEDYYGAEWTEEEKSKGLHL-AGLKFAENSVSERGKRNAILAVP--CSPP 502

TaNRT2-6D.10------PPSAD---ATEEEYYGAEWTEEEKSKGLHI-AGQKFAENSISERGKRNAILAVP--SSPP 502

TaNRT2-6A.10------PPGAD---ATEEEYYGAEWTEEEKSKGLHI-AGQKFAENSISERGKRNAILAVP--SSPP 502

TaNRT2-U.2-------------------------------------------------------------------- 259

TaNRT2-6D.1-------PPNAN---ATEEDFYAAEWSEEEKKKGLHI-PGQKFAENSRSERGRRNVILATA--ATPP 502

TaNRT2-6A.6-------PPNAS---ATEEDFYAAEWSEEEKKKGLHI-PGQKFAENSRSERGRRNVILATA--ATPP 502

TaNRT2-6B.1-------PPNAD---ATEEDFYAAEWSEEEKKKGLHI-PGQKFAENSRSERGRRNVILATA--ATPP 502

TaNRT2-6B.6-------PASAD---ATEEEYYASEWSEEEKSKGLHI-AGQKFAENSRSERGRRNVILATS--ATPP 478

TaNRT2-6B.4-------PASAD---ATEEEYYASEWSEEEKGKGLHI-TGQKFAENSRSERGRRNVILATS--ATPP 500

TaNRT2-6D.5-------PASAD---ATEEEYYASEWSEEEKSKGLHI-AGQKFAENSRSERGRRNVVLATS--ATPP 298

TaNRT2-6A.2-------PASAD---ATEEEYYASEWSEEEKSKGLHI-AGQKFAENSRSERGRRNVVLATS--AMPP 500

TaNRT2-6B.5-------PASAD---ATEEEYYASEWSEEEKSKGLHI-AGQKFAENSRSERGRRNVVLATS--ATPP 558

TaNRT2-6B.3-------PASAD---ATEEEYYGSEWSEEEKRNGLHI-AGQKFAENSRSERGRRNVILATS--ATPP 500

TaNRT2-6A.1-------PASAD---ATEEEYYASEWSEEEKSKGLHI-AGQKFAENSRSERGRRNVILAAS--ATPP 500

TaNRT2-6A.3-------PASAD---ATEEEYYASEWSEEEKGKGLHI-AGQKFAENSRSERGRRNVILATS--ATPP 500

TaNRT2-6D.3-------PASAD---ATEEEYYGSEWSEEEKRNGLHI-AGQKFAENSRSERGRRNVILATS--ATPP 500

TaNRT2-6D.6-------PASTD---ATEEEYYASEWSEEEKSKGLHI-AGQKFAENSRSERGRRNVILATS--ATPP 500

TaNRT2-6D.4-------PASAD---ATEEEYYASEWSEEEKGKGLHI-AGQKFAENSRSERGRRNVILATS--ATPP 500

TaNRT2-6B.2-------PASAD---ATEEEYYASEWSEEEKNKGLHI-AGQKFAENSRSERGRRNVILATS--VTPP 500

TaNRT2-6A.5-------PASAD---ATEEEYYASEWSEEEKNKGLHI-AGQKFAENSRSERGRRNVILATS--ATPP 500

TaNRT2-6A.4-------PASAD---ATEEEYYASEWSEEEKNKGLHI-AGQKFAENSRSERGRRNVILATS--ATPP 500

TaNRT2-6D.2-------PASAD---ATEEEYYASEWSEEEKNKGLHI-AGQKFAENSRSERGRSNVILATS--ATPP 388

OsNRT2.1----------PPNAG---AEEEHYYGSEWSEQEKSKGLHG-ASLKFAENSRSERGRRNVINAAAAAATPP 526

OsNRT2.2----------PPNAG---AEEEHYYGSEWSEQEKSKGLHG-ASLKFAENSRSERGRRNVINAAAAAATPP 526

ZmNRT2.1----------PPSAT---ADEEGYYASEWNDDEKSKGLHS-ASLKFAENSRSERGKRNVIQADAAATPE- 522

ZmNRT2.2----------PPSAT---ADEEGYYASEWNDDEKSKGLHS-ASLKFAENSRSERGKRNVIQADAAATPE- 522

AtNRT2.6----------RPSQDGEKSKEEHYYGAEWTEEEKSLGLHE-GSIKFAENSRSERGRKAMLADIP---TPE 535

AtNRT2.3----------RPSTDGERSQEEYYYGSEWTENEKQQGLHE-GSIKFAENSRSERGRKVALANIP---TPE 532

AtNRT2.4----------PSTEDEVKSTEEYYYMKEWTETEKRKGMHE-GSLKFAVNSRSERGRRVASAPSP---PPE 525

AtNRT2.1----------PPSTDPVKGTEAHYYGSEWNEQEKQKNMHQ-GSLRFAENAKSEGGRRVRSAATP---PEN 525

AtNRT2.2----------PPSNDSVDATE-HYYVGEYSKEEQQIGMHL-KSKLFADGAKTEGGSSVHKGNAT---NNA 522

------------------------------------------------------------------------------

OsNRT2.4------------------- 485

TaNRT2-7A------------------ 468

TaNRT2-7B------------------ 486

TaNRT2-7D------------------ 483

AtNRT2.7------------------- 493

AtNRT2.5------------------- 502

OsNRT2.3----------DASPAGV-- 486

ZmNRT2.5----------ESSPADV-- 520

TaNRT2-3A---------DGSPADV-- 514

TaNRT2-3B---------DGSPADV-- 514

TaNRT2-3D---------DGSPADV-- 514

TaNRT2-1D---------DGSPAPADV 518

TaNRT2-U.1--------DSSPAPADV 515

TaNRT2-2A------------NQHA-- 502

TaNRT2-2D------------NQHA-- 499

ZmNRT2.3-------------PEHVV- 522

TaNRT2-6B.7-------NSTPQHV-- 508

TaNRT2-6A.7-------NSTPQHV-- 508

TaNRT2-6D.7-------NSTPQHV-- 508

TaNRT2-6D.8-------NSTPQHV-- 508

TaNRT2-6A.8-------NSTPQHV-- 508

TaNRT2-6B.8-------NSTPQHV-- 506

TaNRT2-6A.9-------NNTPQHV-- 507

TaNRT2-6B.9-------NNTPQHI-- 507

TaNRT2-6D.9-------NNTPQHV-- 507

TaNRT2-6A.13------HDTPQHV-- 508

TaNRT2-6B.11------HVTPQHV-- 508

TaNRT2-6D.14------HDTPQQV-- 508

TaNRT2-6A.12------HVTPQHV-- 508

TaNRT2-6D.13------HVTPQHV-- 508

TaNRT2-6D.11------NNTPQHV-- 507

TaNRT2-6D.12------NNTPQHV-- 509

TaNRT2-6B.10------NNTPQHV-- 509

TaNRT2-6A.11------NNTPQHV-- 509

TaNRT2-6D.10------NSTPLHV-- 509

TaNRT2-6A.10------NNTPQHV-- 509

TaNRT2-U.2----------------- 259

TaNRT2-6D.1-------NNTPQHA-- 509

TaNRT2-6A.6-------NNTPQHA-- 509

TaNRT2-6B.1-------NNTPQHA-- 509

TaNRT2-6B.6-------NNTPQHV-- 485

TaNRT2-6B.4-------NNTPQHV-- 507

TaNRT2-6D.5-------NNTPQHV-- 305

TaNRT2-6A.2-------NNTPQHV-- 507

TaNRT2-6B.5-------NNTPQHV-- 565

TaNRT2-6B.3-------NNTPQHV-- 507

TaNRT2-6A.1-------NNTPQHL-- 507

TaNRT2-6A.3-------NNTPQHV-- 507

TaNRT2-6D.3-------NNTPQHV-- 507

TaNRT2-6D.6-------NNTPQHV-- 507

TaNRT2-6D.4-------NNTPQHV-- 507

TaNRT2-6B.2-------NNTPQHV-- 507

TaNRT2-6A.5-------NNTPQHV-- 507

TaNRT2-6A.4-------NNTPQHV-- 507

TaNRT2-6D.2-------NNTPQHV-- 395

OsNRT2.1----------NNSPEHA-- 533

OsNRT2.2----------NNSPEHA-- 533

ZmNRT2.1---------------HV-- 524

ZmNRT2.2---------------HV-- 524

AtNRT2.6----------TGSPAHV-- 542

AtNRT2.3----------NGTPSHV-- 539

AtNRT2.4----------HV------- 527

AtNRT2.1----------TPNN--V-- 530

AtNRT2.2------------------- 522
