# Supplementary material for: Genome-wide systematic characterization of the NRT2 gene family and its expression profile in wheat (Triticum aestivum L.) during plant growth and in response to nitrate deficiency
Source: BMC Plant Biol. 2023 Jul 7;23:353. doi: 10.1186/s12870-023-04333-5 (PMC10327373; doi:10.1186/s12870-023-04333-5)
Supplement: Supplementary file 3 — Additional file 3. The amino acid sequence of NRT2s in Arabidopsis, maize, rice and wheat. [file 12870_2023_4333_MOESM3_ESM.docx]

**Additional file 3.** The amino acid sequence of NRT2s in *Arabidopsis*, maize, rice and wheat.

>AtNRT2.4

MADGFGEPGSSMHGVTGREQSYAFSVESPAVPSDSSAKFSLPVDTEHKAKVFKLLSFEAP

HMRTFHLAWISFFTCFISTFAAAPLVPIIRDNLNLTRQDVGNAGVASVSGSIFSRLVMGA

VCDLLGPRYGCAFLVMLSAPTVFSMSFVGGAGGYITVRFMIGFCLATFVSCQYWMSTMFN

GQIIGLVNGTAAGWGNMGGGVTQLLMPMVYEIIRRLGSTSFTAWRMAFFVPGWMHIIMGI

LVLTLGQDLPDGNRSTLEKKGAVTKDKFSKVLWYAITNYRTWVFVLLYGYSMGVELTTDN

VIAEYFFDRFHLKLHTAGIIAASFGMANFFARPIGGWASDIAARRFGMRGRLWTLWIIQT

LGGFFCLWLGRATTLPTAVVFMILFSLGAQAACGATFAIIPFISRRSLGIISGLTGAGGN

FGSGLTQLVFFSTSTFSTEQGLTWMGVMIMACTLPVTLVHFPQWGSMFLPSTEDEVKSTE

EYYYMKEWTETEKRKGMHEGSLKFAVNSRSERGRRVASAPSPPPEHV

>AtNRT2.6

MAHNHSNEDGSIGTSLHGVTAREQVFSFSVQEDVPSSQAVRTNDPTAKFALPVDSEHRAK

VFKPLSFAKPHMRAFHLGWISFFTCFISTFAAAPLVPVIRDNLDLTKTDIGNAGVASVSG

AIFSRLAMGAVCDLLGARYGTAFSLMLTAPAVFSMSFVADAGSYLAVRFMIGFCLATFVS

CQYWTSVMFTGKIIGLVNGCAGGWGDMGGGVTQLLMPMVFHVIKLTGATPFTAWRFAFFI

PGILQIVMGILVLTLGQDLPDGNLSTLQKSGQVSKDKFSKVFWFAVKNYRTWILFMLYGF

SMGVELTINNVISGYFYDRFNLTLHTAGIIAASFGMANFFARPFGGYASDVAARLFGMRG

RLWILWILQTVGALFCIWLGRASSLPIAILAMMLFSMGTQAACGALFGVAPFVSRRSLGL

ISGLTGAGGNFGSGVTQLLFFSSSRFSTAEGLSLMGVMAVVCSLPVAFIHFPQWGSMFLR

PSQDGEKSKEEHYYGAEWTEEEKSLGLHEGSIKFAENSRSERGRKAMLADIPTPETGSPA

HV

>AtNRT2.5

MEVEGKGGEAGTTTTTAPRRFALPVDAENKATTFRLFSVAKPHMRAFHLSWFQFFCCFVS

TFAAPPLLPVIRENLNLTATDIGNAGIASVSGAVFARIVMGTACDLFGPRLASAALTLST

APAVYFTAGIKSPIGFIMVRFFAGFSLATFVSTQFWMSSMFSGPVVGSANGIAAGWGNLG

GGATQLIMPIVFSLIRNMGATKFTAWRIAFFIPGLFQTLSAFAVLLFGQDLPDGDYWAMH

KSGEREKDDVGKVISNGIKNYRGWITALAYGYCFGVELTIDNIIAEYFFDRFHLKLQTAG

IIAASFGLANFFARPGGGIFSDFMSRRFGMRGRLWAWWIVQTSGGVLCACLGQISSLTVS

IIVMLVFSVFVQAACGLTFGVVPFISRRSLGVVSGMTGAGGNVGAVLTQLIFFKGSTYTR

ETGITLMGVMSIACSLPICLIYFPQWGGMFCGPSSKKVTEEDYYLAEWNDEEKEKNLHIG

SQKFAETSISERGRATTTHPQT

>AtNRT2.7

MEPSQRNTKPPSFSDSTIPVDSDGRATVFRPFSLSSPHSRAFHLAWLSLFSCFFSTFSIP

PLVPVISSDLNLSASTVSAAGIASFAGSIFSRLAMGPLCDLIGPRTSSAILSFLTAPVIL

SASLVSSPTSFILVRFFVGFSLANFVANQYWMSSMFSGNVIGLANGVSAGWANVGAGISQ

LLMPLIYSTIAEFLPRAVAWRVSFVFPAIFQVTTAVLVLLYGQDTPHGNRKNSNQNKLTI

PEEEEVLVVEEDERSSFVEILIGGLGNYRAWILALLYGYSYGVELTTDNVIAGYFYERFG

VNLEAAGTIAASFGISNIASRPAGGMISDALGKRFGMRGRLWGLWIVQSVAGLLCVLLGR

VNSLWGSILVMWVFSVFVQAASGLVFGVVPFVSTRSLGVVAGITGSGGTVGAVVTQFLLF

SGDDVRKQRSISLMGLMTFVFALSVTSIYFPQWGGMCCGPSSSSEEEDISRGLLVEDEDE

EGKVVSGSLRPVC

>AtNRT2.3

MTHNHSNEEGSIGTSLHGVTAREQVFSFSVDASSQTVQSDDPTAKFALPVDSEHRAKVFN

PLSFAKPHMRAFHLGWLSFFTCFISTFAAAPLVPIIRDNLDLTKTDIGNAGVASVSGAIF

SRLAMGAVCDLLGARYGTAFSLMLTAPTVFSMSFVGGPSGYLGVRFMIGFCLATFVSCQY

WTSVMFNGKIIGLVNGCAGGWGDMGGGVTQLLMPMVFHVIKLAGATPFMAWRIAFFVPGF

LQVVMGILVLSLGQDLPDGNLSTLQKSGQVSKDKFSKVFWFAVKNYRTWILFVLYGSSMG

IELTINNVISGYFYDRFNLKLQTAGIVAASFGMANFIARPFGGYASDVAARVFGMRGRLW

TLWIFQTVGALFCIWLGRASSLPIAILAMMLFSIGTQAACGALFGVAPFVSRRSLGLISG

LTGAGGNFGSGLTQLLFFSSARFSTAEGLSLMGVMAVLCTLPVAFIHFPQWGSMFLRPST

DGERSQEEYYYGSEWTENEKQQGLHEGSIKFAENSRSERGRKVALANIPTPENGTPSHV

>AtNRT2.1

MGDSTGEPGSSMHGVTGREQSFAFSVQSPIVHTDKTAKFDLPVDTEHKATVFKLFSFAKP

HMRTFHLSWISFSTCFVSTFAAAPLVPIIRENLNLTKQDIGNAGVASVSGSIFSRLVMGA

VCDLLGPRYGCAFLVMLSAPTVFSMSFVSDAAGFITVRFMIGFCLATFVSCQYWMSTMFN

SQIIGLVNGTAAGWGNMGGGITQLLMPIVYEIIRRCGSTAFTAWRIAFFVPGWLHIIMGI

LVLNLGQDLPDGNRATLEKAGEVAKDKFGKILWYAVTNYRTWIFVLLYGYSMGVELSTDN

VIAEYFFDRFHLKLHTAGLIAACFGMANFFARPAGGYASDFAAKYFGMRGRLWTLWIIQT

AGGLFCVWLGRANTLVTAVVAMVLFSMGAQAACGATFAIVPFVSRRALGIISGLTGAGGN

FGSGLTQLLFFSTSHFTTEQGLTWMGVMIVACTLPVTLVHFPQWGSMFLPPSTDPVKGTE

AHYYGSEWNEQEKQKNMHQGSLRFAENAKSEGGRRVRSAATPPENTPNNV

>AtNRT2.2

MGSTDEPGSSMHGVTGREQSYAFSVDGSEPTNTKKKYNLPVDAEDKATVFKLFSFAKPHM

RTFHLSWISFSTCFVSTFAAAPLIPIIRENLNLTKHDIGNAGVASVSGSIFSRLVMGAVC

DLLGPRYGCAFLVMLSAPTVFSMSFVSDAAGFITVRFMIGFCLATFVSCQYWMSTMFNSQ

IIGLVNGTAAGWGNMGGGITQLLMPIVYEIIRRCGSTAFTAWRIAFFVPGWLHIIMGILV

LTLGQDLPGGNRAAMEKAGEVAKDKFGKILWYAVTNYRTWIFVLLYGYSMGVELSTDNVI

AEYFFDRFHLKLHTAGIIAACFGMANFFARPAGGWASDIAAKRFGMRGRLWTLWIIQTSG

GLFCVWLGRANTLVTAVVSMVLFSLGAQAACGATFAIVPFVSRRALGIISGLTGAGGNFG

SGLTQLVFFSTSRFTTEEGLTWMGVMIVACTLPVTLIHFPQWGSMFFPPSNDSVDATEHY

YVGEYSKEEQQIGMHLKSKLFADGAKTEGGSSVHKGNATNNA

>OsNRT2.4

MVAMEKKTKLVEEEDGCYYYDYGGYGDGVVDDEGRATELRPMALSRPHTQAFHLAWMSLF

ACFFAAFAAPPILPAMRPALVLAPSDASAAAVASLSATLVGRLAMGPACDLLGPRRASGV

ASLVCALALALAAVFASSPAGFVALRFVAGLSLANFVANQHWMSRIFAPSAVGLANAVAA

GWANVGSAAAQVVMPVAYDAVVLRLGVPVTVAWRVTYLLPCAMLVTTGLAVLAFPYDLPG

GGGGRCPGGGGGRRRSFWAVVRGGVGDYRAWLLGLTYGHCYGVELIMENVAADFFRRRFR

LPMEAAGAAAACFGAMNAVARPAGGVASDEVARRFGMRGRLWALWAVQSAGAALCVLVGR

MGAAEAPSLAATVAVMVACAAFVQAASGLTFGIVPFVCKRSLGVVSGMTASGGAVGAIVT

NRLFFSGSRYTVEEAISCTGITSLLCTLPVALIHFRRQGGMFCGPSATIDGDGDVDDDDD

YMLLK

>OsNRT2.3

MEAKPVAMEVEGVEAAGGKPRFRMPVDSDLKATEFWLFSFARPHMASFHMAWFSFFCCFV

STFAVFARLAMGTACDLVGPRLASASLILLTTPAVYCSSIIQSPSGYLLVRFFTGISLAS

FVSAQFWMSSMFSAPKVGLANGVAGGWGNLGGGAVQLLMPLVYEAIHKIGSTPFTAWRIA

FFIPGLMQTFSAIAVLAFGQDMPGGNYGKLHKTGDMHKDSFGNVLRHALTNYRGWILALT

YGYSFGVELTIDNVVHQYFYDRFDVNLQTAGLIAASFGMANIISRPGGGLLSDWLSSRYG

MRGRLWGLWTVQTIGGVLCVVLGIVDFSFAASVAVMVLFSFFVQAACGLTFGIVPFVSRR

SLGLISGMTGGGGNVGAVLTQYIFFHGTKYKTETGIKYMGLMIIACTLPVMLIYFPQWGG

MLVGPRKGATAEEYYSREWSDHEREKGFNAASVRFAENSVREGGRSSANGGQPRHTVPVD

ASPAGV

>OsNRT2.1

MDSSTVGAPGSSLHGVTGREPAFAFSTEVGGEDAAAASKFDLPVDSEHKAKTIRLLSFAN

PHMRTFHLSWISFFSCFVSTFAAAPLVPIIRDNLNLTKADIGNAGVASVSGSIFSRLAMG

AICDMLGPRYGCAFLIMLAAPTVFCMSLIDSAAGYIAVRFLIGFSLATFVSCQYWMSTMF

NSKIIGLVNGLAAGWGNMGGGATQLIMPLVYDVIRKCGATPFTAWRLAYFVPGTLHVVMG

VLVLTLGQDLPDGNLRSLQKKGDVNRDSFSRVLWYAVTNYRTWIFVLLYGYSMGVELTTD

NVIAEYFYDRFDLDLRVAGIIAASFGMANIVARPTGGLLSDLGARYFGMRARLWNIWILQ

TAGGAFCLLLGRASTLPTSVVCMVLFSFCAQAACGAIFGVIPFVSRRSLGIISGMTGAGG

NFGAGLTQLLFFTSSRYSTGTGLEYMGIMIMACTLPVVLVHFPQWGSMFLPPNAGAEEEH

YYGSEWSEQEKSKGLHGASLKFAENSRSERGRRNVINAAAAAATPPNNSPEHA

>OsNRT2.2

MDSSTVGAPGSSLHGVTGREPAFAFSTEVGGEDAAAASKFDLPVDSEHKAKTIRLLSFAN

PHMRTFHLSWISFFSCFVSTFAAAPLVPIIRDNLNLTKADIGNAGVASVSGSIFSRLAMG

AICDMLGPRYGCAFLIMLAAPTVFCMSLIDSAAGYIAVRFLIGFSLATFVSCQYWMSTMF

NSKIIGLVNGLAAGWGNMGGGATQLIMPLVYDVIRKCGATPFTAWRLAYFVPGTLHVVMG

VLVLTLGQDLPDGNLRSLQKKGDVNRDSFSRVLWYAVTNYRTWIFVLLYGYSMGVELTTD

NVIAEYFYDRFDLDLRVAGIIAASFGMANIVARPTGGLLSDLGARYFGMRARLWNIWILQ

TAGGAFCLLLGRASTLPTSVVCMVLFSFCAQAACGAIFGVIPFVSRRSLGIISGMTGAGG

NFGAGLTQLLFFTSSRYSTGTGLEYMGIMIMACTLPVVLVHFPQWGSMFLPPNAGAEEEH

YYGSEWSEQEKSKGLHGASLKFAENSRSERGRRNVINAAAAAATPPNNSPEHA

>ZmNRT2.1

MAAVGAPGSSLHGVTGREPAFAFSTEHEEAASNGGKFDLPVDSEHKAKSVRLFSVANPHM

RTFHLSWISFFTCFVSTFAAAPLVPIIRDNLNLTKADIGNAGVASVSGSIFSRLTMGAVC

DLLGPRYGCAFLIMLSAPTVFCMSLIDDAAGYITVRFLIGFSLATFVSCQYWMSTMFSSK

IIGTVNGLAAGWGNMGGGATQLIMPLVYDVIRKCGATPFTAWRLAYFVPGLMHVVMGVLV

LTLGQDLPDGNLRSLQKKGNVNKDSFSKVMWYAVINYRTWIFVLLYGYCMGVELTTDNVI

AEYMYDRFDLDLRVAGTIAACFGMANIVARPMGGIMSDMGARYWGMRARLWNIWILQTAG

GAFCLWLGRASTLPVSVVAMVLFSFCAQAACGAIFGVIPFVSRRSLGIISGMTGAGGNFG

AGLTQLLFFTSSTYSTGRGLEYMGIMIMACTLPVVFVHFPQWGSMFFPPSATADEEGYYA

SEWNDDEKSKGLHSASLKFAENSRSERGKRNVIQADAAATPEHV

>ZmNRT2.2

MAAVGAPGSSLHGVTGREPAFAFSTEHEEAASNGGKFDLPVDSEHKAKSVRLFSVANPHM

RTFHLSWISFFTCFVSTFAAAPLVPIIRDNLNLTKADIGNAGVASVSGSIFSRLTMGAVC

DLLGPRYGCAFLIMLSAPTVFCMSLIDDAAGYIAVRFLIGFSLATFVSCQYWMSTMFSSK

IIGTVNGLAAGWGNMGGGATQLIMPLVYDVIRKCGATPFTAWRLAYFVPGLMHVVMGVLV

LTLGQDLPDGNLRSLQKKGNVNKDSFSKVMWYAVINYRTWIFVLLYGYCMGVELTTDNVI

AEYMYDRFDLDLRVAGTIAACFGMANIVARPMGGIMSDMGARYWGMRARLWNIWILQTAG

GAFCLWLGRASTLPVSVVAMVLFSFCAQAACGAIFGVIPFVSRRSLGIISGMTGAGGNFG

AGLTQLLFFTSSTYSTGRGLEYMGIMIMACTLPVVFVHFPQWGSMFFPPSATADEEGYYA

SEWNDDEKSKGLHSASLKFAENSRSERGKRNVIQADAAATPEHV

>ZmNRT2.3

MASDAAHGSSLDGVTPSSKFDLPVDSEHKAKTIRLLSFANPHMRTFHLSWMSFFTCVVST

FAAAPLIPIIRENLGLTKADIGNAGVASVSGAIFSRLAMGAVCDLLGPRYGCAFVVMLAA

PAVFCMAVIDSAAGYVACRFLIGFSLATFVSCQYWTSTMFNIKIIGTVNALASGWGDMGG

GATQLIMPFVYEAILRCGATPFAAWRIAYFVPGIMHIAVGILVLTAGQDLPDGNLRSLRK

QQQQQQQGDGGDASCCRRDSFSRVLWHAVANYRTWVFVFVYGYSMGVQLTTNNIIAEFYY

DQFELDIRVAGIIAACFGMANLVSRPLGGVLSDLGARYWGMRARLWNIWILQTAGGAFCF

WLGRASELPASVTAMVLFSFCAQAACGATFGVIPFVSRRSLGVISGLTGAGGNVGAGLTQ

LLFFTTSSYSTRKGIENMGIMAMACTLPLVLVHFPQWGSMLLPPSADADEERYYASEWSE

DEKSVGRHSASLKFAENSRSERGKRNAVAVLATAAATPEHVV

>ZmNRT2.5

MAEGEFKPAAMQVEAPAEAAAAPSKPRFRMPVDSDNKATEFWLFSFARPHMSAFHMSWFS

FFCCFLSTFAAPPLLPLIRDTLGLTATDIGNAGIASVSGAVFARVAMGTACDLVGPRLAS

AAIILLTTPAVYYSAVIDSASSYLLVRFFTGFSLASFVSTQFWMSSMFSPPKVGLANGVA

GGWGNLGGGAVQLIMPLVFEAIRKAGATPFTAWRVAFFVPGLLQTLSAVAVLAFGQDMPD

GNYRKLHRSGDMHKDSFGNVLRHAVTNYRAWILALTYGYCFGVELAVDNIVAQYFYDRFG

VKLSTAGFIAASFGMANIVSRPGGGLLSDWLSSRFGMRGRLWGLWVVQTIGGVLCVVLGA

VDYSFAASVAVMILFSMFVQAACGLTFGIVPFVSRRSLGLISGMTGGGGNVGAVLTQLIF

FHGSKYKTETGIKYMGFMIIACTLPITLIYFPQWGGMFLGPRPGATAEDYYNREWTAHEC

DKGFNTASVRFAENSVREGGRSGSQSKHTTVPVESSPADV

>TaNRT2-1D

MEGASNWGPAAMEVQAAPKAKFKIPVDDDSKATEFWLFSFSRSHMSAFHLSWFSFFCCFVSTFAAPPLMPLIRDNLGLTAKDIGNAGVASVSGAVFARLAMGTACDLVGPRLASAAIILLTTPAVYCTSIINSASSFLLARFFTGFSLASFVSTQFWMSSMFSAPKVGLANGVAGGWGNLGGGAVQLLMPFVFEAVRKIGSTKFVAWRVAFFIPGIMQTVSAIAVLALGQDMPDGNYRKLHKSGEMHKDSFGNVLRHAVTNYRAWILALTYGYSFGVELAVDNIVAEYFYDRFDVNLHTAGLIAATFGLANIVSRPGGGLMSDWLSQRYGMRGRLWGLWVMQTIGGVLCVVLGIVDYSFGASVAVMILFSLFCQAACGLTFGIVPFVSRRSLGLISGMTGGGGNVGAVLTQVIFFRGGKYKTETGIMYMGIMILACTLPVAFIYFPQWGGMLAGPRPGATADDYYGEWTAEERDKGYNAATKRFAENSVREGGRRAASGSDSRHTVPVDGSPAPADV

>TaNRT2-2A

MEKMETEMAAPAKIFPLPVDSEHKAKSFRLFSFAAPHMRAFHLSWMAFFICFVSTFAAAPLIPIIRDNLNLTKRDISNASVASVSGSIFSRVAMGVVCDLLGPRYGCAFLVMLTAPAVFCMSLVHDPAGYIMVRFLIGFSLATLISCQYWMSTMFSGNIIGAVNGLAAGWGNVGGGATQLVMPLVYEAIRSRCGATPFSAWRVAYFGPGTLHIVVGIMVLTLGQDLPDGNLWSLQNKGQVAKDKFAKVAWGAITNYRSWVFVLLYGYSAGVELCTDNVIAEYYYDHFHLGLRTAGTIAASFGLANIFVRSMGGYFSDVGARYFGMRARLWNIWILQTAGGAFCFWLGRASSLPASVTAMVLFSICAQAAEGAIFAVIPFVSRRSLGIVSGMTGAGGTFGAAFNQLLFFTSSNYGTGQGLQYMGIVTMACTLPVMLVHFPQWGSMLFPANVGADEEKYYGAEWSEEEKSKGLNARTVKFAQNCRSERGRHRNVILANDTNQHA

>TaNRT2-2D

METEMAAPAKMFPLAVDSEHKAKSFRLFSFAAPHMRAFHLSWMAFFVCFVSTFAAAPLIPIIRDNLNLTKRDISNASVASVSGSIFSRVAMGVVCDLLGPRYGCAFLVLLTAPAVFCMSLVHDPAGYIMVRFLIGFSLATLISCQYWMSTMFSGNIIGAVNGLAAGWGNVGGGATQLIMPLVYEAIRSRCGATPFSAWRVAYFGPGTLHIVVGIMVLTLGQDLPDGNLWSLQNKGQVAKDKFAKVAWGAITNYRSWIFVLLYGYSAGVELCTNNVIAEYYYDHFHLGLRTAGTIAASFGLANIFVRSMGGYFSDVGARYFGMRARLWNIWILQTAGGAFCFWLGRASSLPASVTAMVLFSICAQAAEGAIFAVIPFVSRRSLGIVSGMTGAGGTLGAAFNQLLFFTSSKYGTGQGLQYMGIVTMACTLPVMLVHFPQWGSMLFPANVGADEDKYYGAEWSEEEKSKGLNARTVKFAQNCRSERGRHRNAILANDTNQHA

>TaNRT2-3A

MEGESKPAAMGVQAAPKGKFRMPVDSDNKATEFWLFSFARPHMSAFHLSWFSFFCCFVSTFAAPPLLPLIRDNLGLTGKDIGNAGIASVSGAVFARLAMGTACDLVGPRLASAAIILLTTPAVYCSAIIDSASSFLLVRFFTGFSLASFVSTQFWMSSMFSSPKVGLANGVAGGWGNLGGGAVQFIMPLVYEIVRKIGSTDFVAWRIAFFIPGIMQTFSAIAVLAFGQDMPDGNYRKLHKSGEMHKDSFGNVLRHAVTNYRAWILALTYGYCFGVELAVDNIVAQYFYDRFDVNLHTAGLIAASFGMANIISRPGGGLMSDWLSDRFGMRGRLWGLWVVQTIGGVLCVVLGVVDYSFGASVAVMILFSFFVQAACGLTFGIVPFVSRRSLGLISGMTGGGGNVGAVLTQVIFFRGTKYKTETGIMYMGLMILACTLPITLIYFPQWGGMFAGPRKGATAEEYYSQEWTEEERAKGYSAATERFAENSVREGGRRAASGSQSRHTVPVDGSPADV

>TaNRT2-3B

MEGESKPAAMGVQAAPKGKFRIPVDSDNKATEFWLFSFARPHMSAFHLSWFSFFCCFVSTFAAPPLLPLIRDNLGLTGKDIGNAGIASVSGAVFARLAMGTACDLVGPRLASAAIILLTTPAVYCSAIIDSASSFLLVRFFTGFSLASFVSTQFWMSSMFSSPKVGLANGVAGGWGNLGGGAVQFIMPLVFEVVRKIGSTDFVAWRVAFFIPGIMQTFSAIAVLAFGQDMPDGNYRKLHKSGEMHKDSFGNVLRHAVTNYRAWILALTYGYCFGVELAVDNIVAQYFYDRFDVNLHTAGLIAASFGMANIISRPGGGLMSDWLSDRFGMRGRLWGLWIVQTIGGILCVVLGVVDYSFGASVAVMILFSFFVQAACGLTFGIVPFVSRRSLGLISGMTGGGGNVGAVLTQVIFFRGTTYKTETGIMYMGLMILACTLPITLIYFPQWGGMFAGPRKGATAEEYYSQEWTEEERAKGYSAATERFAENSVREGGRRAASGSQSRHTVPVDGSPADV

>TaNRT2-3D

MEGESKPAAMGVQAASKGKFRIPVDSDNKATEFWLFSFARPHMSAFHLSWFSFFCCFVSTFAAPPLLPLIRDNLGLTGKDIGNAGIASVSGAVFARLAMGTACDLVGPRLASAAIILLTTPAVYCSAIIDSASSFLLVRFFTGFSLASFVSTQFWMSSMFSSPKVGLANGVAGGWGNLGGGAVQFIMPLVFEVVRKIGSTDFVAWRVAFFIPGIMQTFSAIAVLAFGQDMPDGNYRKLHKSGEMHKDSFGNVLRHAVTNYRAWILALTYGYCFGVELAVDNIVAQYFYDRFDVNLHTAGLIAASFGMANIISRPGGGLMSDWLSDRFGMRGRLWGLWIVQTIGGILCVVLGVVDYSFGASVAVMILFSFFVQAACGLTFGIVPFVSRRSLGLISGMTGGGGNVGAVLTQVIFFRGTTYKTETGIMYMGLMILACTLPITLIYFPQWGGMFAGPRKGATAEEYYSQEWTEEERAKGYSAATERFAENSVREGGRRATSGSQSRHTVPVDGSPADV

>TaNRT2-6A.1

MEVEASSHGDAPASKFSLPVDSEHKAKSFRLFSFANPHMRTFHLSWISFFTCFVSTFAAAPLVPIIRDNLNLAKADIGNAGVASVSGSIFSRLAMGAICDLLGPRYGCAFLVMLSAPTVFCMAVIDDASGYIAVRFLIGFSLATFVSCQYWMSTMFNSKIIGTVNGLAAGWGNMGGGATQLIMPLVFHAIQKCGATPFVAWRIAYFVPGMMHIVMGLLVLTMGQDLPDGNLASLQKKGDMAKDKFSKVLWGAVTNYRTWIFVLLYGYCMGVELTTDNVIAEYYYDHFHLDLRAAGTIAACFGMANIVARPMGGYLSDLGARYFGMRARLWNIWILQTAGGAFCIWLGRASALPASVTAMVLFSICAQAACGAVFGVAPFVSRRSLGIISGLTGAGGNVGAGLTQLLFFTSSQYSTGRGLEYMGIMIMACTLPVALVHFPQWGSMFFPASADATEEEYYASEWSEEEKSKGLHIAGQKFAENSRSERGRRNVILAASATPPNNTPQHL

>TaNRT2-6A.2

MEVEAGAHGDMAASKFTLPVDSEHKAKSFRLFSFANPHMRTFHLSWISFFTCFVSTFAAAPLVPIIRDNLNLAKADIGNAGVASVSGSIFSRLAMGAICDLLGPRYGCAFLVMLSAPTVFCMAVIDDASGYIAVRFLIGFSLATFVSCQYWMSTMFNSKIIGTVNGLAAGWGNMGGGATQLIMPLVFHAIQKCGATPFVAWRIAYFVPGMMHIVMGLLVLTMGQDLPDGNLASLQKKGDMAKDKFSKVLWGAVTNYRTWIFVLLYGYCMGVELTTDNVIAEYYYDHFHLDLRAAGTIAACFGMANIVARPMGGYLSDLGARYFGMRARLWNIWILQTAGGAFCIWLGRASALPASVTAMVLFSICAQAACGAVFGVAPFVSRRSLGIISGLTGAGGNVGAGLTQLLFFTSSQYSTGRGLEYMGIMIMACTLPVALVHFPQWGSMFFPASADATEEEYYASEWSEEEKSKGLHIAGQKFAENSRSERGRRNVVLATSAMPPNNTPQHV

>TaNRT2-6A.3

MEVESSAHGDAAASKFTLPVDSEHKAKSFRLFSFANPHMRTFHLSWISFFTCFVSTFAAAPLVPIIRDNLNLAKADIGNAGVASVSGSIFSRLAMGAVCDLLGPRYGCAFLVMLSAPTVFCMAVIDDASGYIAVRFLIGFSLATFVSCQYWMSTMFNSKIIGTVNGLAAGWGNMGGGATQLIMPLVFHAIQKCGATPFVAWRIAYFVPGMMHIVMGLLVLTMGQDLPDGNLASLQKKGDMAKDKFSKVLWGAVTNYRTWIFVLLYGYCMGVELTTDNVIAEYYYDHFHLDLRAAGTIAACFGMANIVARPMGGYLSDLGARYFGMRARLWNIWILQTAGGAFCIWLGRASALPASVTAMVLFSICAQAACGAVFGVAPFVSRRSLGIISGLTGAGGNVGA

GLTQLLFFTSSQYSTGRGLEYMGIMIMACTLPITLVHFPQWGSMFFPASADATEEEYYASEWSEEEKGKGLHIAGQKFAENSRSERGRRNVILATSATPPNNTPQHV

>TaNRT2-6A.4

MEVEAGAHGDTAASKFTLPVDSEHKAKSFRLFSFANPHMRTFHLSWISFFTCFVSTFAAAPLVPIIRDNLNLAKADIGNAGVASVSGSIFSRLAMGAVCDLLGPRYGCAFLVMLSAPTVFCMAVIDDASGYIAVRFLIGFSLATFVSCQYWMSTMFNSKIIGTVNGLAAGWGNMGGGATQLIMPLVFHAIQKCGATPFVAWRIAYFVPGMMHIVMGLLVLTMGQDLPDGNLASLQKKGDMAKDKFSKVLWGAVTNYRTWIFVLLYGYCMGVELTTDNVIAEYYYDHFHLDLRAAGTIAACFGMANIVARPMGGYLSDLGARYFGMRARLWNIWILQTAGGAFCIWLGRASALPASVTAMVLFSICAQAACGAVFGVAPFVSRRSLGIISGLTGAGGNVGAGLTQLLFFTSSQYSTGRGLEYMGIMIMACTLPVTLVHFPQWGSMFFPASADATEEEYYASEWSEEEKNKGLHIAGQKFAENSRSERGRRNVILATSATPPNNTPQHV

>TaNRT2-6A.5

MEVQAGSHADSAASKFTLPVDSEHKAKSFRLFSFANPHMRTFHLSWISFFTCFVSTFAAAPLVPIIRDNLNLAKADIGNAGVASVSGSIFSRLAMGAICDLLGPRYGCAFLVMLSAPTVFCMSVIDDASGYIAVRFLIGFSLATFVSCQYWMSTMFNSKIIGTVNGLAAGWGNMGGGATQLIMPLVFHAIQKCGATPFVAWRIAYFVPGMMHIVMGLLVLTMGQDLPDGNLASLQKKGDMAKDKFSKVLWGAVTNYRTWIFVLLYGYCMGVELTTDNVIAEYYYDHFHLDLRAAGTIAACFGMANIVARPMGGYLSDLGARYFGMRARLWNIWILQTAGGAFCIWLGRASALPASVTAMVLFSICAQAACGAVFGVAPFVSRRSLGIISGLTGAGGNVGAGLTQLLFFTSSQYSTGRGLEYMGIMIMACTLPVALVHFPQWGSMFFPASADATEEEYYASEWSEEEKNKGLHIAGQKFAENSRSERGRRNVILATSATPPNNTPQHV

>TaNRT2-6A.6

MEVESSSHGAGDEAASKFSLPVDSEHKAKSFRLFSFANPHMRTFHLSWISFFTCFVSTFAAAPLVPIIRDNLNLAKADIGNAGVASVSGSIFSRLAMGAICDLLGPRYGCAFLVMLAAPTVFCMSLIDDAAGYITVRFLIGFSLATFVSCQYWMSTMFNSKIIGTVNGLAAGWGNMGGGATQLIMPLVFHAIQKCGATPFVAWRIAYFVPGMMHIVMGLMVLTMGQDLPDGNLASLQKKGDVAKDKFSKVLWGAVTNYRTWIFVLLYGYCMGVELTTDNVIAEYYFDHFHLDLRTSGTIAACFGMANLVARPMGGYLSDLGARYFGMRARLWNIWILQTAGGAFCLWLGRAKALPESVTAMVLFSVCAQAACGAVFGVIPFVSRRSLGIISGMSGAGGNFGAGLTQLLFFTSSKYGTGRGLEYMGIMIMACTLPVALVHFPQWGSMLLPPNASATEEDFYAAEWSEEEKKKGLHIPGQKFAENSRSERGRRNVILATAATPPNNTPQHA

>TaNRT2-6A.7

MEMEIGSTGATAASTNFSLPVDSEHKAKSIKIFSFSNPHMRAFHLGWMSFFTCVVSTFAAAPLIPIIRDNLNLTKADIGNAGVASVSGAIFSRLAMGAICDLLGPRYGGAFLIMLSAPAVFCMSVIDSPAGYITVRFLIGVSLATFVSCQYWISTMFNSKIIGTVGGLTAGWGDMGGGATQLIMPFVFDAIKACGATRFTAWRIAYFVPGMMLVVMGLLVLTLGQDLPDGNLRNLQKNGDMNKDKFSKVLRGAVTNYRTWIFVFIYGYCMGVELTSNNVIAGYYYDSFYLDLRKAGIIAACFGLANIFARPMGGYLSDLGARYFGMRARLWNIWILQTAGGVFCLCLGRASTLPTSIACMVLYSICVEAACGAVYGVIPFVSRRSLGLVSGMTGAGGNVGGGLTQLLFFTSSQYTTGKGLQYMGIMIMACTLPVILVHFPQWGSMLVPPSADATEEEYYAAEWTEEEKSKGLHMAGLKFAENSISERGKRNAILAVPATPPNSTPQHV

>TaNRT2-6A.8

MEMEIGSTGATAASTTFSLPVDSEHKAKSIKIFSFSNPHMRAFHLGWMSFFTCVVSTFAAAPLIPIIRDNLNLTKADIGNAGVASVSGAIFSRLAMGAICDLLGPRYGGAFLIMLSAPAVFCMSVIDSPAGYITVRFLIGVSLATFVSCQYWISTMFNSKIIGTVGGLTAGWGDMGGGATQLIMPFVFDAIKACGATRFTAWRIAYFVPGMMLVVMGLLVLTLGQDLPDGNLRSLQKNGGMNKDKFSKVLRGAITNYRTWIFVFIYGYCMGVELTSNNVIAGYYYDSFYLDLRKAGIIAACFGLANIFARPMGGYLSDLGARYFGMRARLWNIWILQTAGGVFCLCLGRASTLPTSIVCMVLYSICVEAACGAVYGVIPFVSRRSLGLVSGMTGAGGNVGGGLTQLLFFTSSQYTTGKGLQYMGIMIIACTLPVVLVHFPQWGSMLVPPSADATEEEYYSTEWTEEEKGKGLHMAGLKFAENSISERGRRNAILAVPATPPNSTPQHV

>TaNRT2-6A.9

MEMEAGSTSDTVAGRFSLPVDSENKAKSIKIFSFGNPHMRAFHLGWMSFFTCVVSTFAAAPLIPIIRDNLNLTKADIGNAGVASVSGAIFSRLAMGAICDLLGPRYGCAFLVMLSAPAVFCMSIIDGPAGYITIRFLIGVSLATFVSCQYWVSTMFNSKIIGTVGGLTAGWGDMGGGATQLIMPLVFDAIIACDATPFTAWRIAYFVPGLMLVVMGLLVLTMGQDLPDGNLRSLQKNGDMNKDKFSNVLRGAVTNYRTWIFVFIYGYCMGVELTTNNVIAEYYYDSFHLDLRAAGTIAACFGLVNIFARPMGGYLSDLGARYFGMRARLWNIWILQTAGGAFCLCLGRASALPTSITCMVLYSICIEAACGAVYGVIPFVSRRSLGLISGMSGAGGNVGGGLTQFLFFTSSQYSTGKGLQYMGIMVMACTLPVALVHFPQWGSMLLPPSANATEEDYYGAEWTEEEKNKGLHITSLKFAENSISERGRRNAILAAPVTPPNNTPQHV

>TaNRT2-6A.10

METEVGSPAAAAAAPIHFSLLVDSEHKAKSIKIFSFGNPHMRAFHLGWMSFFTCVVSTFAAAPLIPIIRDNLNLTKADIGNAGVASVSGAIFSRLAMGAICDLLGPRYGCAFLVMLSAPAVFCMSVIDGPGGYITIRFLIGVSLATFVSCQYWISTMFNSKIIGTVGGLTAGWGDMGGGATQLIMPLVFDGILACGATSFTAWRIAYFVPGMMLVVMGLLVLTMGQDLPDGNLRSLQKNGDMNKDKFTKVLRGAVTNYRTWIFVFIYGYCMGVELTTNNVIAEYYYDSFHLDLRAAGTIAASFGLANIFARPMGGYLSDLGARYFGMRARLWNIWILQTAGGVFCICLGRASSLPTSVTCMVLYSICVEAACGAVYGVIPFVSRRSLGLISGMSGAGGNVGGGLTQFLFFTSSQYTTGKGLQYMGIMIMVCTLPVALVHFPQWGSMLLPPGADATEEEYYGAEWTEEEKSKGLHIAGQKFAENSISERGKRNAILAVPSSPPNNTPQHV

>TaNRT2-6A.11

METEVGSPATATAAPINFSLPVDSEHKAKSIKIFSFGNPHMRAFHLGWMSFFTCVVSTFAAAPLIPIIRDNLNLTKADIGNAGVASVSGAIFSRLAMGAICDLLGPRYGCAFLVMLSAPAVFCMSVIDGPGGYITIRFLIGVSLATFVSCQYWISTMFNSKIIGTVGGLTAGWGDMGGGATQLIMPLVFDGILACGATRFTAWRIAYFVPGMMLVVMGLLVLTMGQDLPDGNLRSLQKNGDMNKDKFSNVLRGAVTNYRTWIFVFIYGYCMGVELTTNNVIAEYYYDSFHLDLRAAGTIAASFGLANIFARPMGGYLSDLGARYFGMRARLWNIWILQTAGGVFCICLGRASSLPTSVTCMVLYSICVEAACGAVYGVIPFVSRRSLGLISGMSGAGGNVGGGLTQFLFFTSSQYSTGKGLQYMGIMIMVCTLPITLVHFPQWGSMLLPPSADAAEEDYYGAEWTEEEKSKGLHLAGLKFAENSVSERGKRNAILAVPCSPPNNTPQHV

>TaNRT2-6A.12

MEMEVASTPTTPAPINFLLPVDSEHKAKSIKIFSFGNPHMRAFHLGWMSFFTCVVSTFAAAPLIPIIRDNLNLTKADIGNAGVASVSGAIFSRLAMGAICDLLGPRYGCAFLVMLSAPAVFCMSIIDGPGGYITIRFLIGVSLATFVSCQYWVSTMFNSKIIGTVGGLTAGWGDMGGGATQLIMPLVFDAILACGATPFTAWRLAYFVPGMMLVVMGLLVLTMGQDLPDGNLRSLQKNGDMNKDKFSNVLRGAVTNYRTWIFVFIYGYCMGVELTTNNVIAEYYYDSFHLDLRAAGTIAACFGLANVFARPMGGYLSDLGARYFGMRARLWNIWILQTAGGAFCLCLGSATTLPTSITCMVLYSICVEAACGAVYGVIPFVSRRSLGLISGMSGAGGNVGGGLTQFLFFTSSQYSTGKGLQYMGIMVMACTLPVALVHFPQWGSMLLPPTTGATEEEYYAAEWTEEEKSKGLHNAGIKFAENSVSERGRRNTILAVPATPPHVTPQHV

>TaNRT2-6A.13

MEMEVASTPTTPAPINFLLPVDSEHKAKSIKIFSFGNPHMRAFHLGWMSFFTCVVSTFAAAPLIPIIRDNLNLTKADIGNAGVASVSGAIFSRLAMGAICDLLGPRYGCAFLVMLSAPAVFCMSVIDGPSGYITIRFLIGVSLATFVSCQYWVSTMFNSKIIGTVGGLTAGWGDMGGGATQLIMPLVFDAILACGATPFMAWRLAYFVPGMMLVVMGLLVLTMGQDLPDGNLRSLQKNGDMNKDKFSNVLRGAVTNYRTWIFVFIYGYCMGVELTTNNVIAEYYYDSFHLDLRAAGTIAACFGLANIFARPMGGYLSDLSARYFGMRARLWNIWILQTAGGAFCICLGRASSLPTSITCMVLYSICVEAACGAVYGVIPFVSRRSLGLISGMSGAGGNVGGGLTQFLFFTSSQYSTGKGLQYMGIMVMACTLPVALVHFPQWGSMLLPPTASATEEDYYAAEWTEEERSKGLHNAGIKFAENSVSERGRRNAILAVPASPPHDTPQHV

>TaNRT2-6B.1

MEVESSSHGARDEAVSKFSLPVDSEHKAKSFRLFSFANPHMRTFHLSWISFFTCFVSTFAAAPLVPIIRDNLNLAKADIGNAGVASVSGSIFSRLAMGAICDLLGPRYGCAFLVMLAAPTVFCMSLIDDAAGYITVRFLIGFSLATFVSCQYWMSTMFNSKIIGTVNGLAAGWGNMGGGATQLIMPLVFHAIQKCGATPFVAWRIAYFVPGMMHIVMGLMVLTMGQDLPDGNLASLQKKGDMAKDKFSKVVWGAVTNYRTWIFVLLYGYCMGVELTTDNVIAEYYFDHFHLDLRTSGTIAACFGMANIVARPVGGYLSDLGARYFGMRARLWNIWILQTAGGAFCLWLGRAKALPESITAMVLFSICAQAACGAVFGVIPFVSRRSLGIISGLSGAGGNFGAGLTQLLFFTSSKYGTGRGLEYMGIMIMACTLPVALVHFPQWGSMLLPPNADATEEDFYAAEWSEEEKKKGLHIPGQKFAENSRSERGRRNVILATAATPPNNTPQHA

>TaNRT2-6B.2

MEVQAGSHADAAASKFTLPVDSEHKAKSFRLFSFANPHMRTFHLSWISFFTCFVSTFAAAPLVPIIRDNLNLAKADIGNAGVASVSGSIFSRLAMGAICDLLGPRYGCAFLVMLSAPTVFCMAVIDDASGYIAVRFLIGFSLATFVSCQYWMSTMFNSKIIGTVNGLAAGWGNMGGGATQLIMPLVFHAIQKCGATPFVAWRIAYFVPGMMHIVMGLLVLTMGQDLPDGNLASLQKKGDMAKDKFSKVLWGAVTNYRTWIFVLLYGYCMGVELTTDNVIAEYYYDHFHLDLRAAGTIAACFGMANIVARPMGGYLSDLGARYFGMRARLWNIWILQTAGGAFCIWLGRASALPASVTAMVLFSICAQAACGAVFGVAPFVSRRSLGIISGLTGAGGNVGA

GLTQLLFFTSSQYSTGRGLEYMGIMIMACTLPVALVHFPQWGSMFFPASADATEEEYYASEWSEEEKNKGLHIAGQKFAENSRSERGRRNVILATSVTPPNNTPQHV

>TaNRT2-6B.3

MEVQAGTHGDTATSKFTLPVDSEHKAKSIRLFSFANPHMRTFHLSWISFFTCFVSTFAAAPLVPIIRDNLNLAKADIGNAGVASVSGSIFSRLAMGAVCDLLGPRYGCAFLVMLSAPTVFCMAVIDDASGYIAVRFLIGFSLATFVSCQYWMSTMFNSKIIGTVNGLAAGWGNMGGGATQLIMPLVFHAIQKCGATPFVAWRIAYFVPGMMHIVMGLLVLTLGQDLPDGNLASLQKKGDMAKDKFSKVLWGAVTNYRTWIFVLLYGYCMGVELTTDNVIAEYYYDHFHLDLRAAGTIAACFGMANIVARPMGGYLSDLGARYFGMRARLWNIWILQTAGGAFCIWLGRASALPASVTAMVLFSICAQAACGAVFGVAPFVSRRSLGIISGLTGAGGNVGAGLTQLLFFTSSQYSTGRGLEYMGIMIMACTLPVTLVHFPQWGSMFFPASADATEEEYYGSEWSEEEKRNGLHIAGQKFAENSRSERGRRNVILATSATPPNNTPQHV

>TaNRT2-6B.4

MEVEASAHGDTAASKFTLPVDSEHKAKSFRLFSFANPHMRTFHLSWISFFTCFVSTFAAAPLVPIIRDNLNLAKADIGNAGVASVSGSIFSRLAMGAICDLLGPRYGCAFLVMLSAPTVFCMAAIDDASGYIAVRFLIGFSLATFVSCQYWMSTMFNSKIIGTVNGLAAGWGNMGGGATQLIMPLVFHAIQKCGATPFVAWRIAYFVPGMMHIVMGLLVLTMGQDLPDGNLASLQKKGDMAKDKFSKVLWGAVTNYRTWIFVLLYGYCMGVELTTDNVIAEYYYDHFHLDLRAAGTIAACFGMANIVARPMGGYLSDLGARYFGMRARLWNIWILQTAGGAFCIWLGRASALPASVTAMVLFSICAQAACGAVFGVAPFVSRRSLGIISGLTGAGGNVGAGLTQLLFFTSSQYSTGRGLEYMGIMIMACTLPVALVHFPQWGSMFFPASADATEEEYYASEWSEEEKGKGLHITGQKFAENSRSERGRRNVILATSATPPNNTPQHV

>TaNRT2-6B.5

MSTPALYPTINTQSIQDPKHQTTQPLATPSCRPLQVAKLSSKELAEKKPSSVRTGEADMEVEASAHGDTAASKFTLPVDSEHKAKSFRLFSFANPHMRTFHLSWISFFTCFVSTFAAAPLVPIIRDNLNLAKADIGNAGVASVSGSIFSRLAMGAICDLLGPRYGCAFLVMLSAPTVFCMAVIDDASGYIAVRFLIGFSLATFVSCQYWMSTMFNSKIIGTVNGLAAGWGNMGGGATQLIMPLVFHAIQKCGATPFVAWRIAYFVPGMMHIVMGLLVLTMGQDLPDGNLASLQKKGDMAKDKFSKVLWGAVTNYRTWIFVLLYGYCMGVELTTDNVIAEYYYDHFHLDLRAAGTIAACFGMANIVARPMGGYLSDLGARYFGMRARLWNIWILQTAGGAFCIWLGRASALPASVTAMVLFSICAQAACGAVFGVAPFVSRRSLGIISGLTGAGGNVGAGLTQLLFFTSSQYSTGRGLEYMGIMIMACTLPVALVHFPQWGSMFFPASADATEEEYYASEWSEEEKSKGLHIAGQKFAENSRSERGRRNVVLATSATPPNNTPQHV

>TaNRT2-6B.6

MEVEAGSHADTASKFTLPVDSEHKAKSFRLFSFANPHMRTFHLSWISFFTCFISTFARRLSSPSSATTSTLPRPTSAMLAWHPCPAPSSRGSPWEPFFCMAVIDDASGYIAVRFLIGFSLATFVSCQYWMSTMFNSKIIGTVNGLAAGWGNMGGGATQLIMPLVFHAIQKCGATPFVAWRIAYFVPGMMHIVMGLLVLTMGQDLPDGNLASLQKKGDMAKDKFSKVLWGAVTNYRTWIFVLLYGYCMGVELTTDNVIAEYYYDHFHLDLRAAGTIAACFGMANIVARPMGGYLSDLGARYFGMHARLWNIWILQTTGGAFCIWLGRASALPASVTAMVLFSICAQAACGAVFRVAPFVSRRSLGIISALTGAGGNVGAGLTQLLFFTSSQYSTGRGLEYMGIMIMACTLPVALVHFPQWGSMFFPASADATEEEYYASEWSEEEKSKGLHIAGQKFAENSRSERGRRNVILATSATPPNNTPQHV

>TaNRT2-6B.7

MEMEIGSPGAIAASTNFSLPVDSEHKAKSIKIFSFGNPHMRAFHLGWMSFFTCVVSTFAAAPLIPIIRDNLNLTKADIGNAGVASVSGAIFSRLAMGAICDLLGPRYGGAFLIMLSAPAVFCMSVIDSPAGYIIVRFLIGVSLATFVSCQYWISTMFNSKIIGTVGGLTAGWGDMGGGATQLIMPFVFDAIKACGATRFTAWRIAYFVPGMMLVVIGLLVLTLGQDLPDGNLRNLQKNGDMNKDKFSKVLRGAVTNYRTWIFVFIYGYCMGVELTSNNVIAGYYYDSFYLDLRKAGIIAACFGLANIFARPMGGYLSDLGARYFGMRARLWNIWILQTAGGVFCLCLGRASTLPTSIACMVLYSICVEAACGAVYGVIPFVSRRSLGLVSGMTGAGGNVGGGLTQLLFFTSSQYTTSKGLQYMGIMIMACTLPVILVHFPQWGSMLVPPSPDATEEEYYAAEWTEEEKGKGLHMAGLKFAENSISERGRRNAILAVPATPPNSTPQHV

>TaNRT2-6B.8

MEIGSTGTTAASTNFSLPVDSEHKAKSIKIFSFGNPHMRAFHLGWMSFFTCVVSTFAAAPLIPIIRDNLNLTKADICNAGVASVSGAIFSRLAMGAICDLLGPRYGGAFLIMLSAPAVFCMSVIDSPAGYITVRFLIGVSLATFVSCQYWISTMFNSKIIGTVGGLTAGWGDMGGGATQLIMPFVFDAIKACGATRFTAWRIAYFVPGMMLVVMGLLVLTLGQDLPDGNLRSLQKNGGMNKDKFSKVLRGAITNYRTWIFVFIYGYCMGVELTSNNVIAGYYYDSFYLDLRKAGIIAACFGLANIFARPMGGYLSDLGARYFGMRARLWNIWILQTAGGVFCLCLGRASTLPTSIVCMVLYSICVEAACGAVYGVIPFVSRRSLGLVSGMTGAGGNVGGGLTQLLFFTSSQYTTGKGLQYMGIMIIACTLPVVLVHFPQWGSMLVPPSADATEGEYYSSEWTEEEKGKGLHMAGLKFAENSISERGRRNAILAVPATPPNSTPQHV

>TaNRT2-6B.9

MEMETGSTGDTVAGRFSLPVDSENKAKSIKIFSFGNPHMRAFHLAWMSFFTCVVSTFAAAPLIPIIRDNLNLTKADIGNAGVASVSGAIFSRLAMGAICDLLGPRYGCAFLVMLSAPAVFCMSIIDGPAGYITIRFLIGVSLATFVSCQYWVSTMFNSKIIGTVGGLTAGWGDMGGGATQLIMPLVFDAIIACGATPFTAWRIAYFVPGLMLVVMGLLVLTMGQDLPDGNLRSLQKNGDMNKDKFSNVLRGAVTNYRTWIFVFIYGYCMGVELTTNNVIAEYYYDSFHLDLRAAGTIAACFGLANIFARPMGGYLSDLGARYFGMRARLWNIWILQTAGGAFCLCLGRASTLPTSITCMVLYSICVEAACGAVYGVIPFVSKRSLGLISGMSGAGGNVGGGLTQFLFFTSSQYSTGKGLQYMGIMVMACTLPVALVHFPQWGSMLLPPSADATEEDYYGAEWTEEEKNKGLHIASLKFAENSISERGRRNAILAAPATPPNNTPQHI

>TaNRT2-6B.10

MEMEVGSPAATTAAPINFSLPVDSEHKAKSIKIFSFGNPHMRAFHLGWMSFFTCVVSTFAAAPLIPIIRDNLNLTKADIGNAGVASVSGAIFSRLAMGAICDLLGPRYGCAFLVMLSAPAVFCMSVIDGPGGYITIRFLIGVSLATFVSCQYWISTMFNSKIIGTVGGLTAGWGDMGGGATQLIMPLVFDGILACGATPFTAWRLAYFVPGMMLVVMGLLVLTLGQDLPDGNLRSLQKNGDMNKDKFTKVLQGAVTNYRTWIFVFIYGYCMGVELTTNNVIAEYYYDSFHLDLRAAGTIAASFGLANIFARPMGGYLSDLGARYFGMRARLWNIWILQTAGGVFCICLGRASSLPTSVTCMVLYSICVEAACGAVYGVIPFVSRRSLGLISGMSGAGGNVGGGLTQFLFFTSSQYTTGKGLQYMGIMIMVCTLPIALVHFPQWGSMLLPPSADATEEDYYSAEWTEEEKSKGLHLGGLKFAENSVSERGKRNAILAVPSSPPNNTPQHV

>TaNRT2-6B.11

MEMEVPSTPTTPAPINFLLPVDSEHKAKSIKIFSFGNPHMRAFHLGWMSFFTCVVSTFAAAPLIPIIRDNLNLTKADIGNAGVASVSGAIFSRLAMGAICDLLGPRYGCAFLVMLSAPAVFCMSVIDGPGGYITIRFLIGVSLATFVSCQYWVSTMFNSKIIGTVGGLTAGWGDMGGGATQLIMPLIFDAILACGATPFTAWRLAYFVPGMMLVVMGLLVLTMGQDLPDGNLRSLQKNGDMNKDKFSNVLRGAVTNYRTWIFVFIYGYCMGVELTTNNVIAEYYYDSFHLDLRAAGTIAACFGLANVFARPMGGYLSDLAARYFGMRARLWNIWILQTAGGAFCICLGRASTLPTSITCMVLYSICVEAACGAVYGVIPFVSRRSLGLISGMSGAGGNVGGGLTQFLFFTSSQYSTGKGLQYMGIMVMACTLPVALVHFPQWGSMLLPPTEGATEEDYYAAEWTEEEKSKGLHNAGIKFAENSVSERGRRNAILAVPASPPHVTPQHV

>TaNRT2-6D.1

MEVESSSHGAGDEAASKFSLPVDSEHKAKSFRLFSFANPHMRTFHLSWISFFTCFVSTFAAAPLVPIIRDNLNLAKADIGNAGVASVSGSIFSRLAMGAICDLLGPRYGCAFLVMLAAPTVFCMSLIDDAAGYITVRFLIGFSLATFVSCQYWMSTMFNSKIIGTVNGLAAGWGNMGGGATQLIMPLVFHAIQKCGATPFVAWRIAYFVPGLMHVVMGLLVLTMGQDLPDGNLASLQKKGDMAKDKFSKVVWGAVTNYRTWIFVLLYGYCMGVELTTDNVIAEYYFDHFHLDLRTSGTIAACFGMANLVARPMGGYLSDLGARYFGMRARLWNIWILQTAGGAFCLWLGRAKALPESITAMVLFSICAQAACGAIFGVIPFVSRRSLGIISGLSGAGGNFGAGLTQLLFFTSSKYGTGMGLEYMGIMIMACTLPVVLVHFPQWGSMLLPPNANATEEDFYAAEWSEEEKKKGLHIPGQKFAENSRSERGRRNVILATAATPPNNTPQHA

>TaNRT2-6D.2

MLSAPTVFCMAVIDDASGYIAVRFLIGFSLATFVSCQYWMSTMFNSKIIGTVNGLAAGWGNMGGGATQLIMPLVFHAIQKCGATPFVAWRIAYFVPGMMHIVMGLLVLTMGQDLPDGNLASLQKKGDMAKDKFSKVLWGAVTNYRTWIFVLLYGYCMGVELTTDNVIAEYYYDHFHLDLRAAGTIAACFGMANIVARPMGGYLSDLGARYFGMRARLWNIWILQTAGGAFCIWLGRASALPASVTAMVLFSICAQAACGAVFGVAPFVSRRSLGIISGLTGAGGNVGAGLTQLLFFTSSQYSTGRGLEYMGIMIMACTLPVALVHFPQWGSMFFPASADATEEEYYASEWSEEEKNKGLHIAGQKFAENSRSERGRSNVILATSATPPNNTPQHV

>TaNRT2-6D.3

MEVEAGAHGDTAASKFTLPVDSEHKAKSFRLFSFANPHMRTFHLSWISFFTCFVSTFAAAPLVPIIRDNLNLAKADIGNAGVASVSGSIFSRLAMGAVCDLLGPRYGCAFLVMLSAPTVFCMAVIDDASGYIAVRFLIGFSLATFVSCQYWMSTMFNSKIIGTVNGLAAGWGNMGGGATQLIMPLVFHAIQKCGATPFVAWRIAYFVPGMMHIVMGLLVLTMGQDLPDGNLASLQKKGDMAKDKFSKVLWGAVTNYRTWIFVLLYGYCMGVELTTDNVIAEYYYDHFHLDLRAAGTIAACFGMANIVARPMGGYLSDLGARYFGMRARLWNIWILQTAGGAFCIWLGRASALPASVTAMVLFSICAQAACGAVFGVAPFVSRRSLGIISGLTGAGGNVGA

GLTQLLFFTSSQYSTGRGLEYMGIMIMACTLPVALVHFPQWGSMFFPASADATEEEYYGSEWSEEEKRNGLHIAGQKFAENSRSERGRRNVILATSATPPNNTPQHV

>TaNRT2-6D.4

MEVEAGAHGDTAASKFTLPVDSEHKAKSFRLFSFANPHMRTFHLSWISFFTCFVSTFAAAPLVPIIRDNLNLAKADIGNAGVASVSGSIFSRLAMGAICDLLGPRYGCAFLVMLSAPTVFCMAVIDDASGYIAVRFLIGFSLATFVSCQYWMSTMFNSKIIGTVNGLAAGWGNMGGGATQLIMPLVFHAIQKCGATPFVAWRIAYFVPGMMHIVMGLLVLTMGQDLPDGNLASLQKKGDMAKDKFSKVLWGAVTNYRTWIFVLLYGYCMGVELTTDNVIAEYYYDHFHLDLRAAGTIAACFGMANIVARPMGGYLSDLGARYFGMRARLWNIWILQTAGGAFCIWLGRASALPASVTAMVLFSICAQAACGAVFGVAPFVSRRSLGIISGLTGAGGNVGAGLTQLLFFTSSQYSTGRGLEYMGIMIMPCTLPVALVHFPQWGSMFFPASADATEEEYYASEWSEEEKGKGLHIAGQKFAENSRSERGRRNVILATSATPPNNTPQHV

>TaNRT2-6D.5

MAYFVPGMMHIVMGLLVLTMGQDLPDGNLASLQKKGDMAKDKFSKVLWGAVTNYRTWIFVLLYGYCMGVELTTDNVIAEYYYDHFHLDLRAAGTIAACFGMANIVARPMGGYLSDLGARYFGMRARLWNIWILQTAGGAFCIWLGRALALPASVTAMVLFSICAQAACGAVFGVAPFVSRRSLGIISGLTGAGGNVGAGLTQLLFFTSSQYSTGRGLEYMGIMIMACTLPVALVHFPQWGSMFFPASADATEEEYYASEWSEEEKSKGLHIAGQKFAENSRSERGRRNVVLATSATPPNNTPQHV

>TaNRT2-6D.6

MEVEASGHGDAAASKFTLPVDSEHKAKSFRLFSFANPHMRTFHLSWISFFTCFVSTFAAAPLVPIIRDNLNLAKADIGNAGVASVSGSIFSRLAMGAICDLLGPRYGCAFLVMLSAPTVFCMAVIDDASGYIAVRFLIGFSLATFVSCQYWMSTMFNSKIIGTVNGLAAGWGNMGGGATQLIMPLVFHAIQKCGATPFVAWRIAYFVPGMMHIVMGLLVLTMGQDLPDGNLASLQKKGDMAKDKFSKVLWGAVTNYRTWIFVLLYGYCMGVELTTDNVIAEYYYDHFHLDLRAAGTIAACFGMANIVARPMGGYLSDLGARYFGMRARLWNIWILQTAGGAFCIWLGRASALPASVTAMVLFSICAQAACGAVFGVAPFVSRRSLGIISGLTGAGGNVGAGLTQLLFFTSSQYSTGRGLEYMGIMIMACTLPVALVHFPQWGSMFFPASTDATEEEYYASEWSEEEKSKGLHIAGQKFAENSRSERGRRNVILATSATPPNNTPQHV

>TaNRT2-6D.7

MEMEIGSTGATAASTNFSLPVDSEHKAKSIKIFSFGNPHMRAFHLGWMSFFTCVVSTFAAAPLIPIIRDNLNLTKADIGNAGVASVSGAIFSRLAMGAICDLLGPRYGGAFLIMLSAPAVFCMSVVDSPAGYIIVRFLIGVSLATFVSCQYWISTMFNSKIIGTVGGLTAGWGDMGGGATQLIMPFVFDAIKACGATRFTAWRIAYFVPGMMLVVMGLLVLTLGQDLPDGNLRNLQKNGDMNKDKFSKVLRGAVTNYRTWIFVFIYGYCMGVELTSNNVIAGYYYDSFYLDLRKAGIIAACFGLANIFARPMGGYLSDLGARYFGMRARLWNIWILQTAGGVFCLCLGRASTLPTSIACMVLYSICVEAACGAVYGVIPFVSRRSLGLVSGMTGAGGNVGGGLTQLLFFTSSQYTTGKGLQYMGIMIMACTLPVILVHFPQWGSMLVPPSMDATEEEYYAAEWTEEEKGKGLHMAGLKFAENSISERGRRNAILAVPATPPNSTPQHV

>TaNRT2-6D.8

MEMEIGSTGATAASTNFSLPVDSEHKAKSIKIFSFGNPHMRAFHLGWMSFFTCVVSTFAAAPLIPIIRDNLNLTKADIGNAGVASVSGAIFSRLAMGAICDLLGPRYGGAFLIMLSAPAVFCMSVIDSPAGYITVRFLIGVSLATFVSCQYWISTMFNSKIIGTVGGLTAGWGDMGGGATQLIMPFVFDAIKACGATRFTAWRIAYFVPGMMLVVMGLLVLTLGQDLPDGNLRSLQKNGGMNKDKFSKVLRGAITNYRTWIFVFIYGYCMGVELTSNNVIAGYYYDSFYLDLRKAGIIAACFGLANIFARPMGGYLSDLGARYFGMRARLWNIWILQTAGGVFCLCLGRASTLPTSIACMVLYSICVEAACGAVYGVIPFVSRRSLGLVSGMTGAGGNVGGGLTQLLFFTSSQYTTGKGLQYMGIMIMACTLPVILVHFPQWGSMLVPPSADATEEEYYAAEWTEEEKGKGLHMAGLKFAENSISERGRRNAILAVPATPPNSTPQHV

>TaNRT2-6D.9

MEMEAGSMGDTVAGRFSLPVDSENKAKSIKIFSFGNPHMRAFHLGWMSFFTCVVSTFAAAPLIPIIRDNLNLTKADIGNAGVASVSGAIFSRLAMGAICDLLGPRYGCAFLVMLSAPAVFCMSIIDGPAGYITIRFLIGVSLATFVSCQYWVSTMFNSKIIGTVGGLTAGWGDMGGGATQLIMPLVFDAIIACGATPFTAWRIAYFVPGLMLVVMGLLVLTTGQDLPDGNMRSLQKNGDMNKDKFSNVLRGAVTNYRTWIFVFIYGYCMGVELTTNNVIAEYYYDSFHLDLRAAGTIAASFGLANIFARPMGGYLSDLGARYFGMRARLWNIWILQTAGGAFCLCLGRASTLPTSITCMVLYSICVEAACGAVYGVIPFVSRRSLGLISGMSGAGGNVGGGLTQFLFFTSSQYSTGKGLQYMGIMVMACTLPVALIHFPQWGSMLLPPSADATEEDYYGAEWTEEEKNKGLHIASLKFAENSISERGRRNAILAAPTTPPNNTPQHV

>TaNRT2-6D.10

METEVGSPAAATAAPINFSLPVDSEHKAKSIKIFSFGNPHMRAFHLGWMSFFTCVVSTFAAAPLIPIIRDNLNLTKADIGNAGVASVSGAIFSRLAMGAICDLLGPRYGCAFLVMLSAPAVFCMSVIDGPGGYITIRFLIGVSLATFVSCQYWISTMFNSKIIGTVGGLTAGWGDMGGGATQLIMPLVFDGILACGATSFMAWRIAYFVPGMMLVVMGLLVLTMGQDLPDGNLRSLQKNGDMNKDKFSKVLRGAVTNYRTWIFVFIYGYCMGVELTTNNVIAEYYYDSFHLDLRAAGTIAASFGLANIFARPMGGYLSDLGARYFGMRARLWNIWILQTAGGVFCICLGRASSLPTSVTCMVLYSICVEAACGAVYGVIPFVSRRSLGLISGMSGAGGNVGGGLTQFLFFTSSQYTTGKGLQYMGIMIMVCTLPVALVHFPQWGSMLLPPSADATEEEYYGAEWTEEEKSKGLHIAGQKFAENSISERGKRNAILAVPSSPPNSTPLHV

>TaNRT2-6D.11

MEAGSSAAAATPPITFSLPVDSEHKAMCIKIFSFGNPHMRAFHLGWMSFFTCVVSTFAAAPLIPIIRDNLNLTKADIGNAGVASVSGAIFSRLAMGAICDLLGPCYGCAFLVMLSAPAVFCMSVIDGPGGYITIRFLIGVSLATFVSCQYWISTMFSSKIIGMVGGLMAGWGDMGGGATQLIMPLVFDGILACGATRFTAWRIAYFVPGMMLVLMGLLVLTMGQDLPDGNLRSLQKNGDMNKDKFSKVLRGAVTNYRTWIFVFIYGYCMGVELTTNNVIAEYYYDSFHLDLRAAGTIAASFGLANIFARPMGGYLSDLGARYFGMRARLWNIWILQTAGGVLCICLGRASSLPTSVTCMVLYSTCVEDACGAVYGVIPFVSRRSLGLISGMSGAGGNVGGGLTQFLFFTSSQYTTSKGLQYMGIMIMVCTLPVALVHFPQWGSMLLPPSTDATEEEYYGAKWTEEEKSKGLHIAGQKFAENSISERGKRNAILAVPSSQPNNTPQHV

>TaNRT2-6D.12

MEMEVGSPDATGAAPINFALPVDSEHKAKSIKIFSFGNPHMRAFHLGWMSFFTCVVSTFAAAPLIPIIRDNLNLTKADIGNAGVASVSGAIFSRLAMGAICDLLGPRYGCAFLVMLSAPAVFCMSVIDGPGGYITIRFLIGVSLATFVSCQYWISTMFNSKIIGTVGGLTAGWGDMGGGATQLIMPLVFDGILACGATRFTAWRLAYFVPGMMLVVMGLLVLTMGQDLPDGNLGSLQKNGDMNKDKFSKVLRGAVTNYRTWIFVFIYGYCMGVELTTNNVIAEYYYDSFHLDLRAAGTIAASFGLANIFARPMGGYLSDLGARYFGMRARLWNVWILQTAGGVFCICLGRASSLPISVTFMVLYSISVEAACGAVYGVIPFISRRSLGLISGMSGAGGNVGGGLTQFLFFTSSQYTTGKGLQYMGIMIIVCTLPVTLVHFPQWGSMLLPPRADATEEEYYGAEWTEEEKSKGLHLAGVKFAENSVSERGKRNAILAVPSSPPNNTPQHV

>TaNRT2-6D.13

MEMEVASTPTTPAPINFLLPVDSEHKAKSIKIFSFGNPHMRAFHLGWMSFFTCVVSTFAAAPLIPIIRDNLNLTKADIGNAGVASVSGAIFSRLAMGAICDLLGPRYGCAFLVMLSAPAVFCMSVIDGPGGYITIRFLIGISLATFVSCQYWVSTMFNSKIIGTVGGLTAGWGDMGGGATQLIMPLVFDAILACGATPFTAWRLAYFVPGMMLVVMGLLVLTMGQDLPDGNLRSLQKNGDMNKDKFSNVLRGAVTNYRTWIFVFIYGYCMGVELTTNNVIAEYYYDSFHLDLRAAGTIAACFGLANVFARPMGGYLSDLGARYFGMRARLWNIWILQTAGGAFCLCLGRATTLPTSITCMVLYSICVEAACGAVYGVIPFVSRRSLGLISGMSGAGGNVGGGLTQFLFFTSSQYSTGKGLQYMGIMVMACTLPVALVHFPQWGSMLLPPTAGATEEDYYAAEWTEEKSKGLHNAGIKFA

ENSVSERGRRNAILAVPGTPPHVTPQHV

>TaNRT2-6D.14

MEMEVASTPTTPAPINFLLPVDSEHKAKSIKIFSFGNPHMRAFHLGWMSFFTCVVSTFAAAPLIPIIRDNLNLTKADIGNAGVASVSGAIFSRLAMGAICDLLGPRYGCAFLVMLSAPAVFCMSVIDGPSGYITIRFLIGVSLATFVSCQYWVSTMFNSKIIGTVGGLTAGWGDMGGGATQLIMPLVFDAILACGATPFTAWRLAYFVPGMMLVVMGLLVLTMGQDLPDGNLRSLQKNGDMNKDKFSNVLRGAVTNYRTWIFVFIYGYCMGVELTTNNVIAEYYYDSFHLDLRAAGTIAACFGLANVFARPMGGYLSDLGARYFGMRARLWNIWILQTAGGAFCLCLGRATTLPTSITCMVLYSICVEAACGAVYGVIPFVSRRSLGLISGMSGAGGNVGGGLTQFLFFTSSQYSTGKGLQYMGIMVMACTLPVALVHFPQWGSMLLPPTAGATEEEYYAAEWTEEEKSKGLHNAGIKFAENSVSERGRRNAILAVPATPSHDTPQQV

>TaNRT2-7A

MVTMGKKVDQEQSYYNDWAHIDHGVDADGRATELRPLALSRPHTQAFHLAWLSLFACFFA

AFAAPPILPALRPALVLAPSDASAAAVASLSAALVGRLAMGAACDLLGPRRASGVASLVC

ALALALAAVYASSPAGFVALRFCAGLSLSNFVANQHWMSRIFAPSGVGLANAVAAGWANV

GSAAAQVVMPLAYDLIVLRLGVPITVAWRVAYLIPCAMLITTGLAVLAFPYDLPSGCAYA

GGGKGAKGEGFWKVVRGGVCDYRAWVLALTYGYCYGVELIMENVAADFFRRRFRLPMEAA

GAAAACFGVMNTVARPAGGVASDVVGRRFGMRGRLWALWAVQSTGAVLCVLVGRMGATEA

PSLAATMAVMVACGAFVQAASGLTFGIVPFVSKRSMGVVSGMTASGRRGCRRDRDKPVVL

QQLQVHGGGGHLIHWPHQPPLHAPCGAHLFPTLGRDALRPLGNRHRRP

>TaNRT2-7B

MVTMGKKVDQEQSYYSDWAHIDHGVDADGRATELRPLALSRPHTQAFHLAWLSLFACFFA

AFAAPPILPALRPALVLAPADASAAAVGSLSAALVGRLAMGPACDLLGPRRASGVASLVC

ALALALAAVYASSPAGFVALRFCAGLSLSNFVANQHWMSRIFAPSGVGLANAVAAGWANV

GSAAAQVVMPLAYDLIVLRLGVPITVAWRVAYLIPCAMLITTGLAVLAFPYDLPSGCAYA

GGAKRAKGEGFWNVVRGGVSDYRAWVLALTYGYCYGVELIMENVAADFFRRRFRLPMEAA

GAAAACFGVMNTVARPAGGVASDEVGRRFGMRGRLWALWAVQSTGAVLCVLVGRMGATEA

PSLAATMAVMVACGAFVQAASGLTFGIVPFVSKRSMGVVSGMTASGGAVGAIVTNRLFFS

SSRYTVEEAISFTGLTSLLCTLPVALIYFPRLGGMLCGPSESATVDHDGHDDDDDVNKDD

DYMLLK

>TaNRT2-7D

MVTMGKKVDQEQSYYSDWAHIDHGVDADGRATELRPLALSRPHTQAFHLAWLSLFACFFA

AFAAPPILPALRPALVLAPSDASAAAVASLSAALVGRLAMGPACDLLGPRRASGVASLVC

ALALALAAVYASSPAGFVALRFCAGLSLSNFVANQHWMSRIFAPSGVGLANAVAAGWANV

GSAAAQVVMPLAYDLIVLRLGVPITVAWRVAYLIPCAMLIATGLAVLAFPYDLPSGCTYA

GGAKGEGFWKVVRGGVSDYRAWVLALTYGYCYGVELIMENVAADFFRRRFRLPMEAAGAA

AACFGVMNTVARPAGGVASDVVGRRFGMRGRLWALWAVQSTGAVLCVMVGRMGATEAPSL

AATMAVMVACGAFVQAASGLTFGIVPFVSKRSMGVVSGMTASGGAVGAIVTNRLFFSSSR

YTVEEAISFTGLTSLLCTLPVALIYFPRLGGMLCGPSESDTVDHDGHDDDDDVNKDDDYM

LLK

>TaNRT2-U.1

MEGAAAMEMEVQAAPKAKFRIPVDDDSKATEFWLFSFARPHMSAFHLSWFSFFCCFVSTFAAPPLMPLIRDNLGLTAKDIGNAGVASVSGAVFARLAMGTACDLVGPRLASAAIILLTTPAVYCTSIINSASSFLLARFFTGFSLASFVSTQFWMSSMFSAPKVGLANGVAGGWGNLGGGAVQLLMPFVFEAVRKMGSTKFVAWRVAFFIPGIMQTVSAIAVLALGQDMPDGNYRKLHKSGEMHKDSFGNVLRHAVTNYRAWILALTYGYSFGVELAVDNIVAEYFYDRFDVNLKTAGLIAATFGLANIVSRPGGGLMSDWLSQRYGMRGRLWGLWVMQTIGGVLCVVLGIVDYSFGASVAVMILFSLFCQAACGLTFGIVPFVSRRSLGLISGMTGGGGNVGAVLTQVIFFRGGKYKTETGIMYMGIMILACTLPVAFIYFPQWGGMLAGPRKGATADDYYNGEWTAEEREKGYNAATKRFAENSVREGGRRAASGSRSRHTVPVDSSPAPADV

>TaNRT2-U.2

MRAFHLGWMSFFTCVVSTFAAAPLIPIIRDNLNLTKADIGNAGVASVSGAIFSRLAMGAICDLLGPRYGCAFLVMLSAPAVFCMSVIDGPGGYITIRFLIGVSLATFVSCQYWISTMFNSKIIGTVGGLTAGWGDMGGGATQLIMPLVFDGILACGATSFTAWRIAYFVPGMMLVVMGLLVLTMGQDLPDGNLRNLQKNGDMNKDKFTKVLRGAVTNYRTWIFVFIYGYCMGIELTTNNVIAEYYYDSFHLDLRAAGTI
