# Supplementary material for: Genome-wide systematic characterization of the NRT2 gene family and its expression profile in wheat (Triticum aestivum L.) during plant growth and in response to nitrate deficiency
Source: BMC Plant Biol. 2023 Jul 7;23:353. doi: 10.1186/s12870-023-04333-5 (PMC10327373; doi:10.1186/s12870-023-04333-5)
Supplement: Supplementary file 6 — Additional file 6: Fig. S1. The number and ratio of NRT2 genes in wheat, rice, maize and Arabidopsis. a The number of NRT2 genes in wheat genome and sub-genome, rice, maize and Arabidopsis. b The ratio of total NRT2 gene is shown for wheat : rice (red) and wheat : Arabidopsis (orange). The expected ratio (3 : 1) is indicated by a black dotted line. Fig. S2. Gene classification was based on GO analysis for DEGs under nitrate deficiency condition. The numbers of DEGs in each GO term was significantly enriched in root (a) and shoot (b). Functional categorization of genes based on the biological process of gene ontology. Different classes are shown for BP (biological process) ,CC (cellular component) and MF (molecular function). The y‑axis shows the counts of differently expressed genes, and the x‑axis shows GO term of gene enriched in each biological process. Fig. S3. K15NO3 uptake into Xenopus oocytes. oocytes injected with water as control, cRNA of TaNRT2-6A.2, TaNRT2-6A.6, TaNRT2-6B.4 were injected alone, respectively.15N enrichment per oocyte is expressed as delta 15N compared with standard atmospheric 15N : 14N ratio. Values are average of n = 6 ± SD. Differences between mean values of treatments and controls were compared using t - tests (* P< 0.05). Fig. S4. Heatmap representing the expression pattern of TaNLP genes in various developmental stages. The TPM values normalized by logarithmic scale were used to construct the heatmap. Z10~Z85 represent different growth stage of wheat. Different colors represent relative expression levels, as shown in the legend on the right. The horizontal axis represents the names and classifications genes, and the vertical axis represents various tissues. The rows of the heat map are clustered according to the expression patterns. Fig. S5. Yeast one-hybrid (Y1H) assay was used to verify TaNLPs bound to the TaNRT2s promoter region. TaNLPs fusion proteins activate the expression of LacZ reporter gene driven by the promoter of TaNRT2- [file 12870_2023_4333_MOESM6_ESM.pptx]

## Slide 1
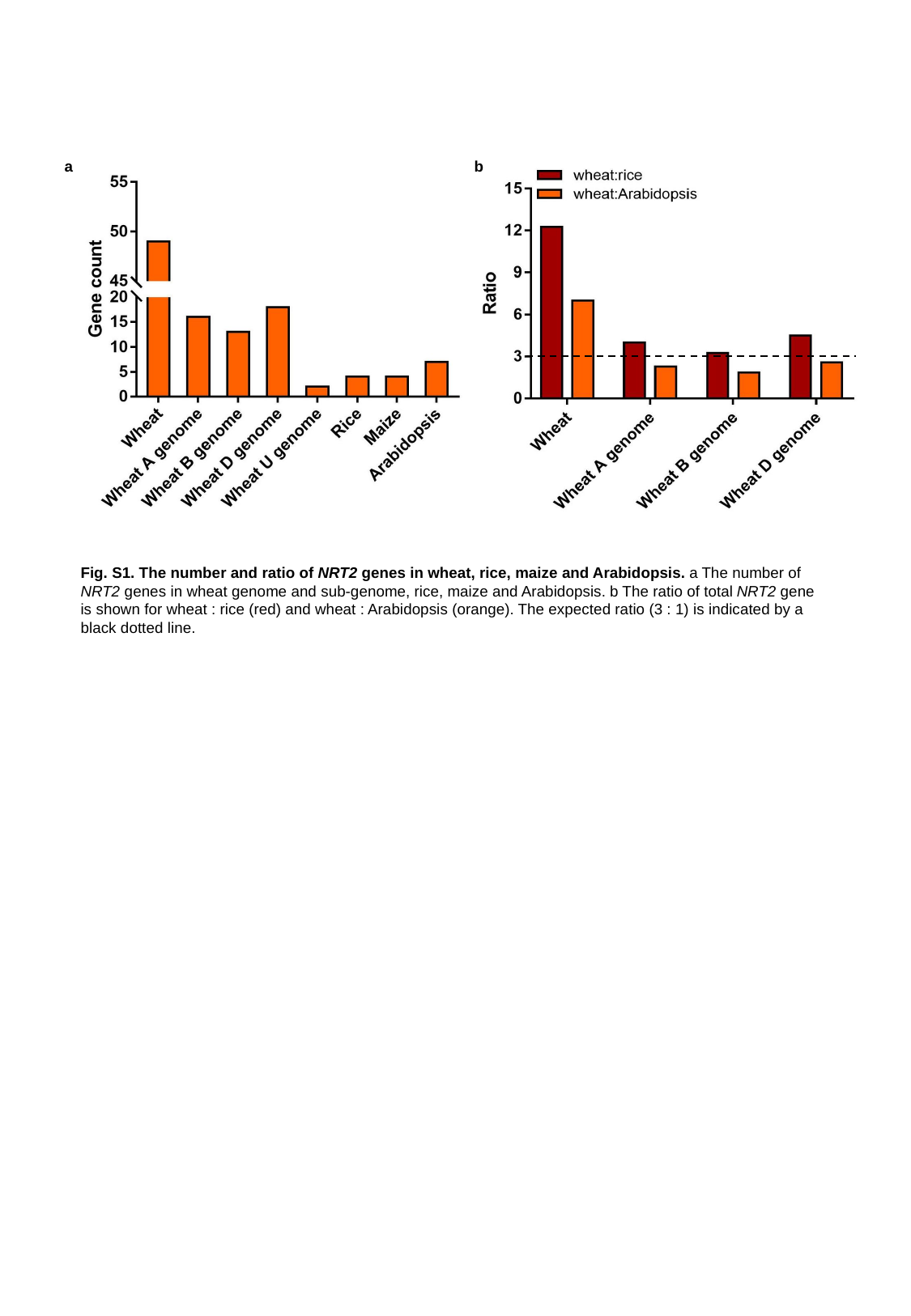

a
b
Fig. S1. The number and ratio of NRT2 genes in wheat, rice, maize and Arabidopsis. a The number of NRT2 genes in wheat genome and sub-genome, rice, maize and Arabidopsis. b The ratio of total NRT2 gene is shown for wheat : rice (red) and wheat : Arabidopsis (orange). The expected ratio (3 : 1) is indicated by a black dotted line.

## Slide 2
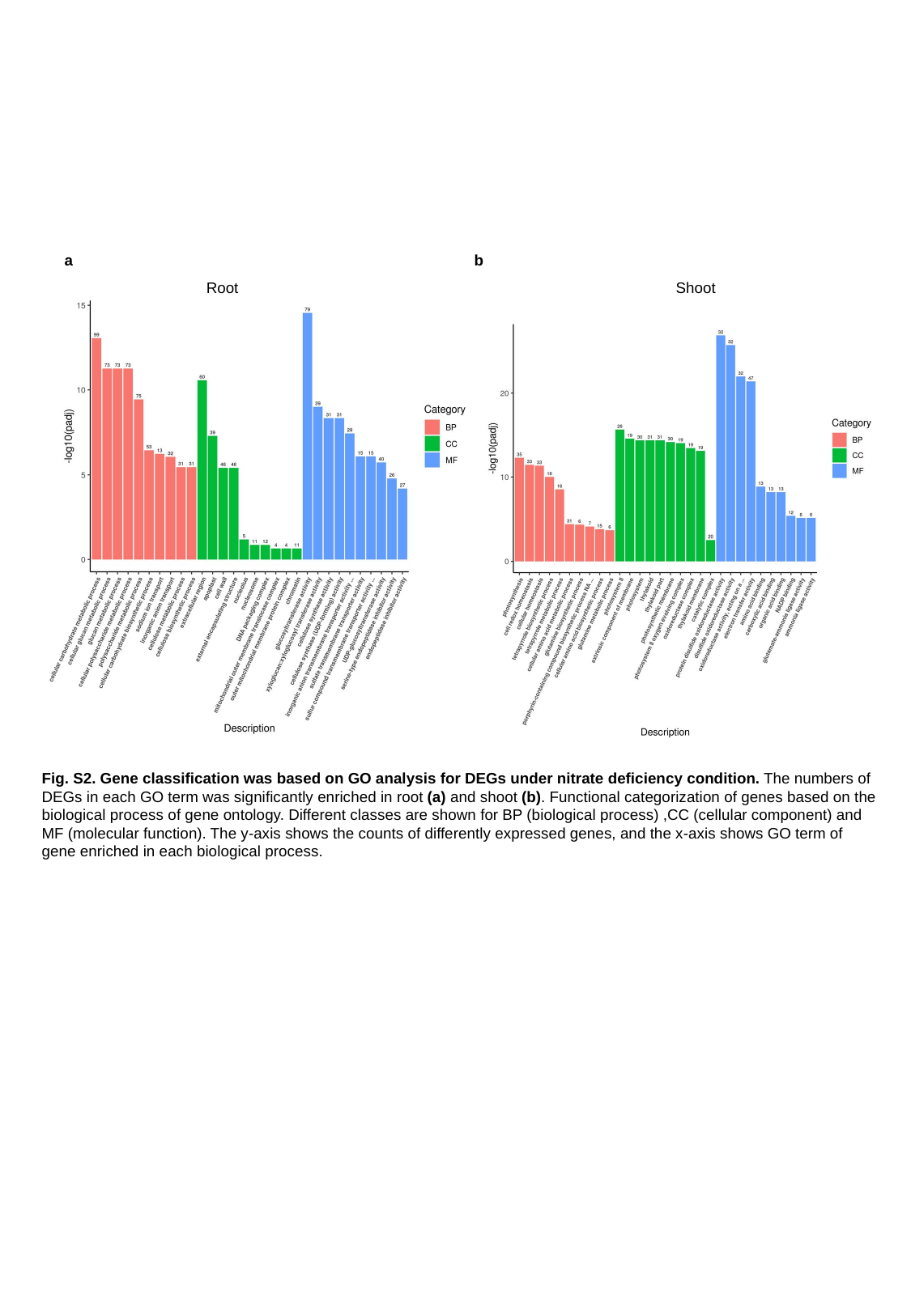

a
b
	Root Shoot
Fig. S2. Gene classification was based on GO analysis for DEGs under nitrate deficiency condition. The numbers of DEGs in each GO term was significantly enriched in root (a) and shoot (b). Functional categorization of genes based on the biological process of gene ontology. Different classes are shown for BP (biological process) ,CC (cellular component) and MF (molecular function). The y‑axis shows the counts of differently expressed genes, and the x‑axis shows GO term of gene enriched in each biological process.

## Slide 3
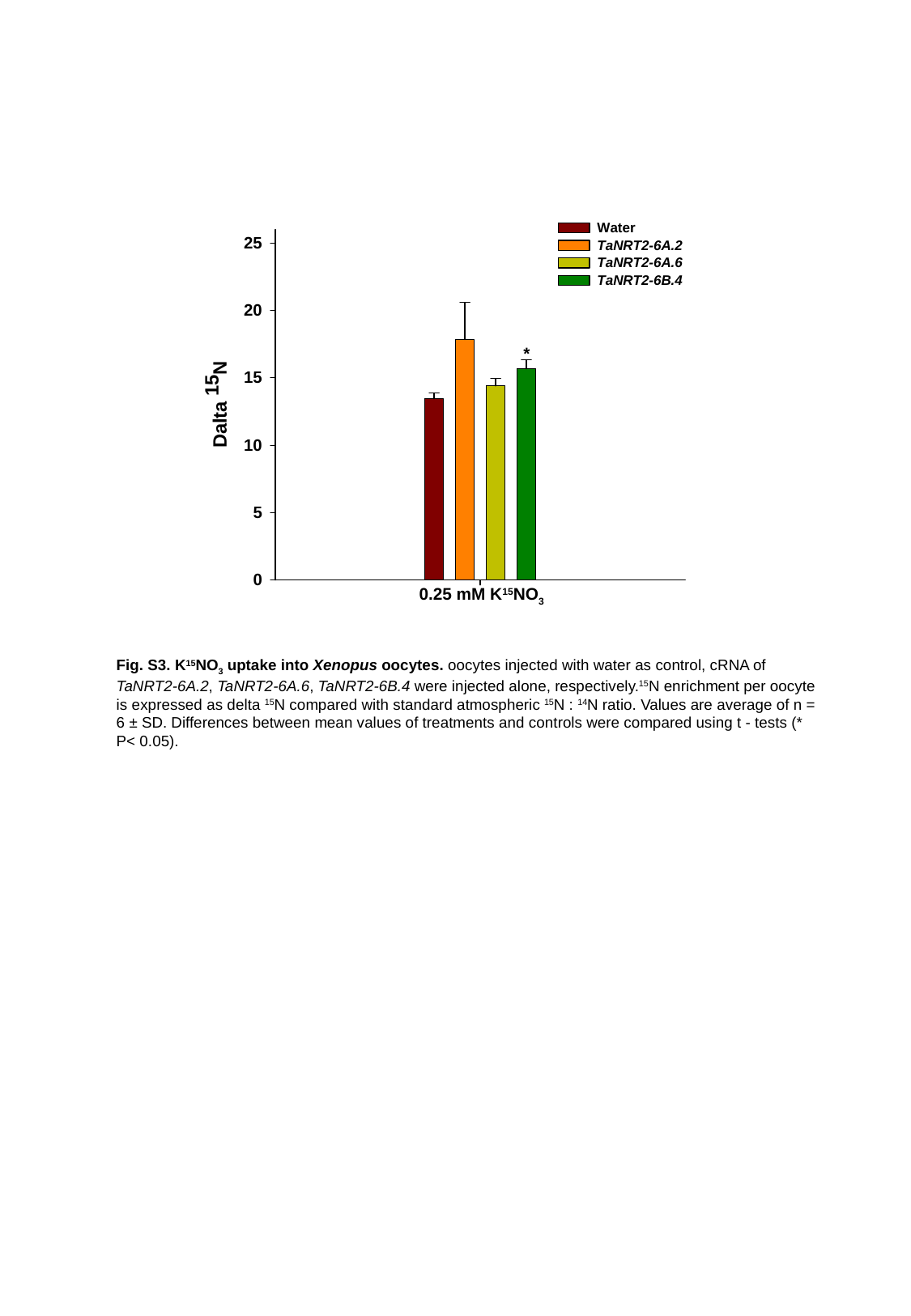

*
0.25 mM K15NO3
Fig. S3. K15NO3 uptake into Xenopus oocytes. oocytes injected with water as control, cRNA of TaNRT2-6A.2, TaNRT2-6A.6, TaNRT2-6B.4 were injected alone, respectively.15N enrichment per oocyte is expressed as delta 15N compared with standard atmospheric 15N : 14N ratio. Values are average of n = 6 ± SD. Differences between mean values of treatments and controls were compared using t - tests (* P< 0.05).

## Slide 4
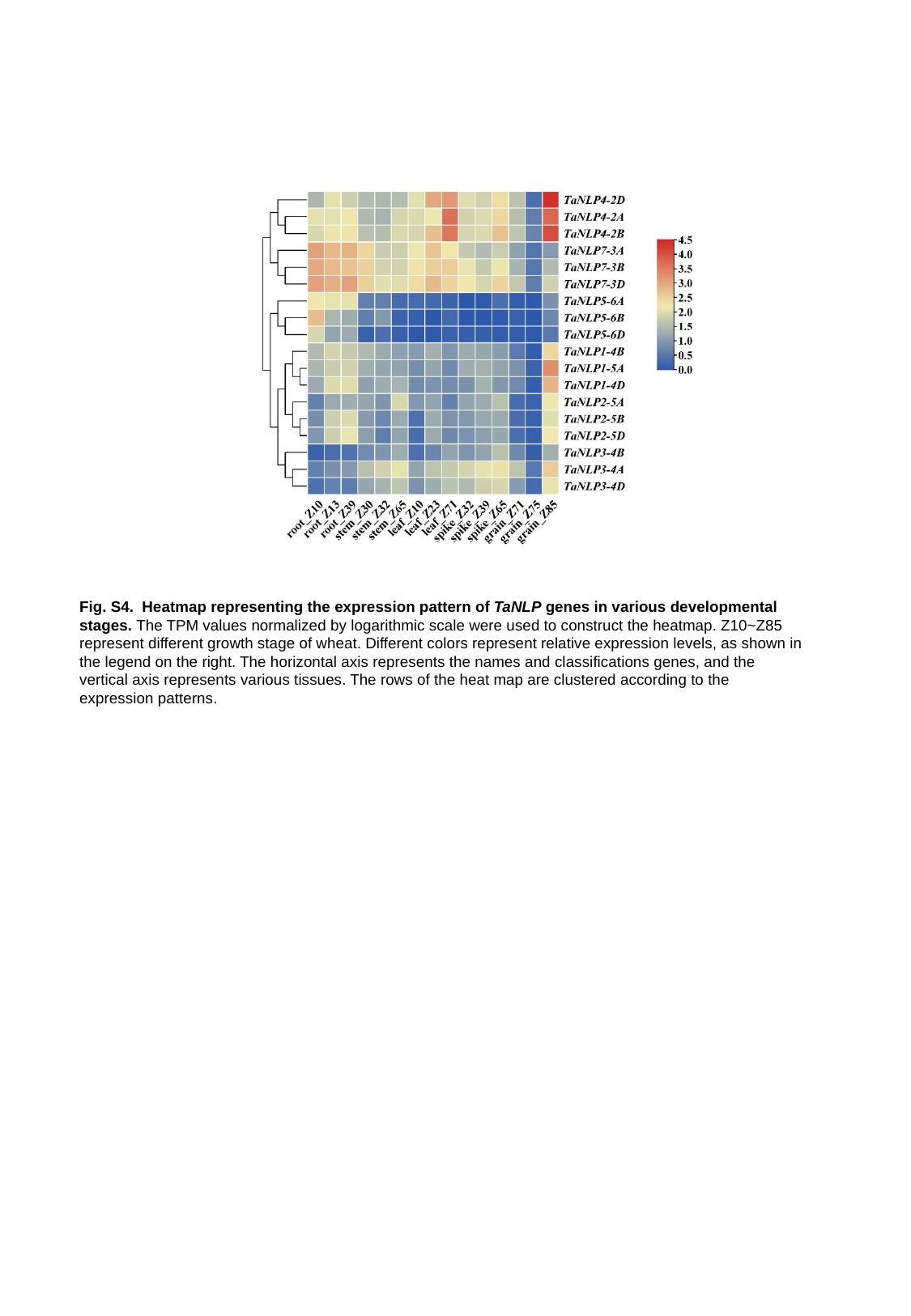

Fig. S4. Heatmap representing the expression pattern of TaNLP genes in various developmental stages. The TPM values normalized by logarithmic scale were used to construct the heatmap. Z10~Z85 represent different growth stage of wheat. Different colors represent relative expression levels, as shown in the legend on the right. The horizontal axis represents the names and classifications genes, and the vertical axis represents various tissues. The rows of the heat map are clustered according to the expression patterns.

## Slide 5
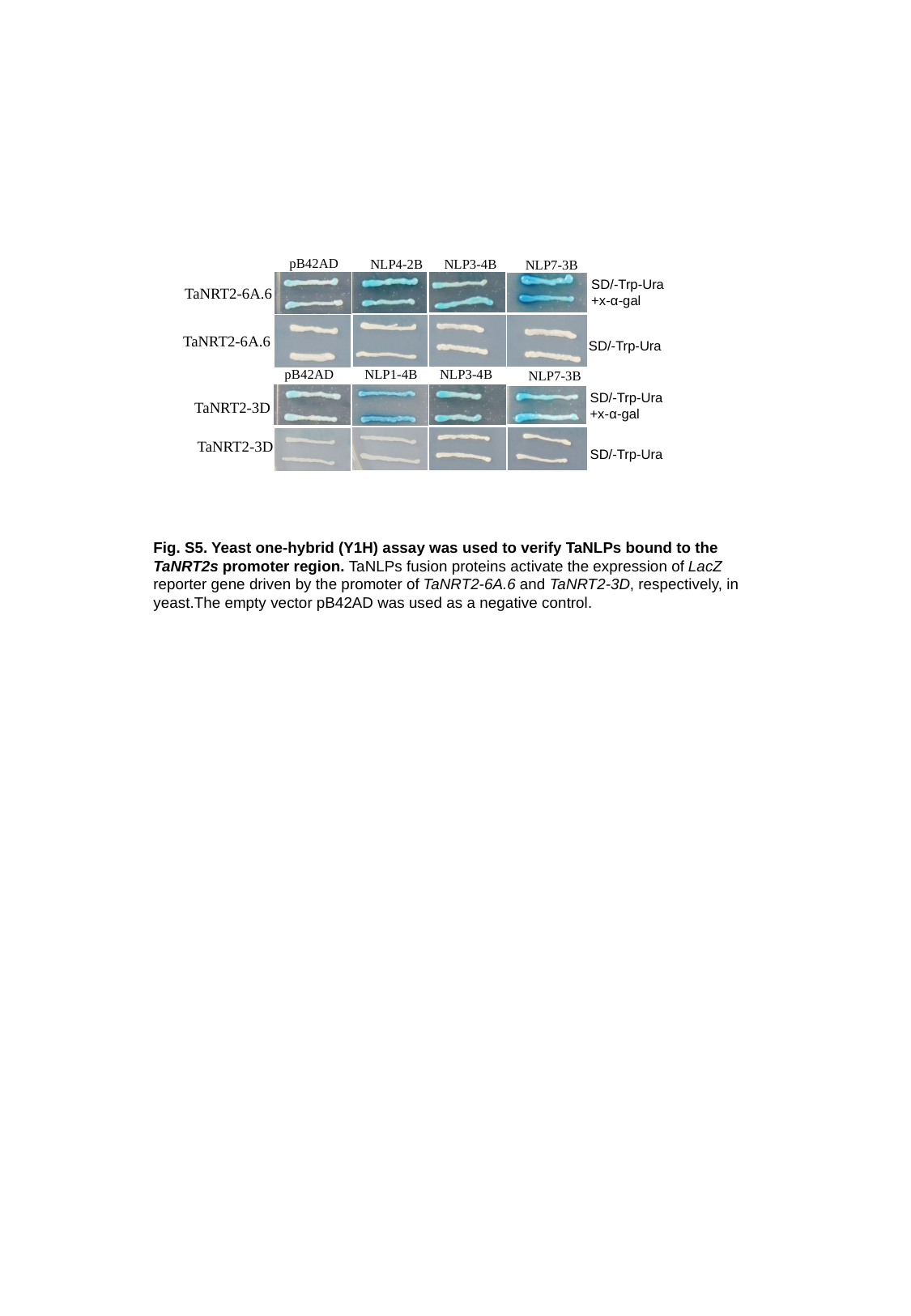

pB42AD
NLP4-2B
NLP3-4B
NLP7-3B
SD/-Trp-Ura
+x-α-gal
TaNRT2-6A.6
TaNRT2-6A.6
SD/-Trp-Ura
NLP1-4B
pB42AD
NLP3-4B
NLP7-3B
SD/-Trp-Ura
+x-α-gal
TaNRT2-3D
TaNRT2-3D
SD/-Trp-Ura
Fig. S5. Yeast one-hybrid (Y1H) assay was used to verify TaNLPs bound to the TaNRT2s promoter region. TaNLPs fusion proteins activate the expression of LacZ reporter gene driven by the promoter of TaNRT2-6A.6 and TaNRT2-3D, respectively, in yeast.The empty vector pB42AD was used as a negative control.

## Slide 6
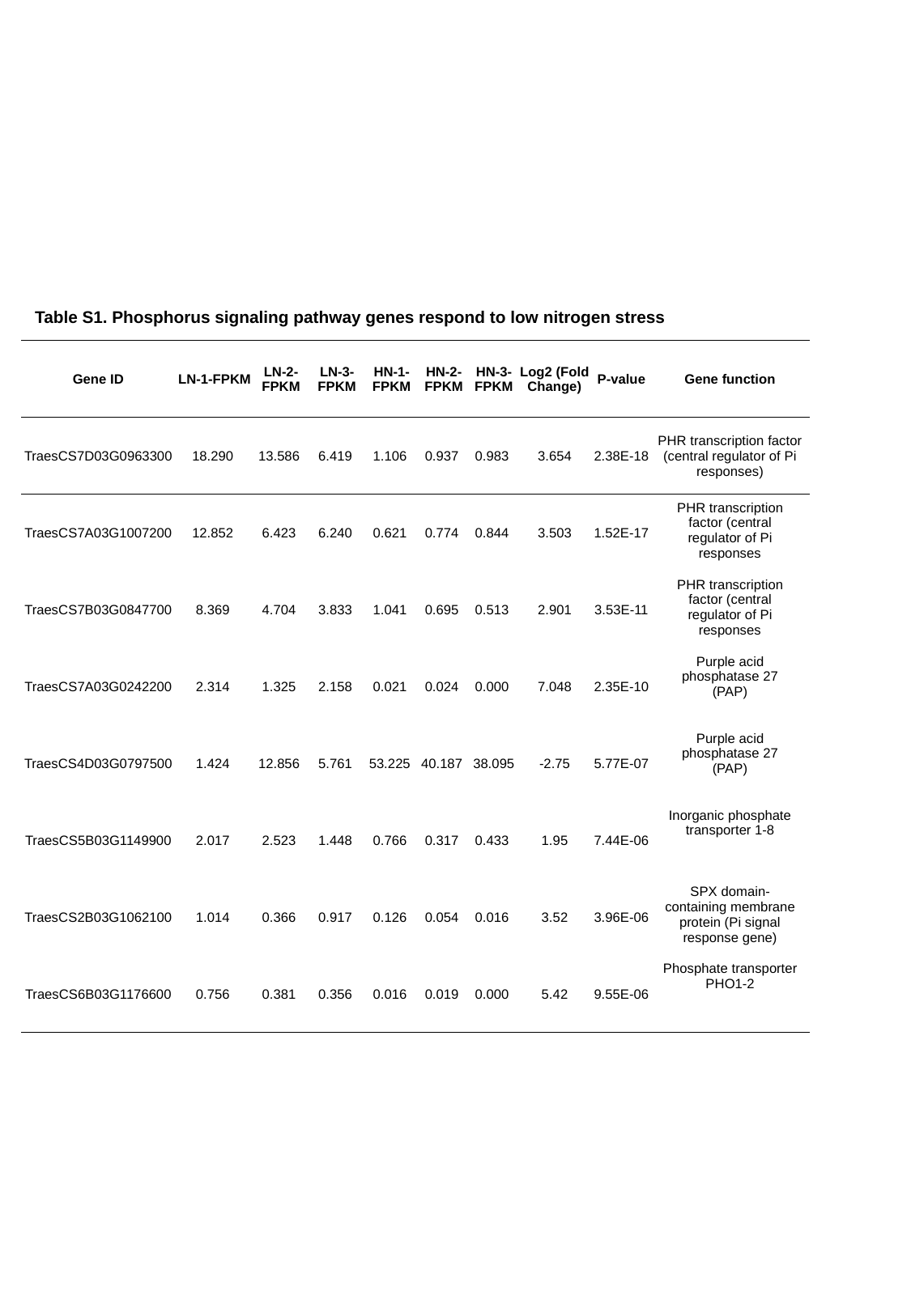

Table S1. Phosphorus signaling pathway genes respond to low nitrogen stress
| Gene ID | LN-1-FPKM | LN-2-FPKM | LN-3-FPKM | HN-1-FPKM | HN-2-FPKM | HN-3-FPKM | Log2 (Fold Change) | P-value | Gene function |
| --- | --- | --- | --- | --- | --- | --- | --- | --- | --- |
| TraesCS7D03G0963300 | 18.290 | 13.586 | 6.419 | 1.106 | 0.937 | 0.983 | 3.654 | 2.38E-18 | PHR transcription factor (central regulator of Pi responses) |
| TraesCS7A03G1007200 | 12.852 | 6.423 | 6.240 | 0.621 | 0.774 | 0.844 | 3.503 | 1.52E-17 | PHR transcription factor (central regulator of Pi responses |
| TraesCS7B03G0847700 | 8.369 | 4.704 | 3.833 | 1.041 | 0.695 | 0.513 | 2.901 | 3.53E-11 | PHR transcription factor (central regulator of Pi responses |
| TraesCS7A03G0242200 | 2.314 | 1.325 | 2.158 | 0.021 | 0.024 | 0.000 | 7.048 | 2.35E-10 | Purple acid phosphatase 27 (PAP) |
| TraesCS4D03G0797500 | 1.424 | 12.856 | 5.761 | 53.225 | 40.187 | 38.095 | -2.75 | 5.77E-07 | Purple acid phosphatase 27 (PAP) |
| TraesCS5B03G1149900 | 2.017 | 2.523 | 1.448 | 0.766 | 0.317 | 0.433 | 1.95 | 7.44E-06 | Inorganic phosphate transporter 1-8 |
| TraesCS2B03G1062100 | 1.014 | 0.366 | 0.917 | 0.126 | 0.054 | 0.016 | 3.52 | 3.96E-06 | SPX domain-containing membrane protein (Pi signal response gene) |
| TraesCS6B03G1176600 | 0.756 | 0.381 | 0.356 | 0.016 | 0.019 | 0.000 | 5.42 | 9.55E-06 | Phosphate transporter PHO1-2 |

## Slide 7
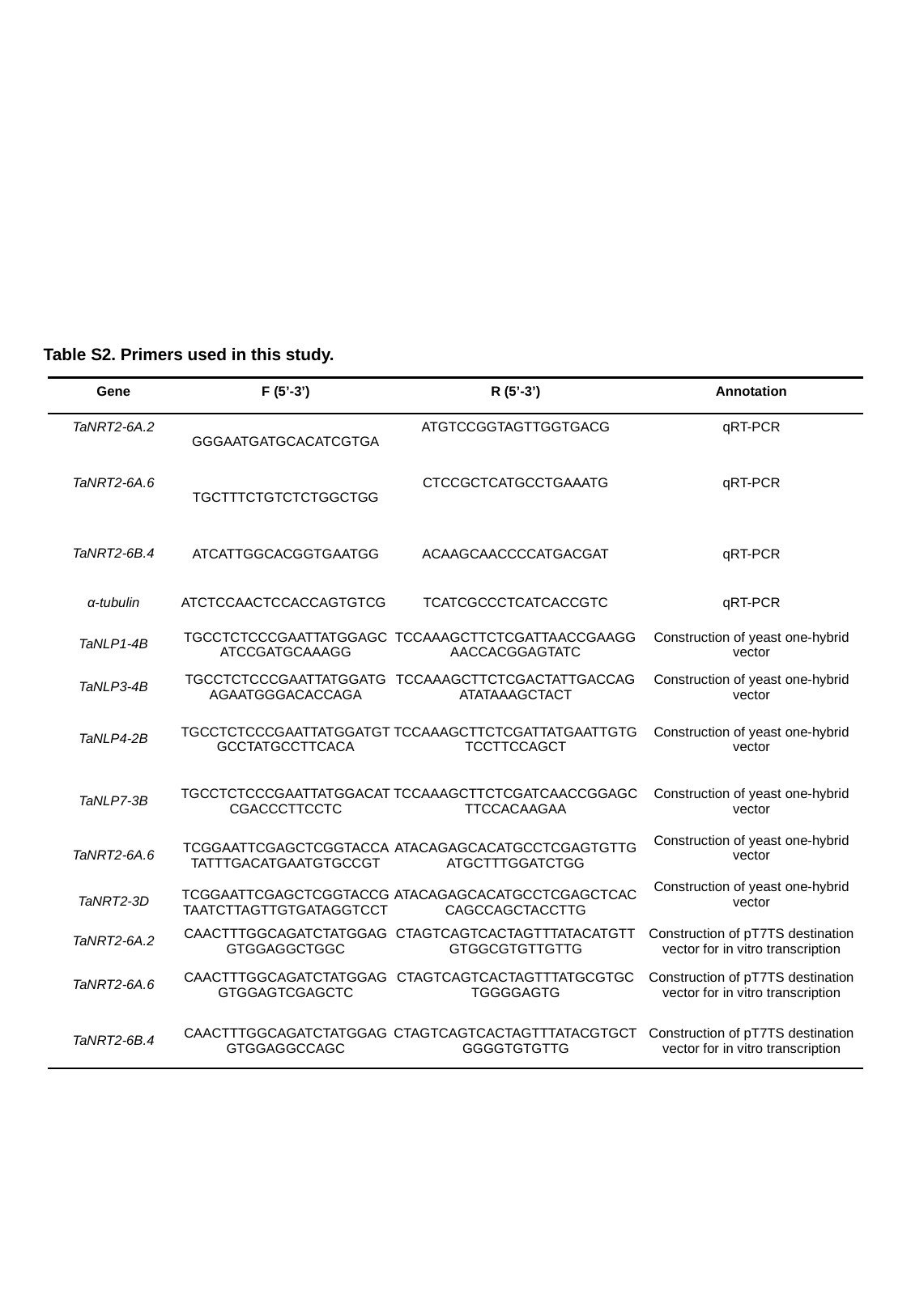

Table S2. Primers used in this study.
| Gene | F (5’-3’) | R (5’-3’) | Annotation |
| --- | --- | --- | --- |
| TaNRT2-6A.2 | GGGAATGATGCACATCGTGA | ATGTCCGGTAGTTGGTGACG | qRT-PCR |
| TaNRT2-6A.6 | TGCTTTCTGTCTCTGGCTGG | CTCCGCTCATGCCTGAAATG | qRT-PCR |
| TaNRT2-6B.4 | ATCATTGGCACGGTGAATGG | ACAAGCAACCCCATGACGAT | qRT-PCR |
| α-tubulin | ATCTCCAACTCCACCAGTGTCG | TCATCGCCCTCATCACCGTC | qRT-PCR |
| TaNLP1-4B | TGCCTCTCCCGAATTATGGAGCATCCGATGCAAAGG | TCCAAAGCTTCTCGATTAACCGAAGGAACCACGGAGTATC | Construction of yeast one-hybrid vector |
| TaNLP3-4B | TGCCTCTCCCGAATTATGGATGAGAATGGGACACCAGA | TCCAAAGCTTCTCGACTATTGACCAGATATAAAGCTACT | Construction of yeast one-hybrid vector |
| TaNLP4-2B | TGCCTCTCCCGAATTATGGATGTGCCTATGCCTTCACA | TCCAAAGCTTCTCGATTATGAATTGTGTCCTTCCAGCT | Construction of yeast one-hybrid vector |
| TaNLP7-3B | TGCCTCTCCCGAATTATGGACATCGACCCTTCCTC | TCCAAAGCTTCTCGATCAACCGGAGCTTCCACAAGAA | Construction of yeast one-hybrid vector |
| TaNRT2-6A.6 | TCGGAATTCGAGCTCGGTACCATATTTGACATGAATGTGCCGT | ATACAGAGCACATGCCTCGAGTGTTGATGCTTTGGATCTGG | Construction of yeast one-hybrid vector |
| TaNRT2-3D | TCGGAATTCGAGCTCGGTACCGTAATCTTAGTTGTGATAGGTCCT | ATACAGAGCACATGCCTCGAGCTCACCAGCCAGCTACCTTG | Construction of yeast one-hybrid vector |
| TaNRT2-6A.2 | CAACTTTGGCAGATCTATGGAGGTGGAGGCTGGC | CTAGTCAGTCACTAGTTTATACATGTTGTGGCGTGTTGTTG | Construction of pT7TS destination vector for in vitro transcription |
| TaNRT2-6A.6 | CAACTTTGGCAGATCTATGGAGGTGGAGTCGAGCTC | CTAGTCAGTCACTAGTTTATGCGTGCTGGGGAGTG | Construction of pT7TS destination vector for in vitro transcription |
| TaNRT2-6B.4 | CAACTTTGGCAGATCTATGGAGGTGGAGGCCAGC | CTAGTCAGTCACTAGTTTATACGTGCTGGGGTGTGTTG | Construction of pT7TS destination vector for in vitro transcription |
